# Global Regulatory Functions of the *Staphylococcus aureus* Endoribonuclease III in Gene Expression

**DOI:** 10.1371/journal.pgen.1002782

**Published:** 2012-06-28

**Authors:** Efthimia Lioliou, Cynthia M. Sharma, Isabelle Caldelari, Anne-Catherine Helfer, Pierre Fechter, François Vandenesch, Jörg Vogel, Pascale Romby

**Affiliations:** 1 Architecture et Réactivité de l′ARN, Université de Strasbourg, CNRS, IBMC, Strasbourg, France; 2 Zentrum für Infektionsforschung (ZINF), Würzburg, Germany; 3 Inserm U851, Centre National de Référence des Staphylocoques, Université de Lyon, Lyon, France; 4 Institut für Molekulare Infektionsbiologie, Würzburg, Germany; Uppsala University, Sweden

## Abstract

RNA turnover plays an important role in both virulence and adaptation to stress in the Gram-positive human pathogen *Staphylococcus aureus*. However, the molecular players and mechanisms involved in these processes are poorly understood. Here, we explored the functions of *S. aureus* endoribonuclease III (RNase III), a member of the ubiquitous family of double-strand-specific endoribonucleases. To define genomic transcripts that are bound and processed by RNase III, we performed deep sequencing on cDNA libraries generated from RNAs that were co-immunoprecipitated with wild-type RNase III or two different cleavage-defective mutant variants *in vivo*. Several newly identified RNase III targets were validated by independent experimental methods. We identified various classes of structured RNAs as RNase III substrates and demonstrated that this enzyme is involved in the maturation of rRNAs and tRNAs, regulates the turnover of mRNAs and non-coding RNAs, and autoregulates its synthesis by cleaving within the coding region of its own mRNA. Moreover, we identified a positive effect of RNase III on protein synthesis based on novel mechanisms. RNase III–mediated cleavage in the 5′ untranslated region (5′UTR) enhanced the stability and translation of *cspA* mRNA, which encodes the major cold-shock protein. Furthermore, RNase III cleaved overlapping 5′UTRs of divergently transcribed genes to generate leaderless mRNAs, which constitutes a novel way to co-regulate neighboring genes. In agreement with recent findings, low abundance antisense RNAs covering 44% of the annotated genes were captured by co-immunoprecipitation with RNase III mutant proteins. Thus, in addition to gene regulation, RNase III is associated with RNA quality control of pervasive transcription. Overall, this study illustrates the complexity of post-transcriptional regulation mediated by RNase III.

## Author Summary

Control of mRNA stability is crucial for bacteria to survive and rapidly adapt to environmental changes and stress conditions. The molecular players and the degradation pathways involved in these adaptive processes are poorly understood in *Staphylococcus aureus*. The universally conserved double-strand-specific endoribonuclease III (RNase III) in *S. aureus* is known to repress the synthesis of several virulence factors and was recently implicated in genome-wide mRNA processing mediated by antisense transcripts. We present here the first global map of direct RNase III targets in *S. aureus*. Deep sequencing was used to identify RNAs associated with epitope-tagged wild-type RNase III and two catalytically impaired but binding-competent mutant proteins *in vivo*. Experimental validation revealed an unexpected variety of structured RNA transcripts as novel RNase III substrates. In addition to rRNA operon maturation, autoregulation, degradation of structured RNAs, and antisense regulation, we propose novel mechanisms by which RNase III increases mRNA translation. Overall, this study shows that RNase III has a broad function in gene regulation of *S. aureus*. We can now address more specifically the roles of this universally conserved enzyme in gene regulation in response to stress and during host infection.

## Introduction

Bacteria are highly adaptive organisms that are able to rapidly alter their gene expression in response to environmental changes. In addition to transcriptional control, regulation of RNA decay has emerged as a major pathway in fast adaptive processes. Changes in RNA turnover facilitate stress responses, growth phase transitions, and virulence factor production [1]–[3]. Over the past decades, the knowledge on key ribonucleases that act in processing and turnover of RNAs in *Escherichia coli* and *Bacillus subtilis* has increased considerably [1]–[3]. Degradation of mRNA can follow several pathways involving a combination of exo- and endoribonucleases, and differs substantially between Gram-negative and Gram-positive bacteria [3], [4]. For instance, *E. coli* uses the single-strand-specific RNase E to catalyze the initial rate-limiting cleavage of a large number of mRNAs [1], while mRNA decay in *B. subtilis* involves the action of the endoribonuclease RNase Y and the bi-functional RNases J1/J2, which are endowed with 5′ exoribonuclease and endoribonuclease activities [5], [6].

Among the endoribonucleases, ribonuclease III (RNase III) is a member of a highly conserved and universal family of double-stranded-RNA (dsRNA)-specific enzymes with essential roles in RNA processing and decay [1], [3], [7]. The discovery that RNase III-type enzymes generate eukaryotic microRNAs and short interfering RNAs has triggered interest in defining the mechanisms of action of this family [8], [9]. Crystal structures of *Aquifex aeolicus* RNase III in complex with different dsRNAs indicated that this protein contains a long RNA-binding surface cleft denoted the catalytic valley [9], [10]. Bacterial RNase III is a homodimer that forms a single processing center with each subunit contributing to the hydrolysis of one RNA strand. Each monomer contains four RNA binding motifs that make extensive contact with the ribose-phosphate of the dsRNA up to 10 base pairs from the cleavage site, while conserved acidic amino acids and Mg^2+^ are responsible for catalysis [9], [11]. Biochemical studies have identified the determinants of the dsRNA substrate and RNase III that are required for substrate specificity and catalytic activity. RNase III cleavage produces RNA fragments with 5′-phosphate and 3′-hydroxyl termini and a two-nucleotide 3′-overhang [11]–[14]. Aside from the universal function of RNase III in the maturation of ribosomal RNAs [15], *E. coli* RNase III plays a broad role in gene regulation. Not only does RNase III autoregulate its own synthesis [16], it also contributes to regulation by small RNAs [17], [18]. In addition, recent genomic analyses revealed that the absence of RNase III in *E. coli* [19] and *B. subtilis* [20] affects the abundance of numerous mRNAs and non-coding RNAs (ncRNAs).

Did the cellular functions and substrate specificity of the ubiquitous RNase III diverge in Gram-positive bacteria? In *Streptococcus pyogenes*, RNase III was identified as an essential host factor for the prokaryotic CRISPR/Cas immunity system [21]. In *B. subtilis*, the *rnc* gene is essential suggesting that RNase III-dependent maturation of one or several critical mRNAs is required for protein synthesis [20], [22]. In *Staphylococccus aureus*, an *rnc* mutant strain showed compromised virulence in a murine peritonitis model [23], while *rnc* deletion did not impair cell growth [23], [24]. Our previous studies in *S. aureus* have shown that RNase III coordinates the repression of mRNAs encoding virulence factors and a transcriptional regulator via the quorum-sensing-dependent regulatory RNA, RNAIII [24]–[26]. The RNAIII-target mRNA complexes adopt various topologies, such as imperfect duplexes and loop-loop interactions that are efficiently recognized and cleaved by RNase III, thus leading to irreversible repression [27]. In addition, a very recent study has shown an unprecedented role of RNase III in antisense regulation restricted to Gram-positive bacteria [28]. Deep sequencing of short *S. aureus* RNAs revealed numerous 22-nt RNA fragments generated by RNase III digestion of sense/antisense RNAs and almost 75% of the cleaved mRNAs had corresponding antisense RNAs [28]. These data are indicative of pervasive antisense regulation by RNase III. Collectively, the previous studies in *E. coli* [19], *B. subtilis* [20], and *S. aureus* [28] evaluated the role of RNase III at a genome-wide scale. However, these analyses of transcriptome changes by tiling array or RNA-seq are not *per se* suitable to identify direct RNase III substrates because they also score indirect regulatory effects. This prompted us to more precisely analyze the functions and direct targets of *S. aureus* RNase III in gene regulation.

We present here the first global map of direct RNase III targets in *S. aureus*. To this end, we used deep sequencing to identify RNAs associated with epitope-tagged wild-type RNase III and two catalytically impaired but binding-competent mutant proteins. Newly identified RNase III targets were validated by a combination of *in vivo* and *in vitro* approaches. Our analysis revealed an unexpected variety of structured RNA transcripts as novel RNase III substrates. In addition to rRNA operon maturation, autoregulation of *rnc* mRNA decay, degradation of structured RNA transcripts, and antisense regulation, we propose novel mechanisms by which RNase III activates the translation of mRNAs through *cis*- or *trans*-acting elements. Overall, our study explores the broad function of RNase III in gene regulation of *S. aureus*.

## Results

### Mutations in *S. aureus* RNase III uncouple binding and catalytic activities

**Figure 1:**
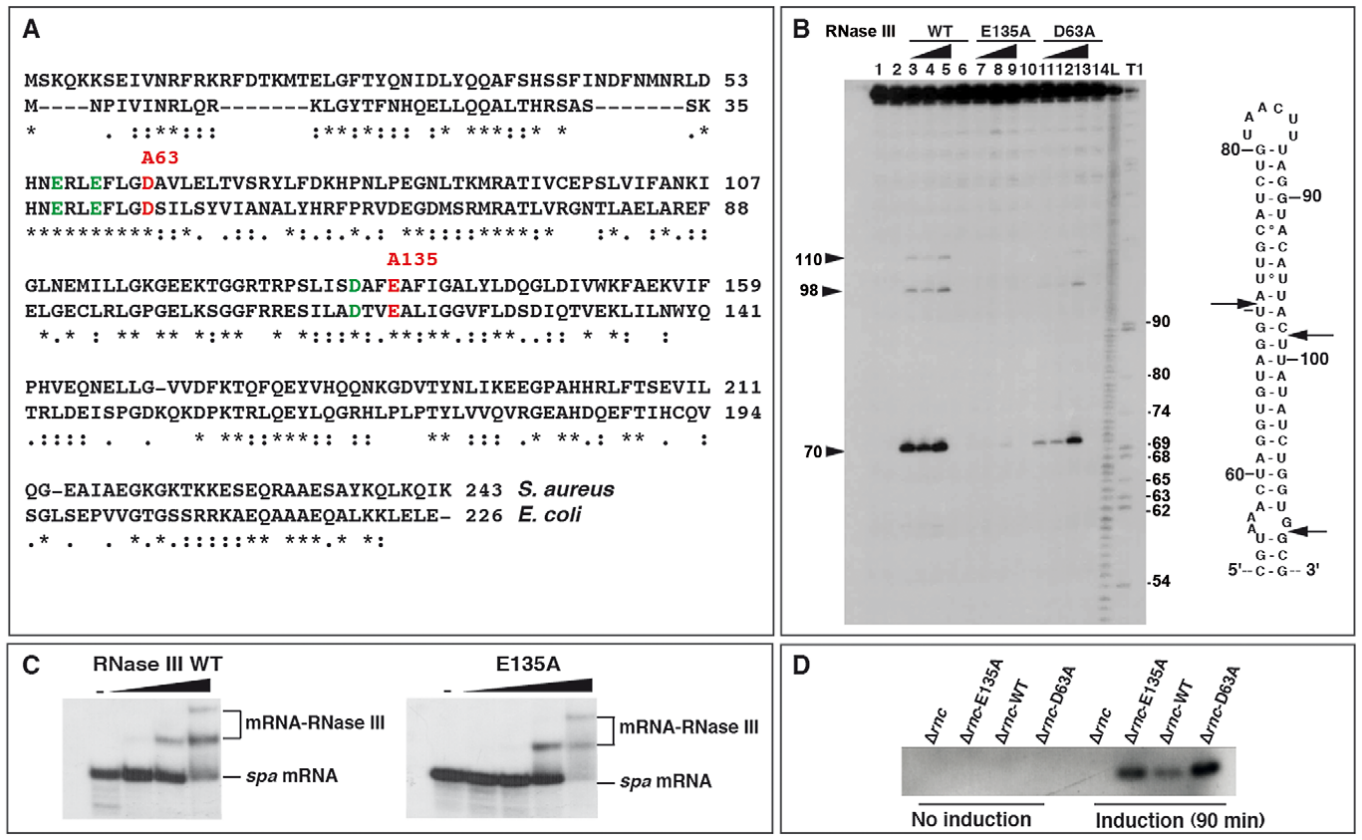
Effect of mutations in the catalytic site of *Staphylococcus aureus* RNase III. (A) Amino acid sequence alignment of RNase III from *S. aureus* and *Escherichia coli*. Acidic amino acids colored in green and red are conserved residues present in the catalytic site of *E. coli* RNase III. The two mutations (D63 to A and E135 to A) generated in *S. aureus* RNase III are shown in red. (B) RNase III cleavage assays were performed on 5′ end-labeled *spa* mRNA using the wild-type enzyme (RNase III-wt), the E135A, and the D63A RNase III mutant enzymes. Lanes 1, 2: incubation controls set in the absence of RNase III. Cleavage reactions were performed in the presence of Mg^2+^ with increasing concentrations of RNase III WT (lanes 3–5), the E135A (lanes 7–9) or D63A (lanes 11–13) mutant proteins: (lanes 3, 7, 11) 0.165 µM; (lanes 4, 8, 12) 0.33 µM; (lanes 5, 9, 13) 0.66 µM. Control reactions included Ca^2+^ and 0.66 µM of RNase III WT (lane 6), E135A (lane 10) or D63A (lane 14) mutants. Lanes T, L: RNase T1 and alkaline ladders, respectively, under denaturing conditions. Arrows denote the positions of RNase III cleavages which are shown in the secondary structure of the RNase III-binding site on *spa* mRNA. (C) Binding of the wild-type (RNase III-wt) and mutant (E135A) enzymes to 5′ end-labeled *spa* mRNA as visualized by gel retardation assays. Lane (−): incubation control of the free RNA in the absence of RNase III. Increasing concentrations of RNase III WT (0.05, 0.1 and 0.2 µM) or RNase III-E135A (0.08, 0.16, 0.32 and 0.49 µM) were added to 5′ end-labeled *spa* mRNA in a buffer containing Ca^2+^ instead of Mg^2+^. (D) RNase III-Flag protein levels at mid-logarithmic phase were monitored in various *S. aureus* strains: Δ*rnc* mutant strain, Δ*rnc* mutant strain complemented with the Flag-E135A mutant, the Flag-wt RNase III and the Flag-D63A mutant. Cells were collected before or after induction with CdCl_2_ for 90 min. 40 µg of total protein were resolved on an SDS-PAGE gel. The western blot was performed with an anti-Flag monoclonal antibody.

Biochemical and structural studies performed on RNase III in *A. aeolicus* and *E. coli* demonstrated a stepwise hydrolysis mechanism of the phosphodiester bonds mediated by two Mg^2+^ ions, involving mutual conformational changes of the RNA and the enzyme [10], [29]. The nuclease domain of RNase III is characterized by two clusters of conserved acidic amino acids, in which the side chains of E41, D45, D114, and E117 (in *E. coli*) are coordinated to Mg^2+^ ions [13], [30], [31]. Two of these residues, E117 and D45, are essential for catalysis, as their substitution by alanine strongly compromised cleavage without affecting RNA binding [11], [14], [29], [31]. Although *S. aureus* RNase III (Sa-RNase III) shares only 33% amino acid identity with the *E. coli* enzyme, the acidic amino acids are strictly conserved (Figure 1A). To obtain catalytically inactive but binding-proficient variants of Sa-RNase III, amino acids E135 and D63 (corresponding to E117 and D45 in *E. coli*, respectively) were changed to alanine (Figure 1A). A histidine epitope tag was added to the N-terminus of the mutant and wild-type (WT) proteins, and the proteins were purified to homogeneity following expression in *E. coli* [27]. The activities of the mutant enzymes were compared to that of the WT protein using *spa* mRNA, a well-characterized Sa-RNase III substrate [24]. Terminally labeled *spa* mRNA was used to map the cleavage sites for WT and mutant *S. aureus* proteins and cleaved products were resolved by polyacrylamide gel electrophoresis under denaturing conditions (Figure 1B). As expected, WT RNase III cleaved both sides of a helix at U70, C98, and G110 in the coding sequence (CDS) of *spa* mRNA. The E135A mutation very strongly compromised the activity of the enzyme, while the effect of D63A was less pronounced (Figure 1B). Gel retardation assays were used to monitor the binding of the mutant enzymes to terminally labeled *spa* mRNA in a buffer containing Ca^2+^ instead of Mg^2+^ (Figure 1C). Ca^2+^ inhibits the catalytic activity of *E. coli* RNase III but does not affect RNA binding [32]. In our study, the mutant E135A (Figure 1C) and D63A (result not shown) enzymes bound *spa* mRNA in a manner similar to the WT RNase III. Hence, the two mutations uncoupled catalytic activity from RNA binding capacity in a manner similar to that described for *E. coli* RNase III [11], [14], [29]. These two mutant proteins were used to capture RNA substrates *in vivo*.

### Identification of several classes of structured RNAs associated with RNase III

To identify RNase III targets *in vivo*, co-immunoprecipitation (coIP) assays were carried out with Flag epitope-tagged WT and mutant proteins expressed from a plasmid-borne Cd^2+^-inducible promoter in a Δ*rnc S. aureus* background. The Flag-epitope was added to the C-terminus of the proteins. A control coIP experiment was performed with the untagged WT RNase III expressed from the chromosome (strain RN6390). Bacteria were harvested at two time points (4 and 6 h of growth at 37°C) corresponding to exponential and late exponential phases of growth, respectively. The growth curves of RN6390 (WT strain), Δ*rnc* mutant strain, and Δ*rnc* complemented with WT RNase III, E135A, or D63A mutant proteins, were similar in the BHI medium (data not shown). Western blot analysis showed that the two mutant proteins accumulated at comparable levels while the WT protein was expressed at a lower level (Figure 1D), indicative of a possible autoregulatory event on the *rnc* mRNA.

RNAs isolated from the coIP experiments with the four strains were converted to cDNA libraries and analyzed by high-throughput pyrosequencing as previously described [33]. Recovered sequences ranged from 1 to 145 nt, but sequences below 18 nt were discarded in later analyses to increase the accuracy of mapping (Table S1). In agreement with the impaired catalytic activity of the mutants, we obtained more reads of ≥18 bp with the E135A (77% of the total reads) and D63A (86%) mutant proteins than with WT RNase III (51%). Mapping of the cDNA reads to the genome of *S. aureus* N315 revealed that the mutant enzymes primarily recovered RNA fragments arising from rRNA and tRNA operons (80 to 90% of the total number of mapped reads) (Table S2). However, a high number of reads were mapped to 58 different ncRNAs including the housekeeping ncRNAs tmRNA, RNase P, and the RNA component (4.5S RNA) of the signal recognition particle (SRP) (Tables S2, S3). Furthermore, in the coIPs of the mutant enzymes, reads were recovered for almost 1,500 individual mRNAs of the 2,653 annotated ORFs in the *S. aureus* genome, but considerably fewer were recovered for polycistronic mRNAs (*mnhA-G*, *gapR*, *pdhA-D*, and *qox*; Table S4). Moreover, a significant number of reads corresponded to antisense RNAs (asRNAs) that were assigned to the opposite strand of 1,175 mRNAs (Tables S1, S5). Given the limited number of sequenced cDNAs (∼1×10^5^) (Table S1), the actual number of RNase III targets may be underestimated. Based on comparison to sequences from the control coIP (WT strain expressing untagged RNase III), which should represent RNAs that are unspecifically bound during the coIP, we only considered transcripts as potential RNase III substrates if they were significantly enriched in the coIP samples of the tagged variants (Tables S2, S3, S4, S5). Not surprisingly, the RNAs that were unspecifically bound in the control coIP reflect the abundance of the transcripts in the cell, i.e., most of these reads were derived from rRNAs that represent >90% of the transcripts in the cell (Table S2). We note that the co-immunoprecipitated RNAs identified with the E135A and D63A mutant proteins were very similar, supporting the reliability and reproducibility of the method (Tables S2, S3, S4, S5). Moreover, many of the target RNAs were detected at both time points of cell growth. Representatives of each RNA class were then selected for experimental validation using *in vitro* and *in vivo* approaches (Table 1).

**Table 1:** Validation of several RNA targets of RNase III.

5′end	3′end	St^a^	Annotation/Alternative IDs	Adjacent genes/Orientation	Comments^b^	Ref^c^	Northern/mRNA stability exp	*In vitro* RNase III cleavage assay	Binding
**ncRNAs**									
292655	292779	−	RsaX28	SA0240><>SA0241			nd		
413160	413255	−	RsaL, SAU-5971	*rpsR*><<SA0355	Processed from large 3′UTR of SA0355	(1)	Highly expressed		
436954	437055	−	RsaM, Teg146, SAU63b	SA0377<<<SA0378	Processed from large 3′UTR of SA0378	(1,2)	Highly expressed		
501414	501696	+	4.5S	SA0434>>>SA0435	4.5S RNA	(2,3,4)	Highly expressed	N	Y
511520	511626	+	RsaX29, Teg43	5S rRNA>>>SA0439	Sequence in part similar to 5S rRNA	(4)			
637038	637394	+	RsaA	SA0543<><SA0544	RsaA (short & large transcript)	(1,2,4)	Short and large transcripts less stable in wt than in Δrnc strains	Y, cleavages at the first hairpin	
637016	637429	−	asRsaA	SA0543<<<SA0544	Start in SA0544	(2)	nd		
765830	766139	−	RsaN	SA0673><>SA0674			Detected only in Δ*rnc* strain		
829498	829646	−	asRsaH	SA0724<<>SA0725	Less abundant than RsaH		nd		
829498	829643	+	RsaH	SA0724<>>SA0725		(1,2,4)	Detected in all strains		Y
975383	975574	+	RsaE	SA0859>><SA0860	RsaE, RsaE-RsaF transcripts	(1,2,4)	Detected in all strains	RsaE-RsaF weak cleavages; one at the end of RsaE	
1006734	1006851	−	Teg102bis	SAS028><>SAS029	Sequence partially repeated to the asSAS028	(2,4)			Y
1329666	1329870	−		SA1167><>SA1168	Overlapping Rho-independent terminator with SA1167		nd		Y
1437260	1437392	+	RsaX31	SA1265>>>SA1266	Structured RNA with a UCCCA motif and a Rho-independent terminator. 10 repeats	(4)	nd		
1660498	1660771	−	6S	SA1455<<<*aspS*	SAS050 and 6S RNA (1660650–1660796)	(2,3,4)	Highly expressed	N (6S-SAS050)	Y
2078734	2078872	−	RNAIII	SA1841><>*agrB*	RNAIII (3′ domain)	(5)	Weakly expressed	RNAIII interacts with RNase III but is only cleaved when bound to mRNA targets (*spa*, *rot*)	Y
2108482	2108586	−	RsaX38	*ilvA*><<5S rRNA	5S rRNA like similar to RsaX29	(4)			
2206379	2206710	−	SprG3, Teg19b	SA1956><>SAS069	Hypothetical sORF, antisense to SprF3, 5′ start at 2206385	(3,4)	Highly expressed	SprFG3 hybrid completely degraded	
2206379	2206710	+	SprF3, Teg19a	SA1956>>>SAS069	Antisense to SprG3, 5′ start at 2206708	(2,3,4)	Highly expressed		
2370161	2370254	−	RsaOas	SA2107><>SA2108			nd		
2370165	2370378	+	RsaO	SA2107>>>SA2108	5′ start at 2370165		Weakly expressed		
2421600	2421812	+	RsaX41	SA2155<><SA2156	UCCCA motif	(4)	nd		
**rRNA and tRNA operons**									
549697	555585	+	5S_8tRNAs_16S_tRNA*_23S_5S		rRNA operon		Unprocessed in Δ*rnc* strain	Y	
1152128	1152283	−	tRNA		tRNA+terminator			N	Y
1916277	1919063	−	26tRNAs_ 5S_23S_2tRNAs_16S		rRNA operon		Unprocessed in Δ*rnc* strain		
2109042	2114500	−	5S_23S_16S_2tRNAs		rRNA operon		Unprocessed in Δ*rnc* strain		
2230200	2230809	−	5tRNAs_5S_23S_16S		rRNA operon		Unprocessed in Δ*rnc* strain	tRNA^Tyr^-tRNA^Gln^ cleaved but not at the expected site *in vitro*	Y
**Riboswitches**									
15976	16095	+	5′UTR of SA0011	SAS001>>>SA0011	EL78, SAM riboswitch regulating the synthesis of homoserine-o-acetyl transferase	(2,4)		N*	Y
430802	430906	+	5′UTR of *xrpT*	SA0372<>>*xprT*	Purine riboswitch regulating the synthesis of xanthine phosphoribosyltransferase	(2,4)		N*	
1523781	1523956	−	5′UTR of SA1316	SA1316<<<SA1317	FMN riboswitch regulating the synthesis of SA1316 (hypothetical protein)	(2,4)		N*	Y*
1716104	1716314	−	5′UTR of *thrS*	*thrS*<<<*dnaI*	T-Box regulatory element regulating the synthesis of threonyl-tRNA synthetase	(2,4)		N	
2211998	2212074	−	5′ UTR *glmS*	*glmS*<<>SAS073	GlcN6P riboswitch regulating the synthesis of glucosamine-6 phosphate; EL78; asRNA detected only in EP	(2,4)	mRNA highly expressed in SP but no difference between wt and Δ*rnc* strains		
**mRNAs**									
122506	122736	−	*spa* mRNA	SA0106><<*spa*	CoIP fragments along the mRNA	(6)	More expressed in Δ*rnc* strain	Y	
686734	686982	+	5′UTR *tagG*	*tagH*<>>*tagG*	Overlapping 5′UTR with *tagH* 5′UTR		Detected in Δ*rnc* strain	*tagG/tagH* hybrid completely degraded	
686682	686968	−	5′UTR *tagH*	*tagH*<<>*tagG*	Overlapping 5′UTR with *tagG* 5′UTR		Detected in Δ*rnc* strain		
716327	716597	+	asSA0620	SA0619>>>SA0621	CoIP fragment covering the entire gene		nd		
716027	716571	−	SA0620		SsaA homologue; entire gene		nd		
827582	827930	+	*clpP* mRNA				Moderately expressed	N	
828123	828309	−	as*clpP*	*clpP*><<SA0724	CoIP small fragments complementary to *clpP* mRNA		nd		
831523	839012	+	operon *gapR-gap-pgk-tpiA-pgm-eno*		CoIP fragments covering all mRNAs within the operon		Enolase: highly expressed		
1069600	1069996	+	5′UTR SA0943	*pdf1*<>>SA0943	Overlapping 5′UTR with *pdf1* 5UTR		5′UTR only detected in Δ*rnc* strain; mRNA detected in wt/rnc strains		
1069717	1069907	−	5′UTR-mRNA *pdf1*	*pdf1*<<>SA0943	Overlapping 5′UTR with SA0943 5′UTR; *pdf1* mRNA accumulation in SP		*pdf1* mRNA unprocessed in Δ*rnc* strain		
1215989	1216195	+	*hmrB* mRNA		CoIP fragment including the Rho-independant terminator			N	
1216270	1216395	+	*rnc* mRNA		CoIP fragment within the coding sequence of *rnc* mRNA, 5′ start at 1216196		RNase III cleavage within the coding region	Y	Y
1408765	1409144	−	*cspA* mRNA		CoIP fragment covering the 5′UTR of *cspA*, 5′ start at 1409085 in wt strain		Unprocessed in Δ*rnc* strain	Y	Y
1409014	1409147	+	as*cspA*	*cspA*<><SA1235	EL78; more enriched in SP 5′ start at 1409015		Very weakly expressed	Y; also *cspA/as-cspA* hybrid completely degraded	
1510679	1511027	−	*hu* mRNA		5′ start at 1511027		Highly expressed, increased steady state level in Δ*rnc* mutant	N	Y
1510923	1511030	+	as*hu*	*hu*<><*gpsA*	EL80; more enriched in SP		Weakly expressed		
2074084	2074375	−	*groES* mRNA		EL80		Accumulation of large transcript (operon) in EL78, EL79, EL80	N	
2081487	2082375	−	as*agrA*		EL80; CoIP fragment covers the whole mRNA		nd		
2081528	2082221	+	*agrA* mRNA		CoIP fragments along *agrA* mRNA		Upregulated in SP, no significant difference between wt and Δ*rnc* strains		
2298654	2298870	−	*secY* mRNA		CoIP fragment within the coding region of *secY* mRNA		Highly expressed, more stable in Δ*rnc* strain	Y	Y
2298909	2298991	+	as *secY*		EL80		nd		
2421872	2422221	−	SA2156		*lctP* homologue; entire gene covered			Y, weak cleavages; degradation?	
**Hypothetical small ORFs**									
954606	954882	−	SAS025	SA0841><>SA0842			nd	N	Y
2000661	2000937	−	SAS057	SA1748<<>SA1749				N	
1006325	1006481	+	SAS028	SA0885>>>SAS029	SAS028: hypothetical protein similar to lactococcin 972	(1,2)	Highly expressed, steady state level higher in Δ*rnc* strain than in the wt strain	Y; weak cleavages; degradation?	
1006234	1006479	−	asSAS028/SAU-02/teg102	SA0885><>SAS029	Partially repeated with Teg102bis RNA, 5′ start at 1409015	(1,2)	Less abundant than SAS028 mRNA		

The annotation of the genome is taken from strain N315.

(a) Strand (+/−) of the genome;

(b) Experiments were performed in RN6390 strain and the mutant Δ*rnc* strain transformed with a plasmid expressing the wt enzyme (wt, strain EL79), the mutant D63A (strain EL80), or E135A enzyme (strain EL78); nd is for not detected.

(c) References: (1) Abu-Qatouseh et al. [36]; (2) Beaume et al. [37]; (3) Pichon and Felden [42]; (4) Geissmann et al. [43]; (5) Novick et al. [44]; (6) Huntzinger et al. [23]. N: No cleavage or binding detected; Y: cleavage (or binding) detected.

*: Assays were done in the presence or absence of the respective ligand. RNase III cleavage and binding assays were done as described in Materials and Methods.

We first performed gel retardation assays to validate a direct interaction between various classes of RNAs and the mutant E135A protein. The data showed that the E135A protein bound to a plethora of structured RNAs including *cis*-acting regulatory elements of mRNAs (e.g., the flavin mononucleotide (FMN) sensing riboswitch), ncRNAs, structured mRNAs, and small ORF-containing RNAs (Figure S1A). Competition binding assays were also performed to monitor the specificity of RNase III binding on *cspA* mRNA. Two forms of *cspA* mRNAs were analyzed: *cspA_L_* containing a long 5′UTR (113 nt), which was recovered with the two mutant proteins and *cspA_S_* containing a short 5′UTR (52 nt), which was pulled down with the WT enzyme. We also used SA2097 mRNA, which was not co-immunoprecipitated with the mutant and WT RNase III. The experiments were carried out with the 5′ end-labeled *cspA_L_* mRNA bound to E135A mutant protein in the presence of increasing concentrations of cold *cspA_L_*, *cspA_S_* or SA2097 mRNA (Figure S1B). The experiments showed that the concentrations of *cspA_S_* and SA2097 necessary to compete for binding were 10 times higher than that for *cspA_L_* suggesting that the interaction of RNase III with *cspA_L_* is specific.

Overall, the data strongly suggest that the immunoprecipitated RNAs resulted from a direct interaction with RNase III. The molecular mechanism of RNase III action on several target RNAs was then studied in more detail both *in vivo* and *in vitro* (Table 1).

### RNase III initiates maturation of rRNA operons

**Figure 2:**
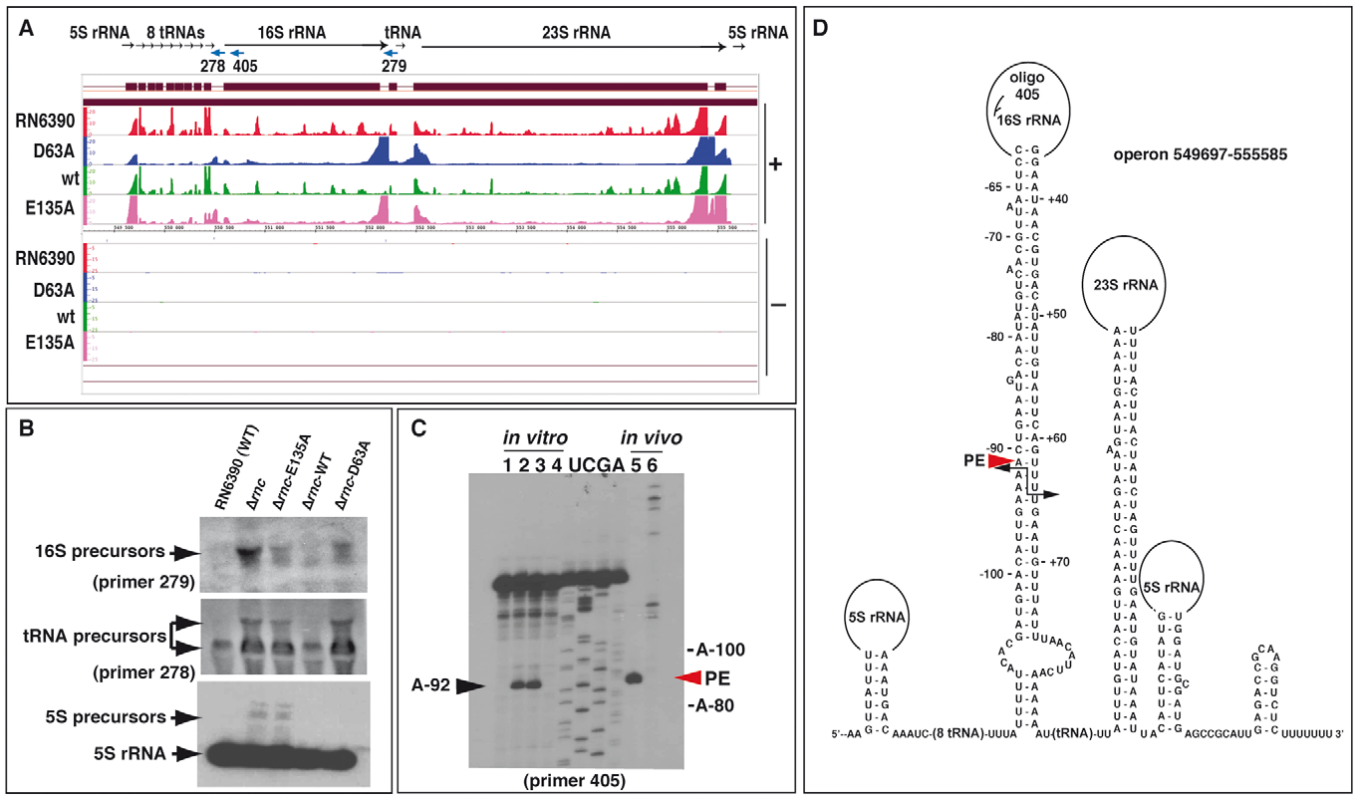
Processing of the rRNA operon by RNase III. (A) Visualization of RNA fragments identified by deep sequencing in the *S. aureus* genome using the Integrated Genome Browser (IGB, Affymetrix). For mapping of cDNAs, the genome sequence of strain N315 was used. Sequenced cDNA reads of RNAs obtained from RN6390 strain (negative control) are shown in red, RNA fragments co-immunoprecipitated with the Flag-RNase III D63A mutant in blue, Flag-RNase III wild type (wt) in green, and Flag-RNase III E135A mutant in magenta. (+) Indicates the leading and (−) the lagging strand, respectively. The Y-axis indicates a relative score for the number of mapped reads per nucleotide normalized to the total number of reads. (B) Analysis of rRNA and tRNA precursors in the WT and mutant Δ*rnc* strains. Northern blot analyses were performed with 5′ end-labeled oligonucleotides using RNAs isolated from RN6390 (WT), the Δ*rnc* strain (Δ*rnc*) or the same strain transformed with a plasmid expressing either the E135A mutant (Δ*rnc*-E135A), the wild type (Δ*rnc*-WT) or the mutant D63A (Δ*rnc*-D63A) enzymes. 5S rRNA was detected with a specific DIG-labeled riboprobe, which was produced by *in vitro* T7 transcription from a PCR product amplified with the oligonucleotides 381/382 (Table S8). Arrows denote the different precursors. (C) Identification of RNase III cleavages *in vitro* and *in vivo* using primer extension. The cleavages were mapped *in vitro* on the 16S rRNA transcript carrying 134 nucleotides at its 5′ trailer region and 100 nucleotides at its 3′ trailer (lanes 1 to 4), and *in vivo* on total RNA extract (lane 5, WT strain; lane 6, Δ*rnc* strain). RNase III cleavage sites were detected by reverse transcription using the 5′ end-labeled oligonucleotide 405. Lane 1: Incubation control; lanes 2–4: *in vitro* cleavage assays performed with RNase III WT on pre-16S rRNA using two concentrations of RNase III (lane 2, 0.1 µM; lanes 3–4, 0.2 µM) in the presence of Mg^2+^ (lanes 1–3) or of Ca^2+^ (lane 4); lanes U, C, G, A: sequencing ladders corresponded to the RNA sequence. Primer extension was performed on total RNAs prepared from exponential cultures of RN6390 (lane 5) and Δ*rnc* strains (lane 6). (D) Secondary structure prediction of the corresponding pre-rRNA operon. Black arrows show positions of the RNase III cleavages, which were experimentally mapped in the 16S pre-rRNA. PE denotes the primer extension stop (red arrow) obtained from total RNA extract.

A high number of reads were mapped to the five rRNA operons and several isolated tRNA operons. The most highly enriched RNA fragments, pulled down with the mutant proteins, corresponded to the intergenic regions of the rRNA operons (Figure 2A). This strongly suggests a role of RNase III in rRNA and tRNA processing as it was previously demonstrated in *E. coli* [34] and *B. subtilis* [22]. We probed one of the five rRNA operons in WT and mutant strains using antisense oligonucleotides complementary to different tRNA and rRNA intergenic sequences (Figure 2B). As expected in the case of impaired rRNA processing, 16S precursor transcripts were observed in the Δ*rnc* strain and in the same strain complemented with either the E135A or D63A mutant enzyme, but not in the RN6390 (WT) strain or in the Δ*rnc* strain complemented with WT RNase III. In addition, aberrant precursors from 5S rRNA and tRNAs were visible on Northern blots probed with a specific DIG-labeled riboprobe or the 5′ end-labeled oligonucleotide 278, respectively, in Δ*rnc* cells and in the same strain expressing the mutant E135A protein (Figure 2B).

Secondary structure analysis of the rRNA operon transcripts predicted that the termini of 16S rRNA and 23S rRNA might each base-pair within long helical domains, generating a typical RNase III substrate (Figure 2D). RNase III cleavage assays were performed on an *in vitro* transcribed 16S rRNA containing the 5′ and 3′ end trailing sequences (see Text S1). Cleavage sites were identified by primer extension on the cleaved rRNA with reverse transcriptase using either the 5′ end-labeled oligonucleotide 405 (Figure 2C) or the 5′ end-labeled oligonucleotide 279 (result not shown). Two specific RNase III cuts were identified at positions A-92 (Figure 2C) and U+64 (result not shown), respectively. These cleavages produced a two-nucleotide 3′ overhang, a hallmark of processing by RNase III (Figure 2D). Primer extension was also performed on total RNA extracted from the WT and Δ*rnc* strains. Using the 5′ end-labeled oligonucleotide 405 that hybridizes within the 16S rRNA, a major reverse transcriptase (RT) stop at A-91 within the 5′ trailer of the 16S rRNA precursor was only seen in the WT strain. Thus, the *in vivo* and *in vitro* RNase III cleavages within the 16S rRNA precursor were congruent (Figure 2C). Interestingly, in the Δ*rnc* strain, several RT stops were detected upstream of A-92 *in vivo* (Figure 2C, lane 6), suggesting that another ribonuclease might target the same region in the absence of RNase III. Such alternative rRNA processing that permits the production of functional ribosomes tentatively explains why the Δ*rnc* mutation in *S. aureus* has only minor effects on cell viability [23], [24].

### RNase III autoregulates its own synthesis at the post-transcriptional level

**Figure 3:**
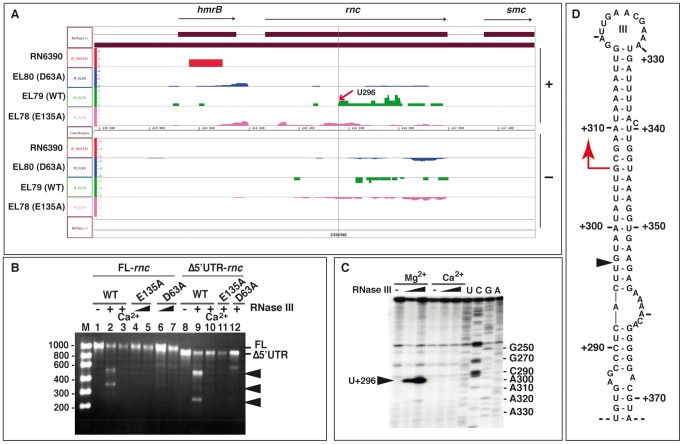
RNase III autoregulates its own expression. (A) Visualization of mapped cDNA reads on the *S. aureus* genome corresponding to *rnc* mRNA fragments using the Integrated Genome Browser (IGB, Affymetrix). Same legend as described in Figure 2A. The red arrow indicates the start of a cleaved fragment at position +U296. (B) *In vitro* RNase III cleavage assays on unlabeled full-length *rnc* mRNA (FL-*rnc* 843 nt, lanes 1–7) or the *rnc* mRNA lacking its 5′ untranslated region (Δ5′UTR-*rnc* 752 nt, lanes 8–12). The RNA fragments were separated using an agarose gel under denaturing conditions and visualized after ethidium bromide staining. Unlabeled *rnc* mRNA (200 nM) was incubated with purified wild type RNase III (WT) (lanes 2, 3, 9, 10) or the mutants E135A (lanes 4, 5, 11) or D63A (lanes 6, 7, 12). Lanes 1, 8: incubation controls of *rnc* mRNAs in the absence of enzyme. Cleavage assays were performed with WT-RNase III at 0.33 µM in a buffer containing Mg^2+^ (lanes 2, 9) or Ca^2+^ (lanes 3, 10), with the mutant E135A at 0.33 µM (lanes 4, 11) and 0.66 µM (lane 5), and with the mutant D63A at 0.33 µM (lane 6, 12) and 0.66 µM (lane 7). Lane M: Riboruler low range RNA marker (Fermentas). (C) RNase III cleavage sites were mapped on *in vitro* transcribed *rnc* mRNA using primer extension with the 5′ end-labeled oligonucleotide 69 (Table S8). Reactions were performed in the presence of increasing concentrations of wild type RNase III (0.33 and 0.66 µM) in a buffer containing either Mg^2+^ or Ca^2+^. Lanes U, C, G, A: DNA sequencing reactions but the labels correspond to the RNA sequence. The black arrow denotes the RNase III cleavage at U+296. (D) Secondary structure of the RNase III-binding site located in the coding region of *rnc* mRNA. A black triangle indicates the *in vitro* cleavage site at position U+296 while the red arrow represents the primer extension stop.

Reads, mapping to *rnc* mRNA, were consistently recovered with both the WT and the two RNase III mutants but not in the control coIP (Figure 3A). These data suggest that RNase III of *S. aureus* specifically recognizes its own mRNA. This hypothesis is supported by the Western blot of the E135A, D63A, and WT proteins expressed in the Δ*rnc* strain because the two mutant proteins accumulated to higher levels than the fully catalytically active WT enzyme (Figure 1D). Prior to mapping the RNase III cleavage site, we determined the 5′ end of the *rnc* mRNA *in vivo* by primer extension (Figure S2A). Two major reverse transcriptase (RT) stops were found, one located at G+306 in the coding sequence and the other >70 nt upstream of the AUG start codon (Figure S2A). Several weaker stops were also observed, e.g. at position U+296, after longer exposure of the autoradiography (Figure S2A). Given the location in the CDS, the RT stop at G+306 represented an internal cleavage of *rnc* mRNA. We then mapped the RNase III cleavage sites on *in vitro* synthesized full-length *rnc* mRNA (843 nt) and a truncated version (752 nt) in which a large part of the 5′UTR had been deleted (Figure 3B). The unlabeled RNAs were subjected to RNase III hydrolysis, and the RNA fragments were separated on agarose gels under denaturing conditions followed by staining with ethidium bromide (for experimental details, see Text S1). RNase III specifically cleaved the *in vitro* transcribed *rnc* mRNA and generated at least two main fragments in a Mg^2+^-dependent manner (Figure 3B). Removal of the 5′UTR altered migration of the smaller fragment, identifying this fragment as 5′ proximal. This result suggests that RNase III recognizes and cleaves its own CDS.

The RNase III cleavage sites were then mapped more precisely by reverse transcription *in vitro* (Figure 3C). This experiment showed that position U+296, located within the CDS of *rnc* mRNA, is the site of the major RNase III-dependent cleavage. Notably, this cleavage coincided with the 5′ end of the RNA fragment recovered by coIP with WT RNase III (Figure 3A). It is surprising, however, that the primer extension performed on total RNA identified a potential RNase III-dependent cleavage at G+306 of *rnc* mRNA, 10 nucleotides downstream of U+296 (Figure 3C and 3D). Although we do not exclude that RNase III cleaves its own mRNA differently *in vivo*, additional trimming of the cleaved RNA by an unknown ribonuclease could tentatively explain this difference. Structure probing of the *rnc* mRNA was performed using the single-strand-specific RNases T2 and T1, and the double-strand-specific RNase V1 (Figure S2B and S2C). Enzymatic reactions were restricted to less than one cut per molecule, and cleavages were mapped by reverse transcription [35]. The structure probing supported the formation of three long hairpins in the CDS, as indicated by numerous RNase V1 cleavages located in the arms and strong RNase T2/T1 cuts occurring in the apical loops (I, II, and III) and the internal loop regions (Figure S2C). The long irregular helix III, in which the RNase III cleavage site at U+296 is located, appears to be the preferred RNase III binding site (Figure 3D).

Taken together, the data support a model wherein RNase III initiates decay of its own mRNA within the CDS, resulting in negative feedback regulation of its expression. The deep sequencing analysis additionally revealed several RNA fragments that were antisense to *rnc* mRNA (Figure 3A). However, expression of these asRNAs was not detectable by Northern blot experiments in the RN6390 strain, indicating a very low abundance and/or low stability of these transcripts.

### The 5′ untranslated region of *cspA* mRNA is processed by RNase III

**Figure 4:**
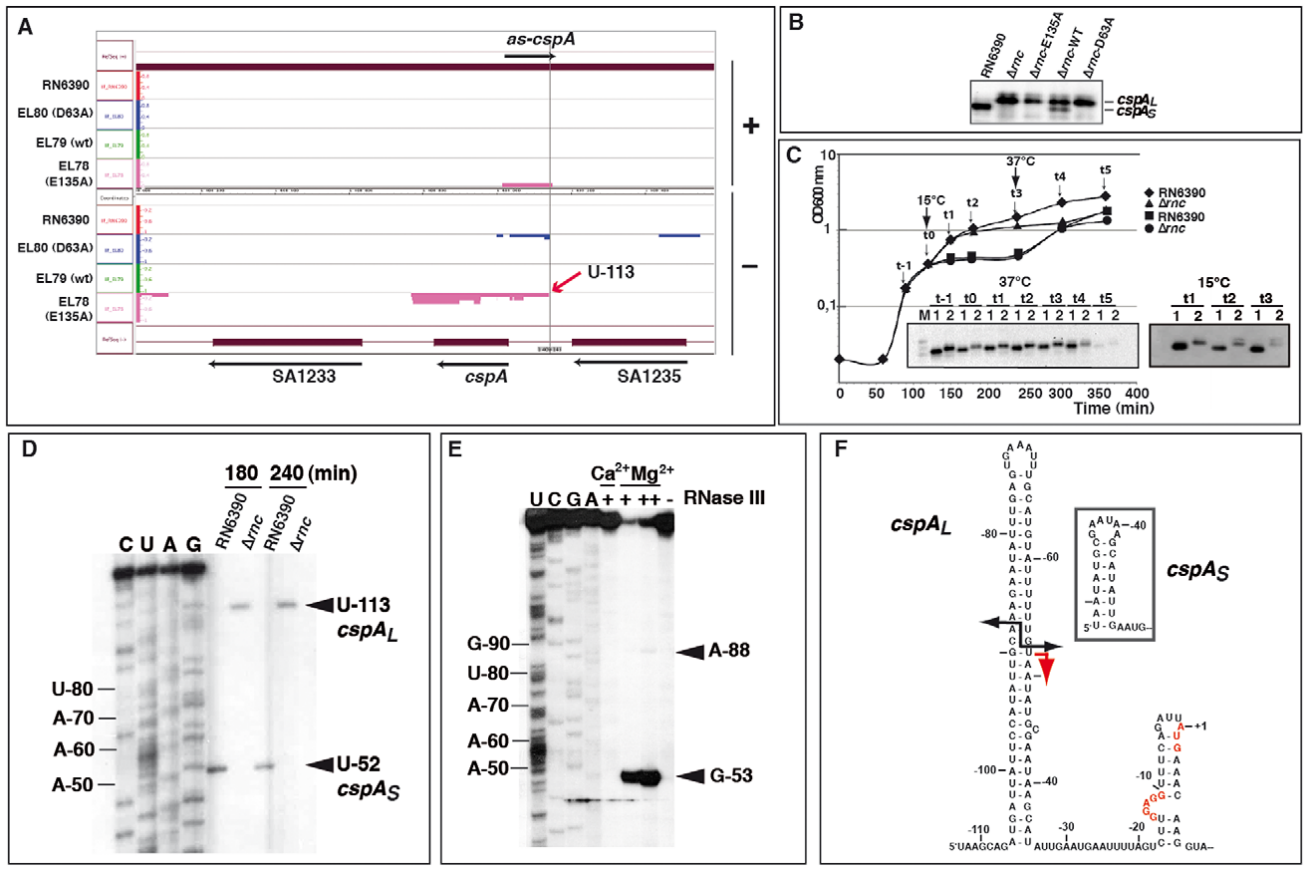
RNase III processes the 5′ untranslated region of *cspA* mRNA. (A) IGB representation of the *cspA* locus. Same legend as in Figure 2A is applied. The +1 site identified by primer extension in the Δ*rnc* strain is indicated by a red arrow (U-113). (B) Expression of *cspA* mRNA forms after 4 h of growth at 37°C in strains RN6390 (WT), Δ*rnc* and Δ*rnc* expressing the E135A, WT or D63A Flag-tagged RNase III. *CspA_L_* corresponds to the longest form of the mRNA while *cspA_S_* corresponds to the processed form of the mRNA. (C) Growth curves of RN6390 (WT) and Δ*rnc* mutant strains (Δ*rnc*). Two cultures were grown at 37°C (black diamonds: RN6390; black triangles: Δ*rnc*) while two other cultures (black squares: RN6390; black circles: Δ*rnc*) were transferred to 15°C (t0) after 120 min of growth at 37°C, then incubated for an additional 120 min at 15°C, and retransferred to 37°C at time t3. In this experiment, a mild growth defect was observed for the Δ*rnc* strain. However, this defect was not reproducible and could be attributed to a higher level of cell aggregation in this strain during the late exponential phase of growth. Northern blot analyses showing the expression of *cspA* mRNA at 37°C or at 15°C at the indicated time-points are depicted in the insets. Lanes 1, 2: RNA samples prepared from RN6390 or Δ*rnc* mutant strains, respectively. Lanes t-1 to t5: incubation times of cell cultures as shown in the growth curves. A DIG-labeled DNA probe (amplified using the oligonucleotides 286 and 16) was used to detect *cspA* mRNA and the autoradiography was revealed after several seconds. (D) Primer extension analysis performed on total RNAs isolated from cells grown at 37°C for 3 h and 4 h. Lane 1: RN6390 strain; lane 2: Δ*rnc* strain. The 5′ end detected in each strain is indicated. The nucleotides are numbered relatively to the AUG start codon. Lanes C, U, A, G: sequencing ladders. Primer extension was carried out using the 5′ end-labeled oligonucleotide 378 (Table S8). (E) RNase III cleavage of unlabeled *cspA_L_* mRNA. The reactions were done in the absence (−) and in the presence of RNase III (+, 0.33 µM; ++ 0.65 µM) in a buffer containing Mg^2+^ or Ca^2+^. Lanes C, U, G, A: sequencing reactions. The same oligonucleotide 378 was used for reverse transcription to analyze the cleavage sites. The RNase III cut at position A-88 appears as a faint band because the enzymatic reaction is too strong. Arrows denote the specific RNase III-induced cleavages at U-53 and A-88 (relatively to the AUG). Lane (−): incubation control in the absence of RNase III. The cleavages were assigned after primer extension using the 5′ end-labeled oligonucleotide 16. (F) Secondary structure of *cspA_L_* is deduced from structure probing experiments. The structure of the 5′UTR of *cspA_S_* is shown in the inset. The grey arrow corresponds to the RNase III cleavage sites obtained *in vitro* while the red arrow represents the reverse transcriptase stop, which was assigned by primer extension in RN6390 (WT). The Shine and Dalgarno (SD) sequence and the AUG strat codon are indicated in red.

**Figure 5:**
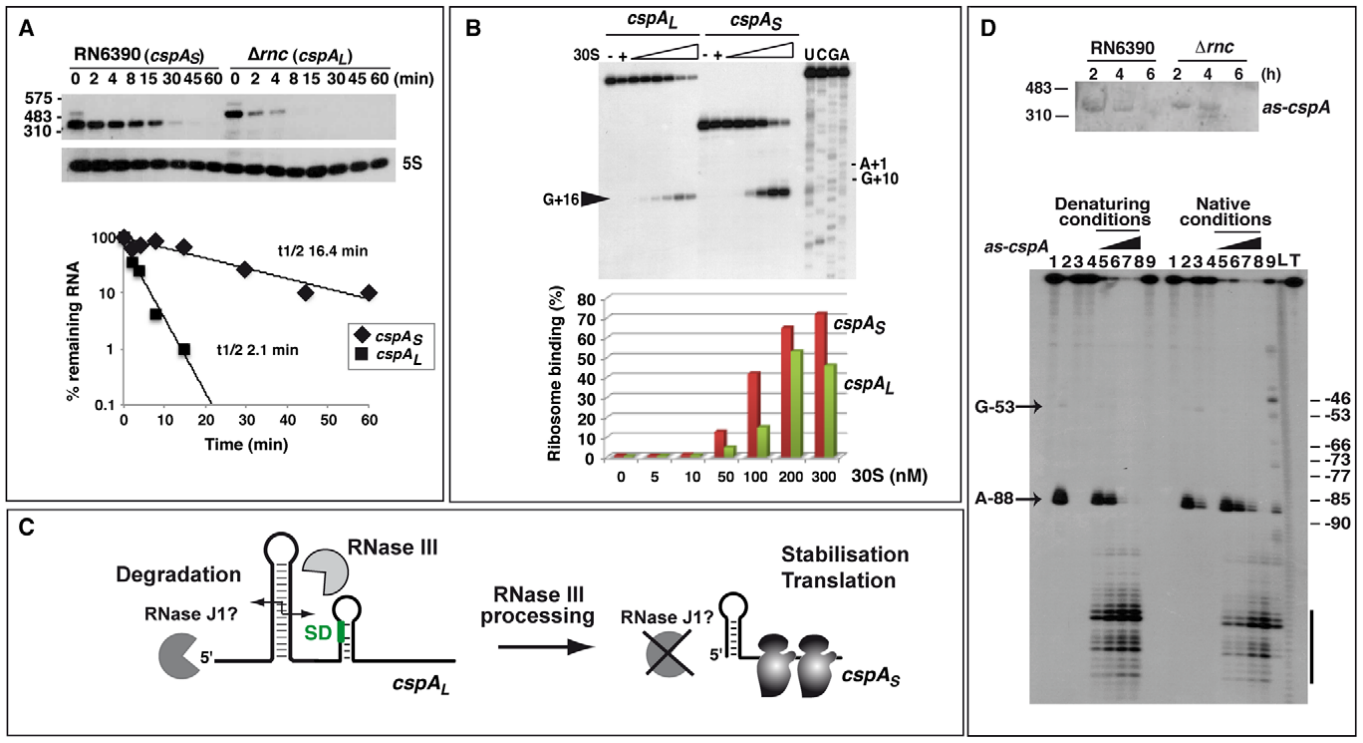
RNase III–dependent processing stabilizes *cspA* mRNA and enhances translation. (A) Upper panel: *cspA* mRNA stability was assessed in RN6390 and Δ*rnc* strains after rifampicin treatment. Expression of 5S rRNA was monitored in the same samples as a loading control. Molecular weight ladders are indicated on the left of the gel. The two forms of *cspA* mRNA were detected using a DIG-labeled riboprobe transcribed by T7 RNA polymerase from a PCR template amplified using the oligonucleotides 367–368 (Table S8). Lower panel: Quantification of *cspA* mRNA level and half-life determination in RN6390 (*cspA_S_*, diamonds) and in Δ*rnc* strain (*cspA_L_*, squares) as a function of time. *CspA_L_* is the unprocessed mRNA and *cspA_S_*, the processed mRNA. The value corresponding to the percentage (%) of the remaining mRNA was normalized with the control experiment performed with 5S rRNA. The half-life was determined from a semi-logarithmic plot of the concentration of the mRNA over time. The slope of the best-fit line was then determined to calculate the half-life, which corresponded to the time-point where 50% of the initial mRNA amount remained. Three experiments provided reproducible results. (B) Formation of the ternary 30S initiation complex using the two forms of *cspA* mRNA (*cspA_L_*, and *cspA_S_*). Ternary complex formation was monitored in the presence of increasing concentrations of *S. aureus* 30S ribosomal subunit (5, 10, 50, 100, 200 and 300 nM), and the initiator tRNA^fMet^ (1 µM). (−): Incubation controls without 30S. Lanes C, G, U, A: sequencing ladders of *cspA_L_* mRNA. The position of the toeprint at +16 and the +1 site corresponding to the AUG codon are indicated. Primer extension was done with the 5′ end-labeled oligonucleotide 16 (Table S8). Lower panel: Quantification of 30S ribosome binding on *cspA_L_* (green) and *cspA_S_* (red) mRNAs. Relative toeprints were calculated by relating the intensity of the band corresponding to the toeprint at +16 to the sum of the intensities of this band and the band corresponding to the full length RNA. (C) Schematic model summarizing the role of RNase III in *cspA* maturation. RNase III cleaves the long hairpin structure at the 5′ end of *cspA_L_* to produce an mRNA with a shorter 5′ untranslated region, which is more stable and translated with a higher efficiency. (D) Top panel: Northern blot analysis showing the expression of the antisense RNA *as-cspA*. Total RNAs were prepared from RN6390 (RN6390, WT) and Δ*rnc* mutant (Δ*rnc*) strains at 2, 4 and 6 h of growth at 37°C. Molecular weight ladders are indicated on the left of the gel. To detect *as-cspA*, a DIG-labeled riboprobe was transcribed *in vitro* with T7 RNA polymerase from a PCR template amplified with the oligonucleotides 286 and 16 (Table S8). The asRNA signal was detected after a long exposure of the autoradiography (30 min). Bottom panel: autoradiography showing the fractionation of RNase III cleavages of 5′ end-labeled *cspA_L_* mRNA alone or in the presence of the antisense RNA (*as-cspA*). Incubation controls of *cspA_L_* mRNA alone or with *as-cspA* in the absence of RNase III are shown respectively in lanes 1 and 4. The RNase III cleavage assays were done in the presence of Mg^2+^ (lanes 2, 5–8) or Ca^2+^ (lanes 3, 9) with *cspA_L_* mRNA alone (lanes 2–3) or with *as-cspA* (lanes 4–9). The *cspA* mRNA-as-*cspA* duplex was formed with denatured RNAs (denaturing conditions) or with RNAs, which were separately renatured (native conditions) (see Text S1). Increasing concentrations of asRNA were used: 10 nM (lane 5), 25 nM (lane 6), 50 nM (lane 7), and 100 nM (lanes 4, 8, 9). Lanes L, T: alkaline ladder and RNase T1 performed on *cspA_L_* mRNA under denaturing conditions, respectively. Lanes 3, 9 (native conditions): the experiments performed in the presence of Ca^2+^ under native conditions show a residual activity of RNase III due to the presence of Mg^2+^, which was used to fold the RNAs prior to complex formation. The arrow indicates the RNase III cleavage at position A-88 occurring in free *cspA_L_* mRNA, and the bar shows the strongest cleavages induced by the *as-cspA* binding.

The *cspA* mRNA, which encodes the major cold-shock protein and RNA chaperone, was a candidate RNase III substrate because the entire transcript was represented by reads from the coIP with the E135A mutant protein (Figure 4A; Table 1 and Table S4). To validate this target, we used Northern blots to first compare *cspA* expression in RN6390 and the Δ*rnc* strain, in the presence or absence of RNase III WT and mutant proteins (Figure 4B). Surprisingly, the absence of RNase III (Δ*rnc* strain) led to the accumulation of a longer *cspA* mRNA (*cspA_L_*) than that observed in the WT strain (Figure 4B). While complementation of the Δ*rnc* strain by functional RNase III partially restored the WT pattern, the two mutant variants did not (Figure 4B). Thus, RNase III appeared to process the *cspA* transcript into a shorter form (*cspA_S_*). Northern blot analysis was then performed on RNA samples collected throughout growth from WT or Δ*rnc* strains at 37°C, after cold-shock at 15°C (at t0, Figure 4C), and after shifting cultures back to 37°C (at t3, Figure 4C). Under all of these conditions, *cspA_L_* mRNA only accumulated in the Δ*rnc* strain, suggesting that the maturation is not regulated by cold-shock but rather is a step in the normal biogenesis of *cspA* mRNA. Next, we performed primer extension on total RNA extracts for a comparative mapping of the 5′ end of *cspA* mRNA in WT and Δ*rnc* strains (Figure 4D). The 5′ end of the processed *cspA_S_* transcript (WT strain) mapped to U-52, while that of the unprocessed *cspA_L_* mRNA (in Δ*rnc*) was found 60 nucleotides upstream, at U-113 (Figure 4D). Importantly, the 5′ end of the *cspA_L_* transcript precisely matched the 5′ boundary of RNA fragments recovered in the coIPs with the two mutant enzymes (Figure 4A). Thus, the comparison of WT and mutant enzymes pinpointed an RNase III-mediated processing event. We then precisely mapped the RNase III cleavage sites on an *in vitro* synthesized unlabeled *cspA_L_* by reverse transcription (Figure 4E), and by using 5′ end-labeled *cspA_L_* (Figure 5D). RNase III hydrolysis of unlabeled *cspA_L_* followed by primer extension, revealed a major cleavage site at G-53 and a minor one at A-88 (Figure 4E), generating the characteristic two-nucleotide 3′ overhang (Figure 4F). The cleavage at position A-88 was also clearly detected with the 5′ end-labeled *cspA_L_* (Figure 5D). Importantly, cleavage at G-53 matched the 5′ termini of *cspA_S_ in vivo* (Figure 4D). Hence, the RNase III cleavage assay *in vitro* faithfully recapitulated a major step of *cspA_L_* mRNA processing *in vivo*.

### Processing of *cspA* mRNA by RNase III activates CspA synthesis

Having confirmed that RNase III processing occurs within the 5′UTR of *cspA* mRNA, we set out to define the functional consequences of this event. The secondary structures of *cspA_L_* and *cspA_S_* were compared using single-strand-specific RNases (RNases T2 and T1) and the double-strand-specific RNase V1 on *in vitro* synthesized mRNAs (Figure S3A). The enzymatic cleavages were mapped by primer extension (for experimental details, see Text S1). The derived secondary structure model supports that *cspA_L_* mRNA is highly structured and starts at the 5′ end with several unpaired nucleotides followed by an almost perfect 32-bp helix (Figure 4F and Figure S3B). This long 5′ hairpin structure resembles a typical RNase III binding site. Shortening of the 5′UTR led to the formation of a smaller but stable 5′ hairpin structure in *cspA_S_* (Figure 4F, inset). Paired nucleotides at the 5′ end of mRNAs are known to protect against pyrophosphate removal by RppH and degradation by the 5′-3′ exoribonuclease activity of RNase J1 in *B. subtilis* [3], [36]. To evaluate the effect of the short stable 5′ hairpin on transcript decay, we analyzed the *in vivo* RNA stability of *cspA* mRNA by Northern blot experiments after rifampicin treatment (Figure 5A). Quantification of the data showed that the processing significantly stabilized *cspA*, increasing transcript half-life from <2.5 min in the Δ*rnc* strain to >16 min in the WT strain (Figure 5A).

We then used toeprinting assays to monitor the formation of translation initiation complexes comprised of *S. aureus* 30S subunits, initiator tRNA^fMet^ and *cspA* mRNA variants (for experiment details, see Text S1). The experiment showed that ∼50% of ternary initiation complexes were formed at 30S concentrations of 120 nM with *cspA_S_* and of 300 nM with *cspA_L_* (Figure 5B). Thus, *cspA_S_* formed initiation complexes more readily than *cspA_L_*. Similarly, a differential proteomic analysis based on two-dimensional gel electrophoresis of cytoplasmic proteins prepared from WT and Δ*rnc* bacteria showed that the synthesis of CspA protein was strongly reduced in the absence of RNase III-mediated processing (data not shown). The increased initiation complex formation of the processed *cspA_S_* mRNA most likely reflects higher accessibility of the RBS as suggested by the enzymatic structure probing of *cspA_S_* mRNA. Indeed, single-strand-specific RNase cleavages were significantly enhanced in the region encompassing the SD sequence in *cspA_S_* (Figure S3). Thus, in the WT strain, the RNase III processing event in the 5′UTR of *cspA_L_* stabilizes the mRNA and facilitates ribosome binding to increase CspA synthesis (Figure 5C). How the long 5′ hairpin of *cspA_L_* hampers ribosome binding remains to be studied. Interestingly, previous work showed that a stable hairpin structure located several nucleotides upstream of a SD sequence sterically interfered with translational initiation [37].

The deep sequencing data indicated the existence of an asRNA complementary to the entire 5′UTR of *cspA_L_* including the six first codons (Figure 4A). Northern blot and primer extension experiments confirmed the presence of this asRNA in both WT and Δ*rnc* strains grown at 37°C (Figure 5D; Table S5). However, the Northern experiments performed with DIG-labeled riboprobes, covering the same region of the genome, suggested that the yield of this asRNA was very low compared to that of *cspA* mRNA (Figure 5D). We tested whether this asRNA guides RNase III cleavage of *cspA*. End-labeled *cspA_L_* mRNA was subjected to RNase III hydrolysis *in vitro*, in the absence or presence of the asRNA (Figure 5D). Two conditions were used to form the asRNA-mRNA complexes: both RNAs were either denatured together and directly hybridized (denaturing conditions), or were denatured and refolded separately before hybridization (native conditions). After RNase III hydrolysis, the labeled RNA fragments were separated on a sequencing gel (Figure 5D, lower panel). The results show that RNase III efficiently cleaved preformed mRNA-asRNA duplexes into short RNA fragments *in vitro*. Therefore, the asRNA suppressed rather than promoted the generation of stable *cspA_S_* mRNA. This regulation could contribute to fine-tuning of mRNA levels *in vivo* [28].

Overall, the RNase III-mediated processing step in the biogenesis of *cspA* mRNA is determined by the intrinsic structural properties of its 5′UTR alone. These data strongly suggest that RNase III cleavage activates the synthesis of the major cold-shock protein CspA at the post-transcriptional level.

### Non-coding RNAs as RNase III targets

In addition to several mRNAs, the abundant housekeeping RNAs, tmRNA, RNase P, 4.5S RNAs, and the transcriptional regulator 6S RNA, were significantly enriched in the coIPs with the mutant proteins (Table S2). These ncRNAs are all processed from precursor transcripts by the concerted action of several endo- and exoribonucleases (e.g., [38], [39]). However, the frequent recovery of such abundant and highly structured RNAs does not strictly imply their maturation by RNase III. For example, although *B. subtilis* 4.5S RNA maturation involves RNase III [39], [40], we did neither observe an altered processing pattern or precursor accumulation in the Δ*rnc* mutant strains in Northern blot experiments (Figure S4A), nor did we detect RNase III-dependent cleavages of 4.5S RNA *in vitro* (results not shown). Likewise, the mature 230 nt product of 6S RNA was recovered by coIP, and its irregular hairpin structure was recognized by the RNase III mutant E135A (Figure S1). Nevertheless, we failed to observe RNase III-dependent processing on Northern blots (Figure S4A) and *in vitro* cleavage assays (results not shown). As an aside, the 6S gene is located downstream of the *aspS*-*hisS* operon, which is controlled by a T-Box motif [41], [42]). Whether 6S RNA expression responds to decreased pools of amino acids or uncharged tRNAs remains to be investigated.

**Figure 6:**
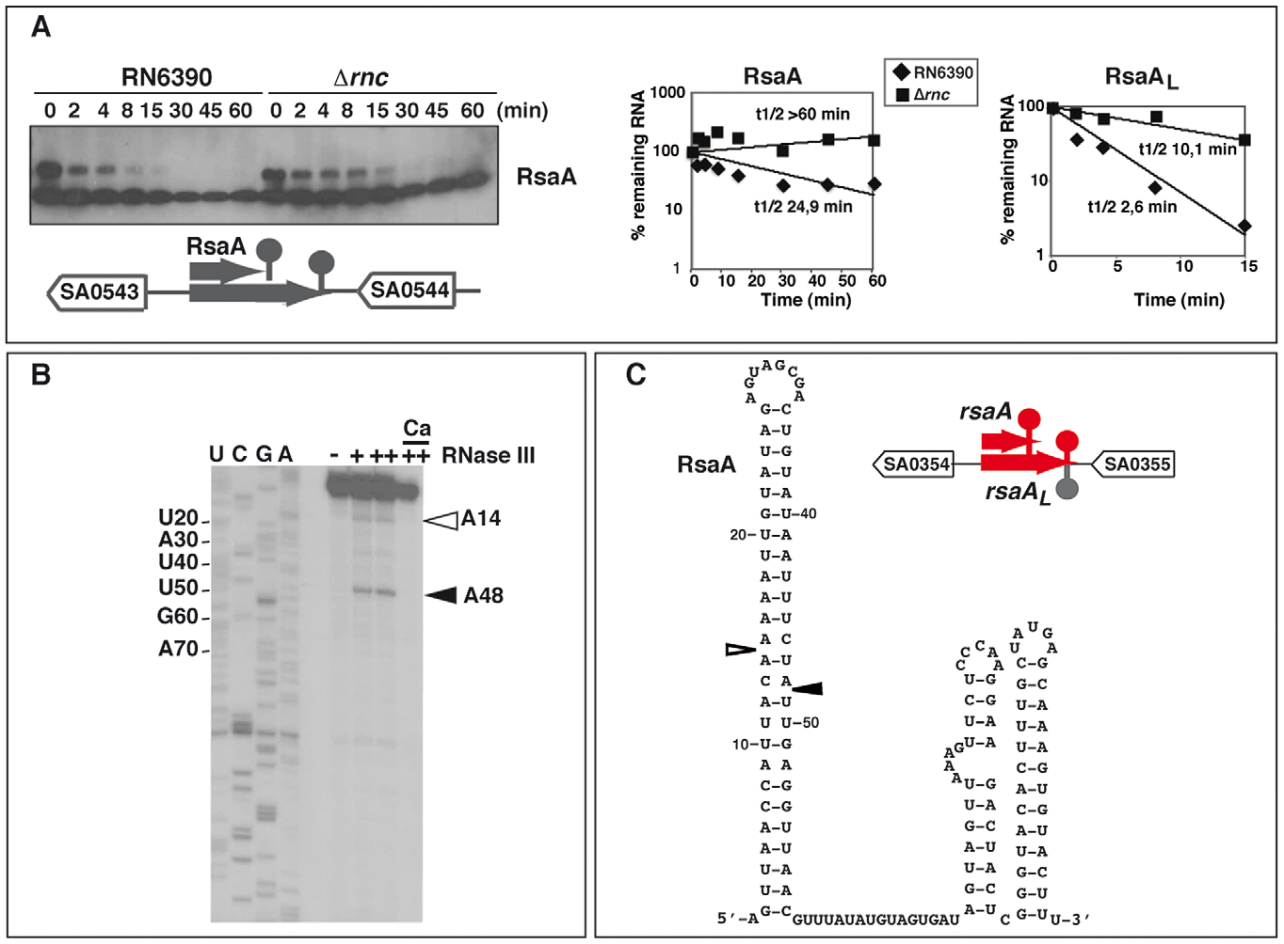
The ncRNA RsaA is a target of RNase III. (A) (Left) The half-life of RsaA was measured as a function of time after rifampicin treatment in the RN6390 and Δ*rnc* strains. A strand specific DIG labeled riboprobe was used to monitor RsaA expression. The oligonucleotides 374 and 376 were used for PCR amplification and the RNA was transcribed *in vitro* with T7 RNA polymerase. (Right) Quantification of mRNA levels in the RN6390 (WT, diamonds) and Δ*rnc* mutant strains (squares) as a function of time after rifampicin treatment. The value of the mRNA half-life was determined by representing a semi-logarithmic plot of the concentration of the mRNA over time. The value corresponding to the percentage (%) of the remaining mRNA was normalized with the control experiment performed with 5S rRNA. The slope of the best-fit line was then determined to calculate the half-life. Three experiments provided reproducible results. (B) RNase III cleavages of cold RsaA *in vitro*. The reactions were done in the absence (−) and in the presence of RNase III (+, 0.33 µM; ++ 0.65 µM) in a buffer containing Mg^2+^ or Ca^2+^. Lanes C, U, G, A: sequencing reactions performed on RsaA_L_. (C) The secondary structure of RsaA was experimentally determined [43]. The two RNase III cleavages are represented as follows: empty and black arrows denote weak and strong cleavages, respectively. The organization of the genes corresponds to the annotation of the N315 genome. The ncRNA genes are shown in red.

Many of the enriched RNA fragments recovered by coIP (listed in Table S3) originated from full-length and *bona fide* ncRNAs of *S. aureus*, such as RsaA, C, E, H, I, and J [43], [44], the pathogenicity island-encoded ncRNAs SprA, SprA3, SprB, SprC, and SprF3/SprG3 [42], [44], as well as RNAIII [45]. Several of these ncRNAs (RsaA, RsaE, RsaX29/X39, RsaI, RsaO, SprA) were enriched with the mutant proteins suggesting that they are substrates of RNase III (Figure S5). These RNAs carry stable stem-loop structures and typical Rho-independent terminator hairpins (Figure 6, Figure S6) [46]. Experimental validation was performed on RsaA (Figure 6). In addition to RsaA, a second larger RNA (RsaA_L_) was detected on Northern blots, which likely originated from read-through at the transcriptional terminator. RsaA and RsaA_L_ share a similar 5′ end as determined by RACE experiments [43]. Half-live measurements revealed a significantly higher RsaA stability in the Δ*rnc* strain (>60 min) compared to WT strain (∼25 min for RsaA; Figure 6A). The longer RsaA_L_ RNA was also more stable in the Δ*rnc* strain (10 min) than in the WT strain (∼2.5 min; Figure 6A). We also performed RNase III cleavage assays on *in vitro* transcribed RsaA followed by primer extension. Two main cleavages were identified in the bulged loop of the 5′ hairpin structure of RsaA (Figure 6B) and of RsaA_L_ (data not shown). Notably, these RNase III-specific cleavages coincided with the 5′ ends of two RNA fragments recovered by coIP with WT RNase III (Figure S5). Thus, RNase III contributes to the turnover of RsaA and RsaA_L_.

This study identified novel ncRNAs such as RsaL, RsaN, and RsaO (Table S3; Figures S4B and S6). Northern blot analyses showed that RsaO was expressed in all strains tested (Figure S4B), while RsaN was only detectable in the Δ*rnc* strain (Table 1 and Table S3, data not shown). Other novel transcripts mapped to loci with multiple copies in the genome. For instance, two of the transcripts with the most abundant sequence reads corresponded to homologous and redundant ncRNAs (RsaX29 and RsaX39) that originated from a partial duplication of the 5S rRNA genes. RsaX29 harbors a long helical structure that might be recognized by RNase III (Figure S6; Table S3).

According to the deep sequencing data, several ncRNAs have associated asRNAs. The abundance of these antisense transcripts varied considerably according to Northern blot experiments (Figure S4C and S4D). For instance, the putative asRNAs of RsaA or RsaH were solely detectable by deep sequencing (results not shown). Conversely, several sense-antisense RNA pairs (SAS028/teg102, SprF3/SprG3) gave strong signals on Northern blots in WT and Δ*rnc* strains (Figure S4C, S4D). Teg102 has been previously identified as an asRNA complementary to SAS028 mRNA, which encodes a small hypothetical protein [44], [47]. Its 5′ half was found in two copies in the same intergenic region of the genome (Figure S4C). The levels of SAS028 mRNA were reproducibly lower in the Δ*rnc* strain overexpressing the WT RNase III (Figure S4C). It remains to be seen whether this RNase III-dependent effect is a consequence of asRNA regulation. SprF3/SprG3, whose partial sequences are present in multiple copies in the genome [42], may belong to the group I toxin-antitoxin systems, with SprG being the putative toxin [48]. Whether SprG3 encodes a peptide is yet unknown. Measurement of the half-lives *in vivo* showed that SprG3 (>60 min) is more stable than SprF3 (<12 min) (Figure S4D). However, under the conditions of growth used in the experiment, the *in vivo* half-lives and the steady-state levels of SprF3 and SprG3 RNAs were similar in the WT and Δ*rnc* strains (Figure S4D) even though RNase III efficiently cleaves the duplex formed *in vitro* (data not shown). These surprising results are reminiscent of a recent study of a *B. subtilis* class I toxin (bsrG)-antitoxin (SR4) system, which showed that the half-lives of bsrG and SR4 RNAs were increased only by 2-fold in a *rnc* mutant [49].

### A possible function of RNase III in the decay of structured regions of mRNAs

**Figure 7:**
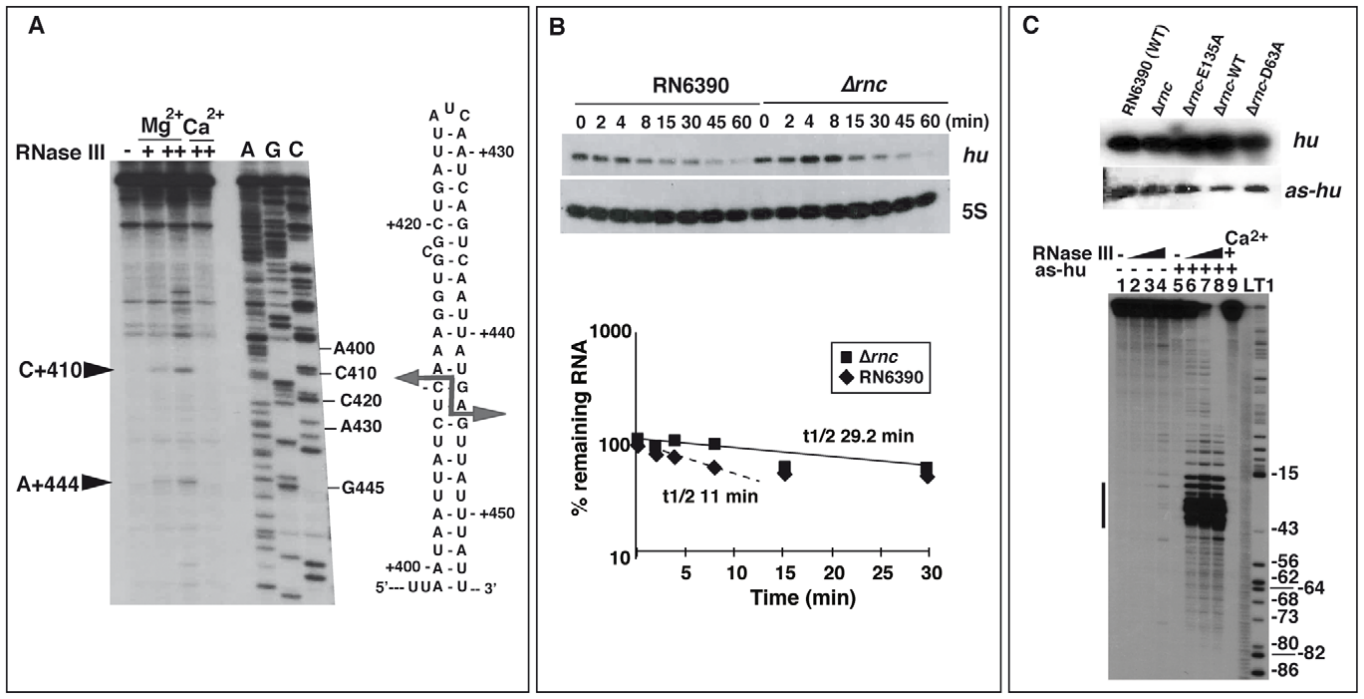
Effect of RNase III on mRNA turnover. (A) RNase III cleavage assays on *in vitro* transcribed *secY* mRNA. The cleavages were assigned after primer extension using 5′ end labeled oligonucleotide 292 (Table S8). RNase III cleavage reactions were done in the absence (−) and in the presence of RNase III (+, 0.33 µM; ++ 0.65 µM) in a buffer containing Mg^2+^ or Ca^2+^. Lanes C, G, A: Sequencing reactions. The arrows denote the RNase III-induced cleavages, which are reported on the secondary structure of the RNase III binding site located in the coding sequence of *secY* mRNA. (B) Analysis of *hu* mRNA expression in RN6390 and the isogenic Δ*rnc* mutant strain. Upper panel: measurements of the half-life of *hu* mRNA by monitoring mRNA levels after rifampicin treatment as a function of time (min). A strand specific labeled riboprobe was used to detect *hu* mRNA. The riboprobe was transcribed *in vitro* with T7 RNA polymerase using a PCR template amplified with the oligonucleotides 370 and 371 (Table S8). As an internal control, 5S RNA expression was detected on the same Northern blot experiment. Lower panel: quantification of *hu* mRNA stability in RN6390 (black diamond) and in Δ*rnc* strain (black square) is given as a function of time. The half-life was calculated as described in Figure 5A. The dotted line represents the half-life for the fraction of *hu* mRNA, which appeared to be degraded in a manner dependent of RNase III. (C) Upper panel: Northern blot analysis of the expression of *hu* mRNA and the antisense RNA (*as-hu*) in various strains: RN6390 (wild type strain), Δ*rnc* mutant strain (Δ*rnc*), and the same strain transformed with plasmid expressing the mutant E135A RNase III (Δ*rnc*-E135A), the wild type RNase III (Δ*rnc*-wt), or the mutant D63A RNase III (Δ*rnc*-D63A). A strand specific riboprobe was used to detect *as-hu* expression. The riboprobe was transcribed *in vitro* with T7 RNA polymerase using a PCR template amplified with oligonucleotides 270 and 71 (Table S8). Lower panel: Autoradiography showing RNase III cleavage products of 5′ end-labeled *hu* mRNA alone or associated with the as-*hu* mRNA. Incubation controls of *hu* mRNA alone or with *hu*-*as* in the absence of RNase III are shown in lanes 1 and 5, respectively. The RNase III cleavage assays were performed in the presence of Mg^2+^ (lanes 2–4 and 6–8) or Ca^2+^ (lane 9) on *hu* mRNA (lanes 2–4) or bound to *hu-as* (lanes 6–9). The *hu* mRNA-as-*hu* duplex was pre-formed with denatured RNAs (denaturing conditions). Increasing concentrations of RNase III were used: 0.165 µM (lanes 2, 6), 0.33 µM (lanes 3, 7) and 0.66 µM (lanes 4, 8, 9). Lanes L, T1: alkaline ladder and RNase T1 ladder of *hu* mRNA, respectively. The bar denotes the shortest *hu* mRNA fragments generated by RNase III cleavage upon the *as-hu* binding.

Deep sequencing of RNase III-associated RNAs recovered several mRNAs that encode proteins of various functions, including regulatory proteins that control the expression of virulence factors (repressor of toxin Rot, transcriptional regulatory protein SarH, two component-system SrrA-SrrB), *bona fide* virulence factors (protein A, the exotoxin Geh), and enzymes involved in various metabolic pathways (Table S4). In many cases, certain mRNA fragments were strongly enriched. This observation might be due to fragmentation occurring during the purification procedure, or alternatively reflect RNase III binding to structured mRNA fragments as a step in promoting their subsequent degradation. Many coIP mRNA fragments contained long hairpin structures which are typical RNase III binding sites, as it is observed for *secY* mRNA (Figure 7A). *In vitro* RNase III cleavage assays were performed on *in vitro* transcribed and unlabeled *secY* mRNA followed by reverse transcription. Two cuts were located in a long hairpin structure within the CDS of one of the coIP fragments, generating typical RNA fragments with a two-nucleotide 3′ overhang (Figure 7A).

Many mRNA fragments corresponded to highly structured 5′UTRs of mRNAs, e.g., *ndrl* and *ptsG* (Figure S7). These 5′UTRs were described as *cis*-acting regulatory elements of downstream genes with functions in the translational machinery or metabolic pathways (Table 1 and Table S4). They contain specific binding sites for diverse ligands such as metabolites, deacetylated tRNAs, or regulatory proteins (ribosomal proteins, antitermination regulatory proteins) [41], [46], [50]. A shared characteristic of most of these structured leaders is the presence of a long Rho-independent terminator structure, indicating that these RNA transcripts resulted from premature transcription termination (Figure S7). Other structured regions in the data sets corresponded to 3′UTRs of mRNAs that all carried stable Rho-independent terminators spanning at least one helical turn, i.e. the minimal substrate of *E. coli* RNase III [51] (Table 1 and Table S4; Figure S7). Several of these 3′UTRs are rather long (>100 nts) and two of them (RsaM, RsaL) correspond to ncRNAs (Table 1 and Table S3) [44], [47].

Overall, these examples illustrate that RNase III might affect the turnover of structured mRNAs, in addition to that of its own transcript and the *cspA* mRNA.

### Identification of numerous antisense RNAs against mRNAs

The coIP strategy using two catalytically impaired RNase III mutant proteins facilitated the identification of asRNAs opposite to 44% of the annotated mRNA genes (Table S5). These asRNAs generally seem to be expressed at a very low level, or are rapidly degraded, since many of them were undetectable on Northern blots (Table 1). One example is *hu* mRNA and its asRNA (Figure 7B, 7C). The stability of *hu* mRNA was measured *in vivo* in WT and Δ*rnc* strains after rifampicin treatment (Figure 7B). Quantification of the data showed that RNase III moderately affected the half-life of *hu* mRNA (Figure 7B). Northern blot experiments performed with DIG-labeled riboprobes, covering identical region of the genome, suggested that the levels of the asRNA were significantly below that of *hu* mRNA (Figure 7C). Moreover, in the Δ*rnc* strain complemented with the WT RNase III, the signal of the asRNA was weaker than in the same strain complemented with the mutant enzymes (Figure 7C). To evaluate whether the asRNA can induce mRNA processing, RNase III cleavage assays were performed on *in vitro* synthesized and 5′ end-labeled full-length *hu* mRNA either free or bound to the asRNA. The cleaved products were resolved on sequencing gels. While the free *hu* mRNA was not efficiently cleaved by RNase III *in vitro* (Figure 7C), the pre-formed asRNA-*hu* mRNA duplexes were strongly cleaved into short RNA fragments (Figure 7C). Thus, *hu* mRNA may be subject to rapid degradation by the combined action of the asRNA and RNase III.

**Figure 8:**
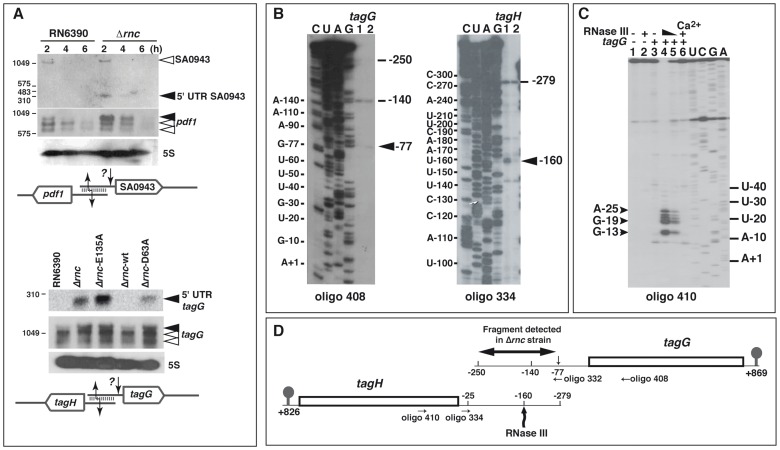
RNase III cleaves mRNAs with overlapping 5′ untranslated regions (UTR). (A) Effect of RNase III on the overlapping 5′ UTRs of SA0943/*pdf1* (top) and *tagG/tagH* (bottom). RNA levels were monitored on Northern blots in various strains: RN6390 (WT), Δ*rnc*, and Δ*rnc* transformed with plasmids expressing E135A RNase III mutant (Δrnc-E135A), the wild type RNase III (Δ*rnc*-wt), or the D63A RNase III mutant (Δ*rnc*-D63A). DIG labeled riboprobes were used to monitor the expression of mRNAs. The riboprobes were transcribed *in vitro* with T7 RNA polymerase using PCR templates amplified with the oligonucleotides 426/427 (SA0943), 429/433 (*pdf1*), 333/334 (5′UTR *tagG*) and 392/393 (*tagG*) (Table S8). Schematic drawings summarizing the data are given below the Northern blot experiments. They show the overlapping 5′UTRs that form a typical RNase III binding substrate. A second cleavage from an unknown RNase (?) generated RNA fragments of 300 nts corresponding to the size of the 5′UTRs of SA0943 or *tagG*, which only accumulated in the Δ*rnc* strain. (B) Primer extension analysis was performed on total RNAs prepared from exponential phase of growth. Oligonucleotides 408 and 334 were used to probe the 5′ ends of *tagG* and *tagH* mRNAs, respectively. Lanes 1, 2: samples prepared from RN6390 and Δ*rnc* mutant strains, respectively. The 5′ ends (−140 in *tagG* mRNA and −25 in *tagH* mRNA) were detected both by primer extension and by RACE experiments performed on circularized RNAs (Table S6). Additional 5′ ends as well as putative RNase cleavage sites are indicated. The nucleotides are numbered relatively to the AUG start codon. Lanes C, U, A, G: sequencing ladders performed on *tagG* and *tagH* mRNAs, which were transcribed from PCR templates amplified with primers 331/221 and 333/384, respectively. (C) RNase III cleavage assays performed with *in vitro* transcribed *tagH* mRNA. Cleavage sites were assigned by primer extension using the 5′ end-labeled oligonucleotide 410. Controls were done with free mRNA (lane 1) or bound to *tagG* mRNA (lane 3); RNase III cleavage assays were performed on the mRNA alone (lane 2) or bound to *tagG* mRNA in a buffer containing Mg^2+^ (lanes 4, 5) or Ca^2+^ (lane 6). Reactions were set with 0.6 µM (lane 2, 5, 6) and 0.8 µM (lane 4) of wild-type RNase III; lanes U, C, G, A: sequencing ladders. (D) Schematic representation of the *tagG-tagH* locus. The RNase III cleavage site in the 5′UTR of *tagH* mRNA at position −160 is shown. An additional cleavage site induced by an unknown RNase is designated by a small arrow at position −77 of *tagG* mRNA. The fragment detected by Northern blot in Δ*rnc* mutant strain (Figure 8A, lower panel) and the hybridization sites of the primers are indicated. The 3′ ends of both mRNAs were identified by RACE on circularized mRNAs (+826 for *tagH* and +869 for *tagG*, respectively).

Several sense-antisense transcript pairs that were strongly enriched by coIP with the mutant proteins corresponded to overlapping UTRs of divergent genes, as illustrated with *pdf1/*SA0943 and *tagG/tagH* mRNAs (Figure 8A; Tables S4 and S5). While *pdf1* encodes the essential peptide deformylase, the *tagG/tagH* genes encode the ABC transporter complex TagGH involved in the export of teichoic acids. Northern blot analysis was performed using specific labeled riboprobes complementary to the 5′UTR of SA0943 or to the CDS of *pdf1* (Figure 8A). In addition to full-length SA0943 mRNA, we observed a weak but reproducible signal for a ∼350 nt long RNA fragment that was only detected in the Δ*rnc* strain (Figure 8A). In contrast, among the three *pdf1* mRNA species, the longest mRNA accumulated strongly in the Δ*rnc* strain (Figure 8A). A very similar pattern was observed for the *tagG/tagH* mRNAs. An RNA probe complementary to *tagG* mRNA detected three forms of the mRNA, the longest of which strongly accumulated in the Δ*rnc* strains expressing the mutant proteins (Figure 8A). Concomitantly, an RNA fragment (<300 nts) corresponding to the 5′UTR of *tagG* was detected in Δ*rnc* cells, suggesting an additional RNase cleavage event. Mapping of the 5′ ends of the *tagG/tagH* mRNAs by primer extension and RACE confirmed that both mRNAs were processed by a mechanism that is partly dependent on RNase III (Figure 8B, Table S6). For *tagG* mRNA, RT stops mapped to positions −140 and −250 in both the RN6390 and Δ*rnc* strains and to position −77 in the Δ*rnc* strain. For *tagH* mRNA, two main RT stops were mapped at −25 and −279 in both strains, while the RT stop at −160 was only observed in the RN6390 WT strain (Figure 8B; Table 1 and Table S6). To assess a functional importance of the observed RNase processing, we further analyzed the RNase III cleavages on *in vitro* transcribed *tagG/tagH* mRNAs containing the long and overlapping 5′UTRs. Using the 5′ end-labeled oligonucleotide 410 complementary to *tagH* mRNA for primer extension, we observed short RNA fragments that were generated by RNase III hydrolysis only when *tagG* associated with *tagH* (Figure 8C). This processing resulted in the formation of a *tagH* mRNA with a shortened leader whose 5′-end lies several nucleotides upstream of the SD sequence (Figure 8D). Thus, RNase III likely targets the 5′ overlapping regions of divergent mRNAs to generate species with shorter or even leaderless 5′UTRs.

## Discussion

Studies of specific transcripts from *S. aureus* have indicated that the regulation of mRNA turnover by RNase III plays an important role in its virulence and adaptation to stress responses [23], [24], [26]. Furthermore, a recent genome-wide analysis revealed an unprecedented high number of asRNAs that are weakly expressed and specifically degraded by RNase III in *S. aureus* and other Gram-positive bacteria [28]. Here to gain better understanding of the broad action of *S. aureus* RNase III, we identified direct target RNAs of this enzyme by deep sequencing of RNAs that were recovered *in vivo* with epitope-tagged variants of the protein. This approach originally pioneered the genome-wide detection of ncRNA and mRNA targets of the RNA chaperone Hfq at single-nucleotide resolution [33], [52]. To facilitate the identification of nuclease targeted RNAs, which normally might be rapidly degraded, we included mutant proteins in which the catalytic activity was uncoupled from their RNA binding capacity (Figure 1A). Based on prior works on *E. coli* RNase III [11], [14], [31], we substituted residues E135 and D63 in *S. aureus* RNase III by alanines. The catalytic activity of the D63A mutant protein was indeed strongly decreased, and the activity of the E135A variant was almost abolished (Figure 1B, 1C). Both mutants retained full RNA binding capacity. Hence, the successful separation of catalytic and RNA binding activity by mutation of the *S. aureus rnc* gene provided independent proof for the contributions of E135 and D63 to the active site of RNase III [10], [14], [31].

Our analysis identified diverse structured transcripts of all gene classes as potential RNase III substrates (Table 1, Tables S2, S3, S4, S5). As expected, the longest RNA fragments were recovered with the two cleavage-impaired mutant enzymes (Table S1), and the highest fraction of mapped cDNA reads corresponded to rRNA and tRNA operons (Figure 2A, Table S2). However, other cDNA reads that mapped to mRNAs including 5′ and 3′UTRs, potential short ORF-containing mRNAs, RNAs from intergenic regions, and asRNAs were specifically enriched in the coIPs with the mutant and WT enzymes (Tables S2, S3, S4, S5). Many of these transcripts carry long hairpin structures, reminiscent of a specific RNase III binding motif (Figures S6 and S7). Thus, RNase III binds many different types of RNAs in the cell and, as discussed below, has a broad effect on RNA processing and turnover. This was recently demonstrated for the *E. coli* and *B. subtilis* RNase III and, interestingly, the steady-state levels of many transcripts showed overlapping effects of *E. coli* RNase III and RNase E [19] and of *B. subtilis* RNase III, RNase J1, and RNase Y [20]. Whether *S. aureus* RNase III acts in a coordinated manner with RNase Y or RNase J1, remains to be studied [53].

### The catalytic activity of RNase III is involved in rRNA processing and *rnc* autoregulation

The requirement for the catalytic activity of RNase III was first demonstrated for the maturation of ribosomal RNA precursors in *E. coli* [34] and *B. subtilis* [22]. Under optimal growth conditions, the synthesis of ribosomes consumes a major fraction of available energy in cells. Thus, maturation of rRNA has to be efficient and accurate for fitness. As in *E. coli* and *B. subtilis* [22], [34], *S. aureus* rRNAs are synthesized as long 30S precursor transcripts containing the three rRNAs genes (16S, 23S, and 5S) interspersed by tRNA genes (Figure 2). Using specific probes that hybridized to the spacer regions of rRNA operons, precursor transcripts were detected in the Δ*rnc* mutant strains (Figure 2B). The identification of RNase III-dependent cleavage in the processing stalk of *S. aureus* 16S rRNA precursors together with the conservation of the precursor structure strongly suggest that the initial processing of rRNAs is carried out by RNase III. Alternative pathways seem to substitute for 16S rRNA maturation in the absence of RNase III (Figure 2C), but the responsible enzymes are not yet known in *S. aureus*. In *B. subtilis*, the final maturation steps of 23S, 16S, and 5S rRNAs involve the mini-III enzyme, the 5′-3′ exoribonuclease RNase J1, and the double-strand-specific RNase M5, respectively [54]–[56]. Because these enzymes are present in *S. aureus*, we may speculate that these maturation pathways are generally conserved in Gram-positive bacteria. Notably, analysis of tRNA/rRNA precursors in the Δ*rnc* strain (Figure 2B) strongly suggested that the maturation of tRNAs is initiated by RNase III cleavage of the large rRNA precursor stalk.

The present study also shows a role of RNase III in gene regulation in *S. aureus*. The enzyme autoregulates its own synthesis by a feedback mechanism similar to that identified in *E. coli* [16], [57] and *Streptomyces coelicolor* [58]. Autoregulation helps to adjust the intracellular amount of the protein to that of the RNA substrates and prevents a potential detrimental over-accumulation of RNase III [59], [60]. We show here that point mutations in the catalytic site of *S. aureus* RNase III cause a two to three-fold increase in the level of the mutant protein compared to the WT enzyme (Figure 1D), which argues that autoregulation depends on the catalytic activity of RNase III. Furthermore, *S. aureus rnc* mRNA is efficiently cleaved by RNase III both *in vitro* and *in vivo* at a specific position in a stem-loop structure located in the CDS (Figure 3D), which is conserved among *Staphylococci*. The ability of RNase III to cleave only one side of the helix is most likely due to the presence of bulged residues that interrupt the helix [27], [61]. We propose that cleavage at this site is responsible for *rnc* mRNA destabilization under conditions when RNase III is in excess over its other RNA substrates. Although the feedback mechanism is preserved in distantly related bacteria, the regulatory site varies. In *E. coli*, RNase III targets a 5′ terminal stem-loop of its own mRNA [57], while the *S. aureus* and *Streptomyces* [58] enzymes regulate themselves via the CDS of their respective gene. Such a structure within the *rnc* coding sequence might locally alter the speed of translation elongation thereby facilitating the access of RNase III.

### RNase III cleavage has a positive effect on protein synthesis

Our results show for the first time that the abundance and translation efficiency of *cspA* mRNA, which encodes the major cold-shock protein CspA, is modulated by RNase III-cleavages within the 5′ leader (Figure 4 and Figure 5). This RNase III processing event generates a more stable mRNA with a shorter 5′ terminal hairpin, which results in strongly enhanced synthesis of the major cold-shock protein. CspA was also found to be involved in the susceptibility of *S. aureus* to an antimicrobial peptide of human cathepsin G thus linking a stress response system to host-pathogen interaction [62]. Interestingly, the 5′UTR of *cspA* is highly conserved in *Staphylococcus* species and *Macrococcus* species, and a similar long hairpin may form upstream of the SD sequence of *cspB* mRNA of *Listeria monocytogenes* (data not shown), suggesting that RNase III-dependent activation may be a conserved mechanism. The fact that RNase J1, a major 5′-3′ exo- and endoribonuclease in Gram-positive bacteria, is inhibited by a 5′ terminal hairpin [53], [63] may explain why the shorter stem-loop structure at the 5′ end stabilizes *cspA* mRNA. In addition, the RNase III-dependent processing of *cspA* mRNA promotes ribosome recruitment, most likely by resolving the inhibitory structure at the RBS.

There are other examples wherein perturbation at the 5′ end impacts the stability and translation of bacterial mRNAs. Binding of deacylated tRNA^Thr^ to the 5′ leader region of *B. subtilis thrS* mRNA induces transcriptional read-through and mRNA cleavage, causing mRNA stabilization due to the formation of a 5′ transcription attenuator hairpin structure [64]. More recently, *Streptococcus pyogenes ska* mRNA is stabilized by the regulatory RNA FasX through the formation of a 9 bp helix at the 5′ end [65]. Similarly, *Clostridium perfringens* collagenase mRNA is stabilized by VR-RNA-dependent cleavage in the 5′ UTR, which renders the SD sequence more accessible for ribosome binding [66]. In contrast with these examples wherein *trans*-acting RNAs are required, we have identified a new mechanism through which RNase III-processing alone confers mRNA stabilization and enhances translation (Figure 5C).

### RNase III is associated with non-coding RNA regulation

We detected 58 ncRNAs that co-immunoprecipitated with RNase III (Table 1, Table S3). Most of these RNAs have been identified previously, and many of them carry hairpin motifs that could be specifically cleaved by RNase III (reviewed in [46]). For instance, RNase III-dependent cleavages were detected in the 5′ hairpin motif of RsaA *in vitro*, and the stability of this RNA was enhanced in the Δ*rnc* strain (Figure 6). Similar to the quorum-sensing-dependent RNAIII, many of these ncRNAs presumably regulate gene expression by antisense mechanisms [27] and it is likely that they would be co-immunoprecipitated with their respective target mRNAs. For instance, RNAIII and two of its major target mRNAs, encoding Rot and protein A, were detected [24], [26], [67]. Likewise, we recovered the 5′UTRs of the *sucC* and *folD* mRNAs, which are known to base-pair with RsaE [43]. Thus, our coIP data sets should be useful to improve the prediction of ncRNA-mRNA interactions. Of note, *E. coli* and *Salmonella* RNase III were also found to affect the steady-state levels of several ncRNAs [19], [68]–[70], suggesting that a significant portion of the *E. coli* transcriptome was directly or indirectly affected by changes in the abundance of the ncRNAs. Thus, RNase III may play a more general role for *trans*-acting ncRNAs than it was previously appreciated.

A significant number of reads representing putative asRNAs complementary to all types of RNA species were found, namely ncRNAs, sORF, and mRNAs (Tables S2, S5). This antisense transcription was directed against 44% of the protein-coding genes. Most asRNAs were present at a low level, suggesting that they might arise from transcriptional noise (e.g., asRNAs against *cspA* and *hu* mRNAs; Figure 5D and Figure 7C). A recent study demonstrated that RNase III might rapidly remove low levels of asRNAs generated by pervasive transcription in *S. aureus* and other Gram-positive bacteria [28]. Interestingly, we observed that *hu* mRNA was more rapidly degraded in the WT strain than in the Δ*rnc* strain (Figure 7B). Because *hu* mRNA was not efficiently cleaved by RNase III (Figure 7C), its rapid degradation might be mediated through asRNA regulation. It is tempting to propose that this RNA quality control mechanism may also contribute to fine-tune the final levels of mRNA in the cell. It is also conceivable that asRNA transcription is transiently enhanced until its concentration reaches a threshold that suffices to regulate the expression of the sense transcript. Indeed, the expression of several asRNAs was recently shown to be SigmaB-dependent, and their decreased expression levels in a Δ*sigB* mutant strain correlated with increasing expression of the sense transcripts [28]. Our data support the view that RNase III-dependent processing indeed contributes to regulate the level of sense mRNA.

Our study further reveals RNase III targets that are derived from long 5′UTRs of divergently transcribed genes. Two of the overlapping 5′UTRs (*tagG/tagH* and *pdf1/*SA0943) are processed by an unknown enzyme to generate mRNAs with shorter 5′ ends, while the processed 5′UTRs are rapidly degraded by RNase III (Figure 8). Shortening of the 5′ end of mRNAs could affect translation and mRNA stability, as illustrated for *cspA* mRNA (Figure 5). A coordinated regulation of TagG and TagH enzymes through overlapping 5′UTRs may be particularly important for the efficient synthesis of teichoic acids in *S. aureus*. Teichoic acids contribute to the structural integrity and shape of the bacteria by regulating the peptidoglycan cross-linking and metabolism during cell division. They are also required for virulence and biofilm formation (reviewed in [71]). Overlapping transcripts from divergently transcribed protein-coding genes with long and overlapping 5′ or 3′UTRs have also been described in *Listeria* [72]. This indicates a mechanism to regulate and coordinate gene expression between neighboring genes.

### Impact of RNase III on gene regulation

In conclusion, this study unveiled the sophistication and complexity of post-transcriptional regulation mediated by RNase III in *S. aureus*. The use of catalytically inactive but binding-competent RNase III mutants allowed the identification of a large set of structured RNase III substrates *in vivo*. For instance, we demonstrated the involvement of the enzyme in rRNA and mRNA processing, in RNA turnover, in the activation of translation through *cis*- and *trans*-acting factors, as well as in antisense RNA-mediated regulation. All of these functions are mediated through the catalytic activity of RNase III. However, we predict that the enzyme may also regulate gene expression through its binding activity, as was shown for the cIII gene of bacteriophage lambda. In this system, RNase III stabilized a conformation of the mRNA that rendered the ribosome binding site accessible to the ribosome [73]. Combining our methodology with comparative proteomics and transcriptomics will help to address more comprehensively the roles of this universally conserved enzyme in gene regulation in response to stress and during host infection.

## Materials and Methods

### Strains and plasmids

Mutations E135A and D63A were introduced into the *S. aureus* RNase III enzyme following the Quickchange XL Site-directed mutagenesis procedure (Stratagene). Experimental details for the preparation of the biological materials and other detailed protocols on Northern blot analysis, RNA structure probing, and toeprinting are given in Text S1. The strains and plasmids used in this study are listed in Table S7.

### Co-immunoprecipitation assays

Wild-type (WT) strain RN6390 or the isogenic Δ*rnc* mutant strains alone or transformed with plasmids expressing either E135A, D63A or WT enzymes were grown in BHI medium at early exponential phase (OD 600 nm 0.2–0.3). Then, 10 µM of CdCl_2_ was added to the cultures, and after 2 h and 4 h of induction, the cells were pelleted and snap-frozen in liquid nitrogen. The bacterial cell pellet was suspended in lysis buffer (TBS, 1% Triton X-100 and protease inhibitor cocktail), transferred onto glass beads (provided by FastRNA Pro Blue Kit, Qbiogene) and processed in the FastPrep instrument (3×45 s at a setting of 6.0). Samples were centrifuged at 13,000 rpm for 5 min. The supernatants were mixed with mouse IgG-agarose (Sigma, A0919) to remove non-specifically binding proteins and incubated at 4°C for 50 min. The beads were spun down (1,500 g, 5 min) and the pre-cleared supernatants (3 ml) were kept separately. A fraction of the volume (0.2 ml) was removed for total RNA isolation and the rest of the sample was mixed with 40 µl (packed gel volume) of Anti-Flag M2 Affinity Gel (Sigma, A2220). Immunoprecipitation was performed according to the manufacturer's instructions. Briefly, the cleared lysates were incubated with the Anti-Flag M2 Affinity Gel for 2 h at 4°C, then the beads were washed three times with TBS. Elution was made with 0.2 ml of Flag Peptide (Sigma, F3290) prepared at the concentration recommended by the supplier. The sample was extracted with acidic phenol and then by chloroform: isoamylic alcohol. RNA was precipitated with ethanol, treated with DNase I, extracted with phenol and precipitated. The final RNA samples were dissolved in 50 µl of sterile water and lyophilized.

### Deep-sequencing analysis

cDNA library construction, pyrosequencing and data analysis were done as previously described [33], [52]. In brief, cDNA-seq libraries were constructed with RNA samples from coIP experiments under exponential and late-exponential phase growth of the Flag-tagged wild-type and mutant enzymes expressed from the inducible plasmid. The resulting cDNA libraries were sequenced on a Roche 454 sequencer using FLX and Titanium chemistry. From the resulting cDNA reads, 5′-linker sequences and polyA-tails were clipped from the sequenced cDNA reads. Only reads of ≥18 nt were aligned to the reference genome, which was retrieved from the NCBI server (accession number of the chromosome: NC_002745.2; accession number of the plasmid: NC_003140.1), using the program *segemehl* [74]. Based on the resulting mapping data, read coverage files were generated in the GR format representing the number of mapped reads per nucleotide. The GR files were visualized in combination with FASTA and GFF files of the genome using the Integrated Genome Browser (IGB) [75]. Additionally, overlaps of mapped reads and gene annotation positions were identified and counted. The overlap between mapped read and a gene annotation had to be at least 10 nucleotides long to be taken into account. Each single overlap counting was normalized by the number of positions to which the overlapping read was mapped and the number of annotations that overlap with the read. For instance, if reads map to multiple regions with exactly the same score (e.g. this is the case for reads that map to the different multiple copies of the rRNA genes), only a relative fraction of one read is counted instead of a count of one read. For example, if a read maps twice, each location gets a score of 0.5 reads. Moreover, if a read overlaps two annotations, each annotation gets a score of 0.5 reads (Table S1).

Text S1 provided experimental details for all the experiments performed in this study.

## Supporting Information

Figure S1 Mutant RNase III E135A binds to the co-immunoprecipitated RNAs *in vitro*. (A) Binding of the mutant E135A RNase III to various RNAs assessed by gel retardation assays. The assays were performed with *in vitro* transcribed unlabeled RNA fragments (50–100 nM), which were incubated with increasing concentrations of E135A enzyme. The complexes were resolved on native agarose gels and subsequently transferred to Hybond-N+ membranes. The free and bound forms of RNAs were revealed after hybridization with a 5′-end labeled oligonucleotide. Data were analyzed using a Phosphoimager (FujiFilm FLA-5100). For the flavin mononucleotide (FMN) riboswitch, the assay was done in the absence (−FMN) or in the presence (+FMN) of the ligand (333 µM). The oligonucleotides used for hybridization are given in Table S8. (B) Binding of the mutant E135A RNase III to the 5′ end-labeled *cspA_L_* mRNA and competition assays. Complex formation was done with the 5′ end-labeled RNA and increasing concentrations of E135A mutant protein (200 to 800 nM). For competition assays, various concentrations of cold competitor RNAs were added. We used *cspA_L_* (10, 50, 100, 200, 500 nM), *cspA_S_* (10, 50, 100, 200, 500 nM), and SA2097 (10, 50, 100, 200 nM). *cspA_S_* is a truncated form of *cspA_L_* and SA2097 is a mRNA which was not co-immunoprecipitated with RNase III. The concentration of E135A mutant protein was 800 nM. (Lane -) no cold RNA was added. The samples were fractionated on 8% (left) and 5% (right) polyacrylamide gel electrophoresis under non denaturing conditions.
(TIF)

Figure S2 Mapping of the 5′ end and secondary structure probing of *rnc* mRNA. (A) Determination of the 5′ end of *rnc* mRNA by primer extension. Total RNA was extracted from different stages of growth of the wild type strain (RN6390). Primer extension was done with 10 µg of RNA. Lanes 1, 4: 240 min of growth; lanes 2, 3: 150 and 180 min of growth, respectively. Two independent experiments were performed with AMV (lane 1) and Superscript (lanes 2–4) RT, respectively. Lanes C, U, A, G: represent DNA sequencing reactions on the full-length *rnc* mRNA transcript, the labels corresponded to the RNA sequence. The 5′ start of the primary transcript is indicated by +1 (approximately 70 nucleotides upstream of the initiation codon AUG). Red arrow corresponds to the RT stop at G+306, the black arrow to the RNase III cut obtained *in vitro* at U+296. Numbering of nucleotides is given relatively to the AUG start codon. A shorter exposition of the autoradiography was performed for a better visualization of the sequencing reactions. For primer extension, the 5′ end-labeled oligonucleotide 380 was used (Table S8). (B) Unlabeled *rnc* mRNA was hydrolyzed in the presence of increasing concentrations of RNase V1 (0.001, 0.002 and 0.01 U), RNase T1 (0.1, 0.2 and 0.4 U) and RNase T2 (0.0125, 0.025 and 0.125 U). Lane (−): incubation control of *rnc* mRNA; lanes A, C, G, U: sequencing reactions. Cuts were detected by primer extension using 5′ end-labeled oligonucleotide 380. (C) Enzymatic cleavages summarized on the secondary structure model of the coding sequence (nts 130–393) of *rnc* mRNA. The grey arrow indicates the RNase III cleavage site at position U+296 obtained *in vitro* and by deep sequencing (Figure 3), and the red arrow corresponds to the reverse transcriptase stop. The annotations of the cleavages induced by RNase T1 (unpaired guanine), RNase T2 (unpaired nucleotides) and RNase V1 (paired nucleotides) are given in the inset.
(TIF)

Figure S3 Analysis of the secondary structure of *cspA* mRNA using enzymatic probing. (A) Enzymatic hydrolysis was performed using *in vitro* transcribed *cspA* mRNAs having a long (*cspA_L_*) or short (*cspA_S_*) 5′UTR. Increasing concentrations of enzymes were added: RNase V1 (0.0001, 0.001 and 0.002 U), RNase T1 (0.1, 0.2 and 0.4 U) and RNase T2 (0.0125, 0.025 and 0.125 U). Lane (−) incubation controls; lanes C, U, G, A are DNA sequencing reactions performed on *cspA_L_* mRNA, the labels corresponded to the RNA sequence. Cuts were detected by primer extension using the 5′ end-labeled oligonucleotide 16 (Table S8). The region of *cspA_S_*, which is more accessible to single-strand specific RNase, is marked by a bar on the right side of the autoradiography. (B) Enzymatic cleavages reported on the secondary structure models of *cspA_L_* mRNA (nts −112 to 257 relatively to AUG). The 5′ end of the processed *cspA_S_* mRNA (red arrow) as well as the labels for the RNase cleavages (grey arrow) are given.
(TIF)

Figure S4 Effect of RNase III on the expression of several ncRNAs and antisense RNAs from *Staphylococcus aureus*. (A) The expression of housekeeping non-coding RNAs (4.5S and 6S RNA) was monitored in various strains: RN6390, the isogenic Δ*rnc* mutant strain (Δ*rnc*), the Δ*rnc* mutant strain transformed with plasmid expressing the mutant E135A RNase III (Δ*rnc*-E135A), the wild type RNase III (Δ*rnc*-wt), and the mutant D63A RNase III (Δ*rnc*-D63A). Grey arrows represent the ncRNA genes. Schematic representation of the genes is according to the N315 genome annotation. (B) Expression of the ncRNA, RsaO, in various strains. Strain annotations are the same as in (A). (C) Expression of SAS028, a mRNA containing a putative small ORF, and its antisense RNA (Sau-02 [17], teg102 [18]). (D) Expression of SprG3 and SprF3 [19] and quantification of RNA stability in RN6390 (diamonds) and Δ*rnc* mutant (squares) strains. Same legend as in B. All the experiments were reproduced at least three times.
(TIF)

Figure S5 Examples of the distribution of cDNA reads represented with the Integrated Genome Browser. Genomic annotation is given at the top of each profile panel. The ncRNA genes are shown by black arrows. (+) and (−) indicate leading and lagging strand, respectively. CoIP RNA was from RN6390 parental strain, and from the mutant Δ*rnc* strain transformed with plasmid expressing wt RNase III, the mutant enzymes RNase III-D63A and RNase III-E135A. E is for exponential phase (4 h) of growth and LE for late-exponential phase (6 h) of growth. Red arrows denote the 5′ end of RsaA RNA fragments that were co-immunoprecipitated with the WT enzyme and which corresponded to RNase III cuts identified by cleavage assays *in vitro*.
(TIF)

Figure S6 Examples of secondary structure motifs as found in several intergenic regions. The genomic organization is depicted and red arrows represent ncRNA genes. Examples of secondary structure motifs found in several ncRNAs as predicted by contrafold [20] and RNAFold [21].
(TIF)

Figure S7 Secondary structures of mRNA fragments co-immunoprecipitated with RNase III. The RNA fragment of *srrA-srrB* mRNA co-immunoprecipitated with the mutant enzymes corresponded to the translational coupling site. The stop codon of *srrA* is depicted in green, the start codon, and the Shine and Dalgarno sequence (SD) of *srrB* are given in red. UTR stands for untranslated region. The secondary structure models were predicted using contrafold [20] and RNAFold [21].
(TIF)

Table S1 Read numbers and Mapping statistics. NC_002745 = *S. aureus* N315 genome; NC_003140 = *S. aureus* N315 plasmid. Immunoprecipitation experiments were carried out in RN6390 strain (wild-type and referent strain) as a control, and in the mutant Δ*rnc* strain transformed with a plasmid expressing either the WT flag-tagged RNase III (IP_EL79), the mutant D63A flag-tagged RNase III (IP_EL80) or the mutant E135A flag-tagged RNase III (IP_E78). Total RNAs were prepared from cells grown at the exponential phase (4 h) and late exponential phase (6 h). * Other stable ncRNAs referred to tmRNA, 4.5S RNA, 6S RNA and RNase P.
(DOCX)

Table S2 List of reads corresponding to rRNA and tRNA operons and to their antisense RNAs. Co-immunoprecipitation (coIP) was done with flag tagged E135A (strain EL78), WT (strain EL79) and D63A (strain EL80) RNase III. In yellow: control experiment was carried out with the untagged WT protein (RN6390). Total RNAs were prepared from cultures grown at the exponential (4 h, Exp) and late exponential (6 h, Late Exp) phase. The overlap between a mapping location and a gene annotation was at least 10 nucleotides long. Each single overlap counting was normalized by the number of mappings of the overlapping read and the number of overlaps of the mapping.
(XLS)

Table S3 Reads corresponding to small non coding RNAs (sRNAs) and their antisense RNAs (asRNA). Co-immunoprecipitation (coIP) was done flag tagged E135A (EL78), WT (EL79) and D63A (EL80) RNase III. In yellow: control experiment was carried out with the untagged WT protein (RN6390).Total RNAs were extracted from cultures grown at exponential (4 h, Exp) and late exponential (6 h, Late Exp) phases. The overlap between a mapping location and a gene annotation was at least 10 nucleotides long. Each single overlap counting was normalized by the number of mappings of the overlapping read and the number of overlaps of the mapping.
(XLS)

Table S4 Reads corresponding to mRNAs. Co-immunoprecipitation (coIP) was done with flag tagged E135A (EL78), WT (EL79) and D63A (EL80) RNase III. In yellow: control experiment was carried out with untagged RNase III (RN6390). Total RNAs were prepared from exponential (4 h, Exp) and late exponential (6 h, Late Exp) phase of growth. CDS is for coding sequence and UTR for untranslated regions of mRNAs. In purple: reads that were not enriched in the coIP with the WT and mutant proteins. They were not considered in the present study. The overlap between a mapping location and a gene annotation was at least 10 nucleotides long. Each single overlap counting was normalized by the number of mappings of the overlapping read and the number of overlaps of the mapping.
(XLS)

Table S5 Reads corresponding to antisense RNAs complementary to mRNAs. Co-immunoprecipitation (coIP) was done with flag tagged E135A (EL78), WT (EL79) and D63A (EL80) RNase III. In yellow: control experiment was carried out with untagged RNase III (RN6390). Total RNAs were prepared from cultures grown at the exponential (4 h, Exp) and late exponential (6 h, Late Exp) phase. The overlap between a mapping location and a gene annotation was at least 10 nucleotides long. Each single overlap counting was normalized by the number of mappings of the overlapping read and the number of overlaps of the mapping.
(XLS)

Table S6 Transcriptional start sites (TSS) of several RNAs that were co-immunoprecipitated with RNase III. (a) Numbering according to N315 genome. (b) Start site as defined by primer extension (PE) analysis using the indicated primer (+1 site detected in wt and Δ*rnc* strains was identical unless otherwise indicated). (c, d) Start site and size of RNAs as defined by deep sequencing data, respectively. Only the longest RNA fragment pulled down by either of the two mutant proteins was indicated. (d) Size of as-*cspA* according to the fragment pulled down with RNase III from samples prepared at the exponential phase of growth (EP); a small fragment was also detected starting at 1408841 from samples prepared at the late exponential phase of growth (SP). (e) References are given for the non coding RNAs for which the exact ends were not mapped in previous studies. Where more than one +1 sites were detected, the main site is indicated in bold letters. In the case of *tagG* and *tagH* mRNAs, the main +1 site was detected by 5′-3′ RACE but could correspond to processed RNA; in the case of *rnc* the main +1 start site is estimated based on the size of the sequenced fragment). nd: not detected.
(DOCX)

Table S7 Strains and plasmids used in this study.
(DOCX)

Table S8 Oligonucleotides used in this study. (a) With bold letters the mutated nucleotides are indicated; (b) With italics the enzyme restriction sites are indicated; (c) With small letters the sequence hybridizing to pQE30 vector is indicated; (d) With small bold letters the Flag-tag is indicated; (e) Underlined is the T7 promoter sequence. Cand is for candidate RNAs: Cand1 is for SprFG3; Cand3: RsaX28; Cand4: RsaN; Cand5a/b: asSAS028 (SAU-02); Cand6: RsaX31; Cand7: RsaX41; Cand8: RsaL; Cand9: RsaM; for additional details see Table S1.
(DOCX)

Text S1 Supplementary Material and Methods.
(DOCX)
