# Supplementary figures and images for: Global Regulatory Functions of the Staphylococcus aureus Endoribonuclease III in Gene Expression
Source: PLoS Genet. 2012 Jun 28;8(6):e1002782. doi: 10.1371/journal.pgen.1002782 (PMC3386247; doi:10.1371/journal.pgen.1002782)

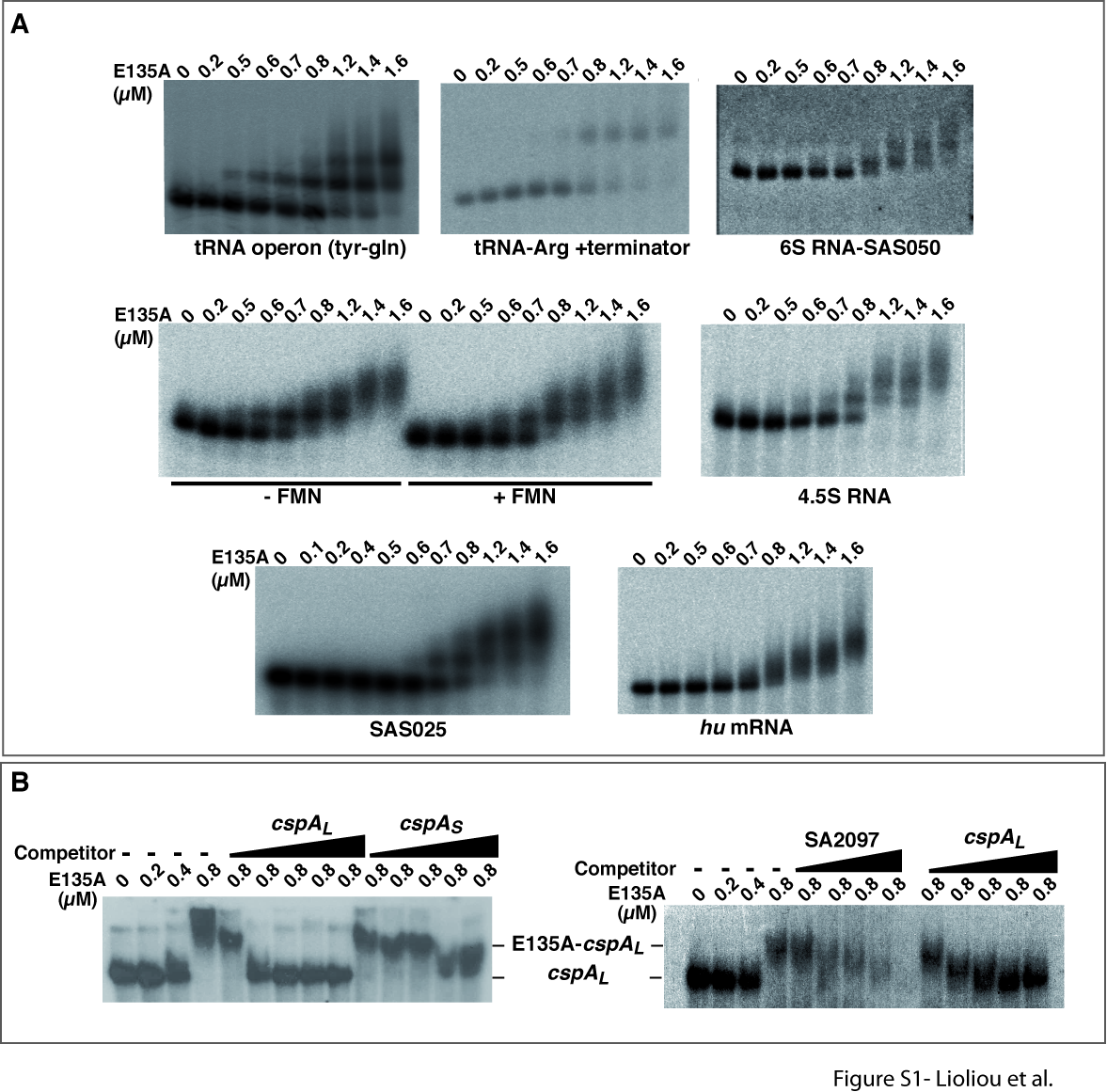

Supplement: Figure S1 — Mutant RNase III E135A binds to the co-immunoprecipitated RNAs in vitro. (A) Binding of the mutant E135A RNase III to various RNAs assessed by gel retardation assays. The assays were performed with in vitro transcribed unlabeled RNA fragments (50–100 nM), which were incubated with increasing concentrations of E135A enzyme. The complexes were resolved on native agarose gels and subsequently transferred to Hybond-N+ membranes. The free and bound forms of RNAs were revealed after hybridization with a 5′-end labeled oligonucleotide. Data were analyzed using a Phosphoimager (FujiFilm FLA-5100). For the flavin mononucleotide (FMN) riboswitch, the assay was done in the absence (−FMN) or in the presence (+FMN) of the ligand (333 µM). The oligonucleotides used for hybridization are given in Table S8. (B) Binding of the mutant E135A RNase III to the 5′ end-labeled cspAL mRNA and competition assays. Complex formation was done with the 5′ end-labeled RNA and increasing concentrations of E135A mutant protein (200 to 800 nM). For competition assays, various concentrations of cold competitor RNAs were added. We used cspAL (10, 50, 100, 200, 500 nM), cspAS (10, 50, 100, 200, 500 nM), and SA2097 (10, 50, 100, 200 nM). cspAS is a truncated form of cspAL and SA2097 is a mRNA which was not co-immunoprecipitated with RNase III. The concentration of E135A mutant protein was 800 nM. (Lane -) no cold RNA was added. The samples were fractionated on 8% (left) and 5% (right) polyacrylamide gel electrophoresis under non denaturing conditions. (TIF) [file pgen.1002782.s001.tif]

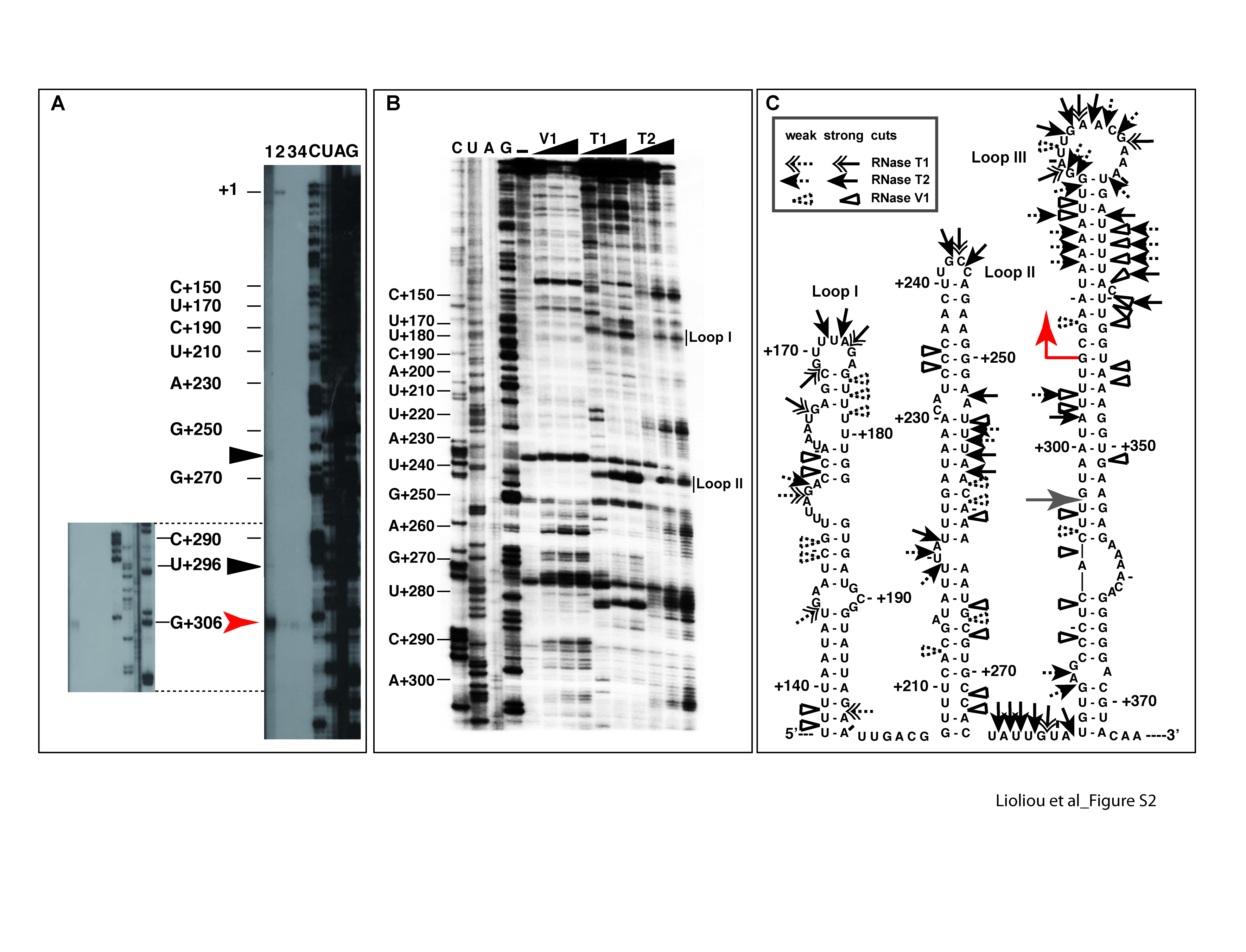

Supplement: Figure S2 — Mapping of the 5′ end and secondary structure probing of rnc mRNA. (A) Determination of the 5′ end of rnc mRNA by primer extension. Total RNA was extracted from different stages of growth of the wild type strain (RN6390). Primer extension was done with 10 µg of RNA. Lanes 1, 4: 240 min of growth; lanes 2, 3: 150 and 180 min of growth, respectively. Two independent experiments were performed with AMV (lane 1) and Superscript (lanes 2–4) RT, respectively. Lanes C, U, A, G: represent DNA sequencing reactions on the full-length rnc mRNA transcript, the labels corresponded to the RNA sequence. The 5′ start of the primary transcript is indicated by +1 (approximately 70 nucleotides upstream of the initiation codon AUG). Red arrow corresponds to the RT stop at G+306, the black arrow to the RNase III cut obtained in vitro at U+296. Numbering of nucleotides is given relatively to the AUG start codon. A shorter exposition of the autoradiography was performed for a better visualization of the sequencing reactions. For primer extension, the 5′ end-labeled oligonucleotide 380 was used (Table S8). (B) Unlabeled rnc mRNA was hydrolyzed in the presence of increasing concentrations of RNase V1 (0.001, 0.002 and 0.01 U), RNase T1 (0.1, 0.2 and 0.4 U) and RNase T2 (0.0125, 0.025 and 0.125 U). Lane (−): incubation control of rnc mRNA; lanes A, C, G, U: sequencing reactions. Cuts were detected by primer extension using 5′ end-labeled oligonucleotide 380. (C) Enzymatic cleavages summarized on the secondary structure model of the coding sequence (nts 130–393) of rnc mRNA. The grey arrow indicates the RNase III cleavage site at position U+296 obtained in vitro and by deep sequencing (Figure 3), and the red arrow corresponds to the reverse transcriptase stop. The annotations of the cleavages induced by RNase T1 (unpaired guanine), RNase T2 (unpaired nucleotides) and RNase V1 (paired nucleotides) are given in the inset. (TIF) [file pgen.1002782.s002.tif]

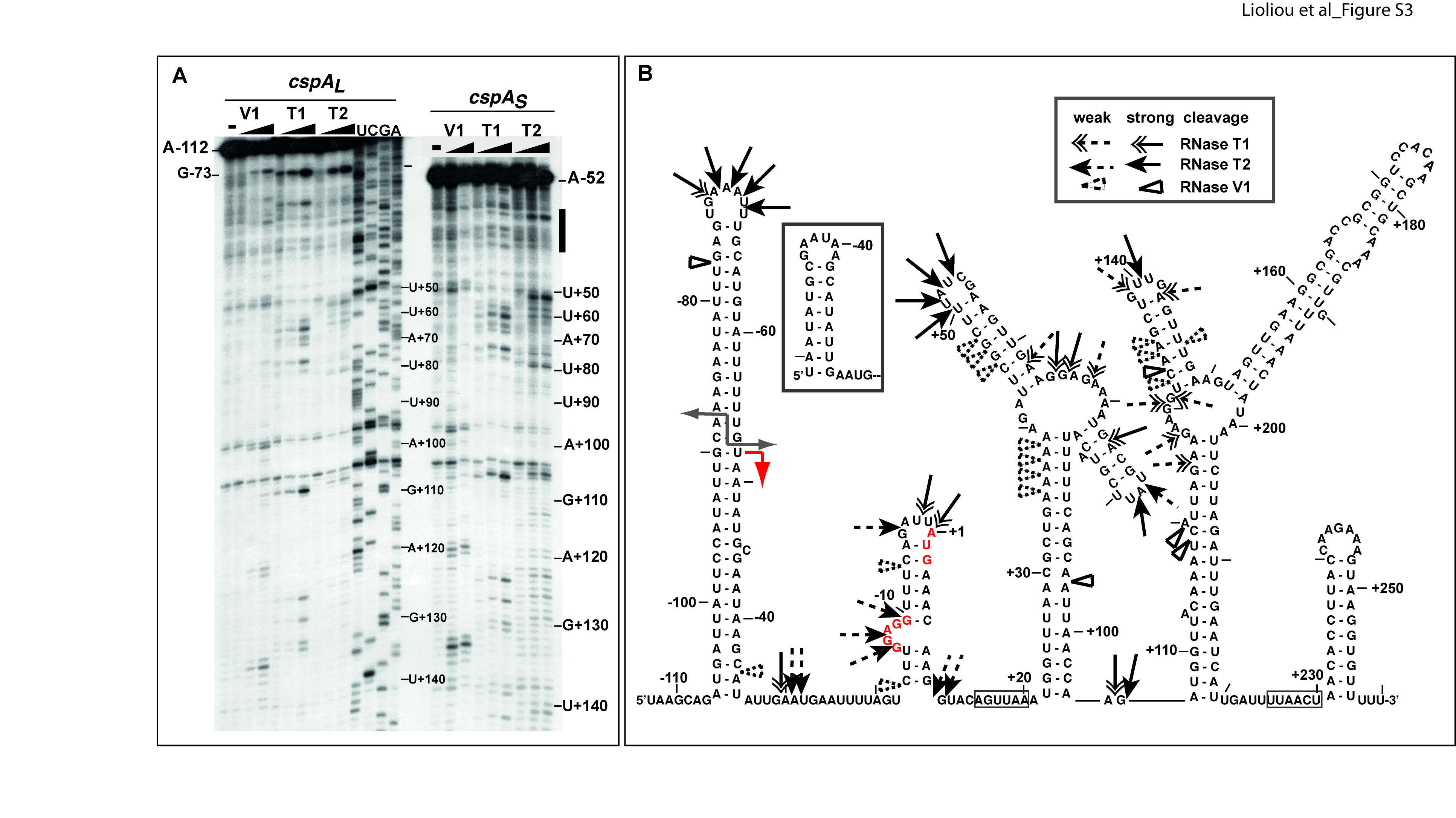

Supplement: Figure S3 — Analysis of the secondary structure of cspA mRNA using enzymatic probing. (A) Enzymatic hydrolysis was performed using in vitro transcribed cspA mRNAs having a long (cspAL) or short (cspAS) 5′UTR. Increasing concentrations of enzymes were added: RNase V1 (0.0001, 0.001 and 0.002 U), RNase T1 (0.1, 0.2 and 0.4 U) and RNase T2 (0.0125, 0.025 and 0.125 U). Lane (−) incubation controls; lanes C, U, G, A are DNA sequencing reactions performed on cspAL mRNA, the labels corresponded to the RNA sequence. Cuts were detected by primer extension using the 5′ end-labeled oligonucleotide 16 (Table S8). The region of cspAS, which is more accessible to single-strand specific RNase, is marked by a bar on the right side of the autoradiography. (B) Enzymatic cleavages reported on the secondary structure models of cspAL mRNA (nts −112 to 257 relatively to AUG). The 5′ end of the processed cspAS mRNA (red arrow) as well as the labels for the RNase cleavages (grey arrow) are given. (TIF) [file pgen.1002782.s003.tif]

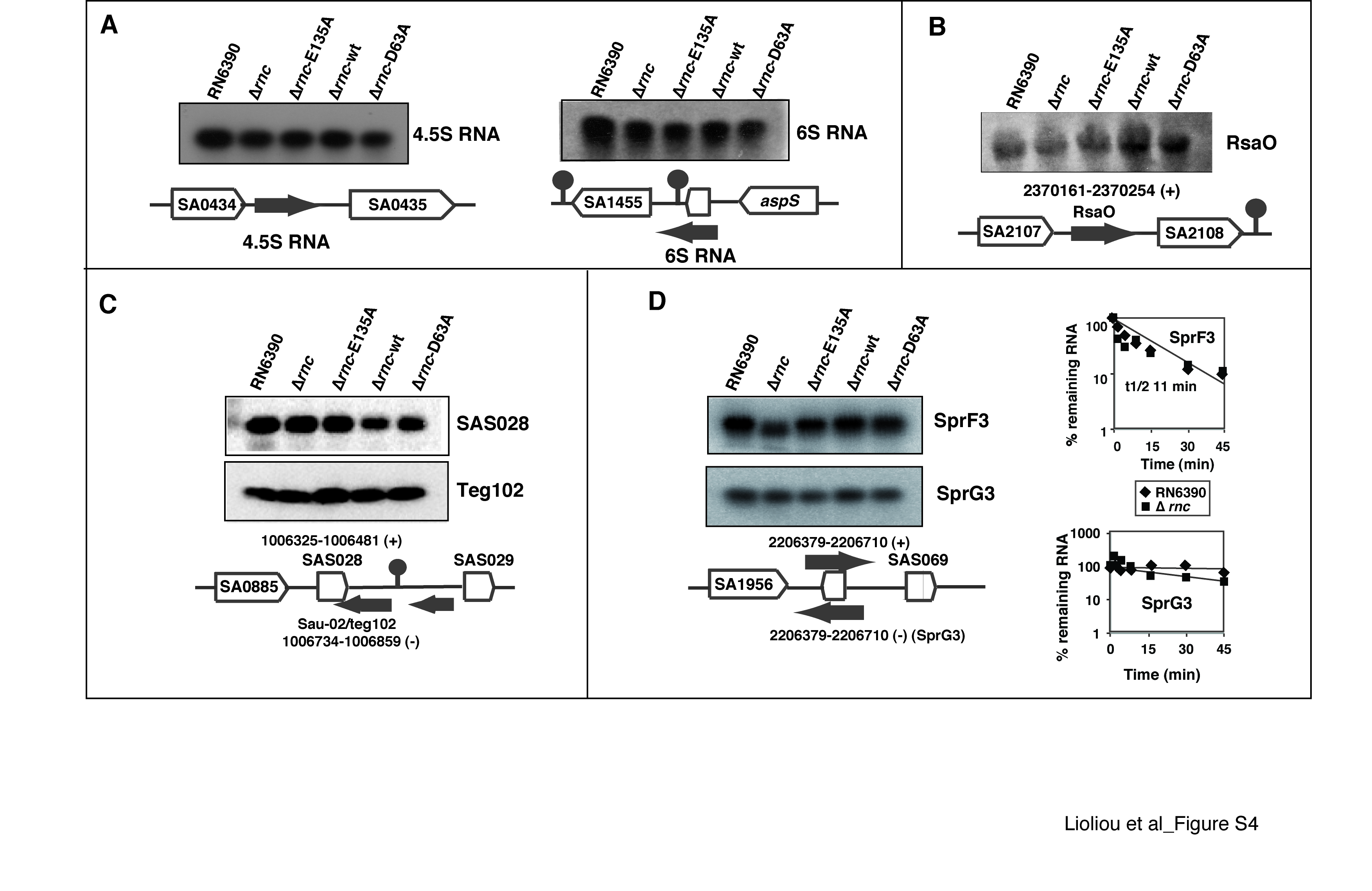

Supplement: Figure S4 — Effect of RNase III on the expression of several ncRNAs and antisense RNAs from Staphylococcus aureus. (A) The expression of housekeeping non-coding RNAs (4.5S and 6S RNA) was monitored in various strains: RN6390, the isogenic Δrnc mutant strain (Δrnc), the Δrnc mutant strain transformed with plasmid expressing the mutant E135A RNase III (Δrnc-E135A), the wild type RNase III (Δrnc-wt), and the mutant D63A RNase III (Δrnc-D63A). Grey arrows represent the ncRNA genes. Schematic representation of the genes is according to the N315 genome annotation. (B) Expression of the ncRNA, RsaO, in various strains. Strain annotations are the same as in (A). (C) Expression of SAS028, a mRNA containing a putative small ORF, and its antisense RNA (Sau-02 [17], teg102 [18]). (D) Expression of SprG3 and SprF3 [19] and quantification of RNA stability in RN6390 (diamonds) and Δrnc mutant (squares) strains. Same legend as in B. All the experiments were reproduced at least three times. (TIF) [file pgen.1002782.s004.tif]

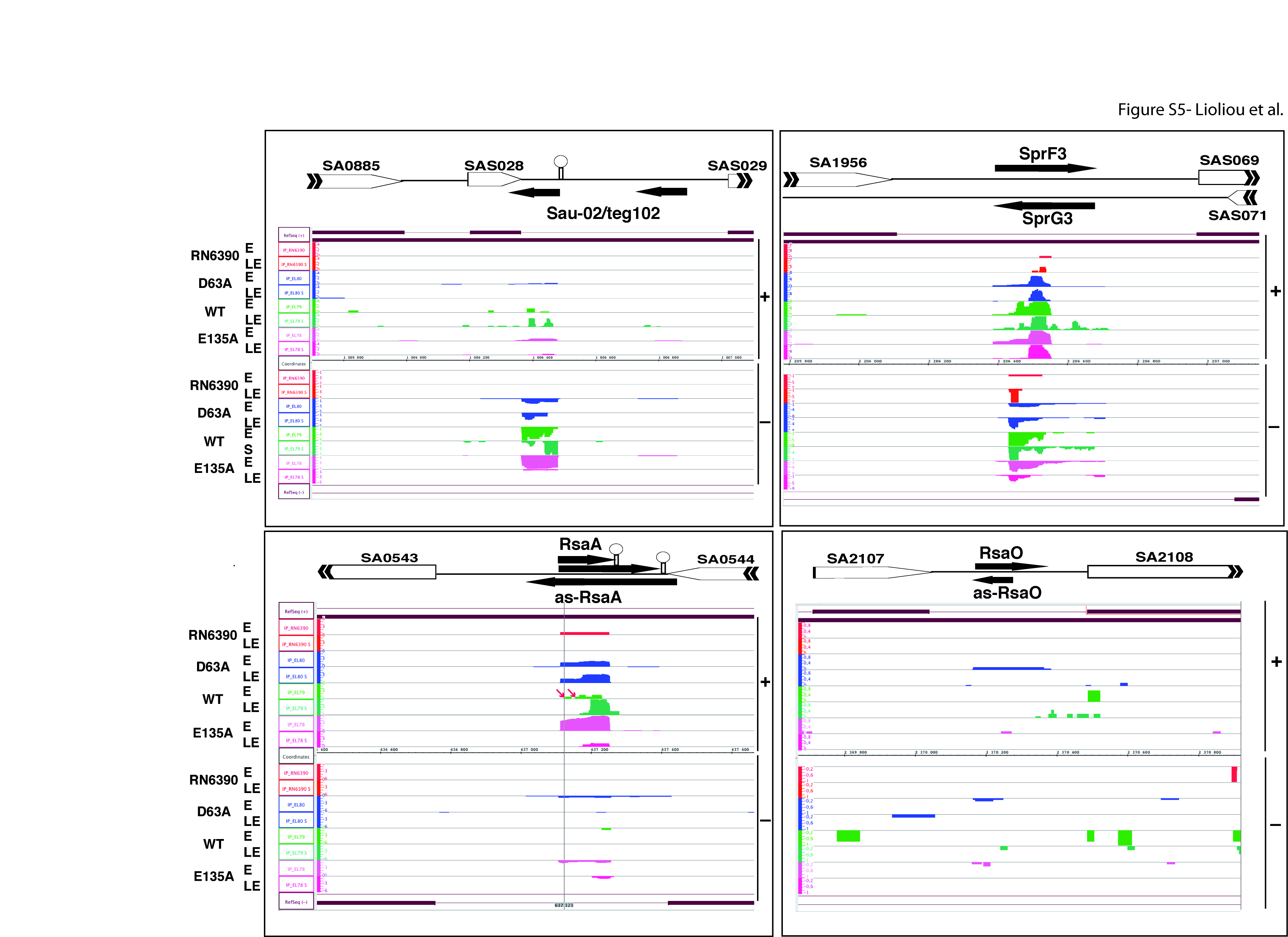

Supplement: Figure S5 — Examples of the distribution of cDNA reads represented with the Integrated Genome Browser. Genomic annotation is given at the top of each profile panel. The ncRNA genes are shown by black arrows. (+) and (−) indicate leading and lagging strand, respectively. CoIP RNA was from RN6390 parental strain, and from the mutant Δrnc strain transformed with plasmid expressing wt RNase III, the mutant enzymes RNase III-D63A and RNase III-E135A. E is for exponential phase (4 h) of growth and LE for late-exponential phase (6 h) of growth. Red arrows denote the 5′ end of RsaA RNA fragments that were co-immunoprecipitated with the WT enzyme and which corresponded to RNase III cuts identified by cleavage assays in vitro. (TIF) [file pgen.1002782.s005.tif]

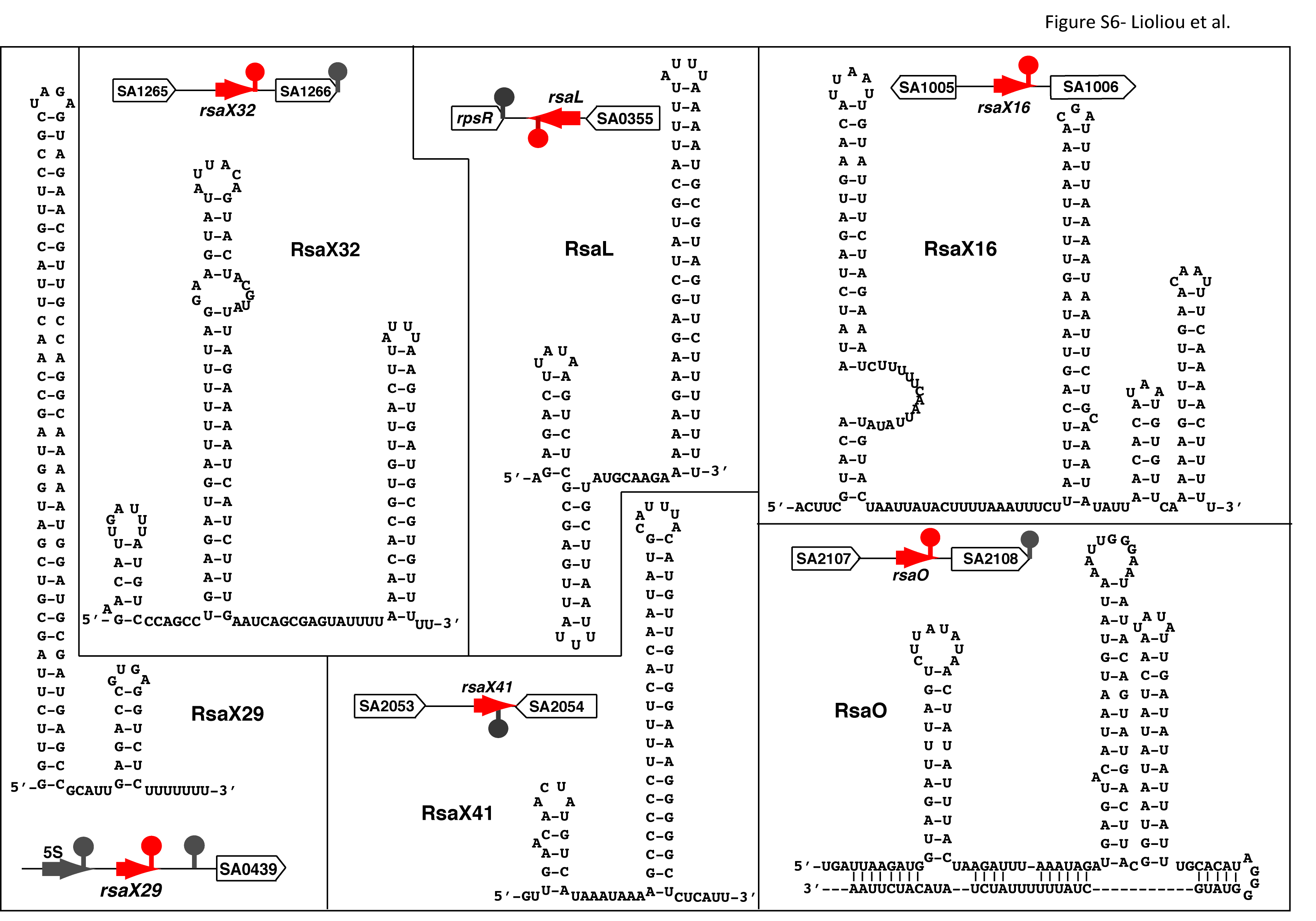

Supplement: Figure S6 — Examples of secondary structure motifs as found in several intergenic regions. The genomic organization is depicted and red arrows represent ncRNA genes. Examples of secondary structure motifs found in several ncRNAs as predicted by contrafold [20] and RNAFold [21]. (TIF) [file pgen.1002782.s006.tif]

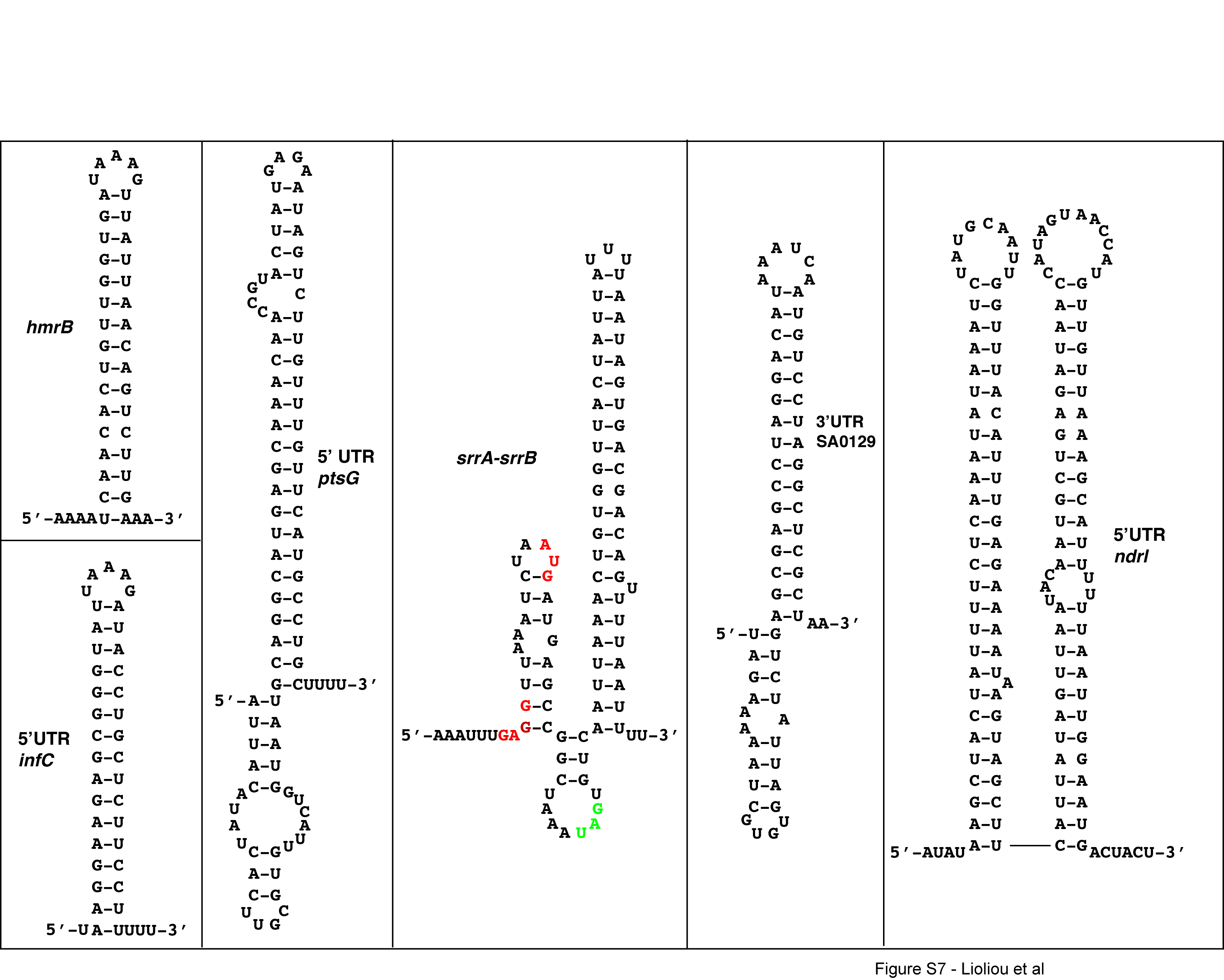

Supplement: Figure S7 — Secondary structures of mRNA fragments co-immunoprecipitated with RNase III. The RNA fragment of srrA-srrB mRNA co-immunoprecipitated with the mutant enzymes corresponded to the translational coupling site. The stop codon of srrA is depicted in green, the start codon, and the Shine and Dalgarno sequence (SD) of srrB are given in red. UTR stands for untranslated region. The secondary structure models were predicted using contrafold [20] and RNAFold [21]. (TIF) [file pgen.1002782.s007.tif]
