# Supplementary material for: Global Regulatory Functions of the Staphylococcus aureus Endoribonuclease III in Gene Expression
Source: PLoS Genet. 2012 Jun 28;8(6):e1002782. doi: 10.1371/journal.pgen.1002782 (PMC3386247; doi:10.1371/journal.pgen.1002782)
Supplement: Table S1 — Read numbers and Mapping statistics. NC_002745 = S. aureus N315 genome; NC_003140 = S. aureus N315 plasmid. Immunoprecipitation experiments were carried out in RN6390 strain (wild-type and referent strain) as a control, and in the mutant Δrnc strain transformed with a plasmid expressing either the WT flag-tagged RNase III (IP_EL79), the mutant D63A flag-tagged RNase III (IP_EL80) or the mutant E135A flag-tagged RNase III (IP_E78). Total RNAs were prepared from cells grown at the exponential phase (4 h) and late exponential phase (6 h). * Other stable ncRNAs referred to tmRNA, 4.5S RNA, 6S RNA and RNase P. (DOCX) [file pgen.1002782.s008.docx]

**Table S1: Read numbers and Mapping statistics**

|  | **Exponential growth** | | | | **Late-exponential phase growth** | | | |
| --- | --- | --- | --- | --- | --- | --- | --- | --- |
|  | **IP_RN6390** | **IP_EL78 (E135A)** | **IP_EL79**  **(WT)** | **IP_EL80**  **(D63A)** | **IP_RN6390** | **IP_EL78**  **(E135A)** | **IP_EL79**  **(WT)** | **IP_EL80**  **(D63A)** |
| **Total number of reads** | 43910 | 66311 | 57802 | 58935 | 54400 | 53483 | 51529 | 55865 |
| **<18 nt** | 17521 | 14997 | 28488 | 8193 | 23441 | 8794 | 15764 | 5515 |
| **>= 18 nt** | 26389 | 51314 | 29314 | 50742 | 30959 | 44689 | 35765 | 50350 |
| **Mapped Reads** | 3850 | 29096 | 5264 | 43862 | 7059 | 38733 | 27427 | 38750 |
| **Total number of mappings** | 14155 | 100068 | 16534 | 126190 | 16364 | 234324 | 112919 | 161548 |
| **Uniquely mapped reads** | 672 | 8120 | 1861 | 7258 | 3451 | 3120 | 7205 | 3996 |
| **Mapped reads in NC_002745.fa** | 3849 | 29002 | 5253 | 43812 | 7058 | 38720 | 27398 | 38738 |
| **Mapped reads in NC_003140.fa** | 2 | 94 | 11 | 50 | 1 | 13 | 29 | 12 |
| **Mappings in NC_002745.fa** | 14153 | 99974 | 16523 | 126140 | 16363 | 234311 | 112886 | 161535 |
| **Mappings in NC_003140.fa** | 2 | 94 | 12 | 50 | 1 | 13 | 33 | 13 |
| **>= 18 nt but not mapped** | 7057 | 38707 | 27369 | 38727 | 23900 | 5956 | 8338 | 11600 |
| **Number of reads for rRNA, tRNA** | 3330,7 (86,5%) | 21898,4 (75,5%) | 3471,5 (66,1%) | 39508,5 (90,2%) | 5939  (84,1%) | 32776,5  (84,7%) | 20539,3  (75%) | 36094,4  (93,2%) |
| **Numbers of reads for other stable ncRNAs*** | 21  (0,6%) | 99,5 (0,34%) | 5,5 (0,1%) | 47 (0,11%) | 24  (0,34%) | 58  (0,15%) | 66  (0,24%) | 71  (0,18%) |
| **Numbers of reads for mRNAs** | 277,6  (7,2%) | 3615,3  (12,5%) | 767,8 (14,6%) | 2034,9 (4,6%) | 361,3 (5,1%) | 1171,3 (3%) | 2822,9 (10,3%) | 1640,6 (4,2%) |
| **Numbers of reads for sense sRNAs** | 47,6  (1,2%) | 1308,8  (4,5%) | 174,8  (3,3%) | 696,6 (1,6%) | 60,1  (0,9%) | 2974,6 (7,7%) | 1199,6  (4,4%) | 357,8 (0,9%) |
| **Numbers of reads for antisense to rRNA and tRNA** | 4 | 22 | 3,7 | 55,6 | 3 | 36,7 | 26 | 20,3 |
| **Numbers of reads for antisense to mRNAs** | 150 | 115,2 | 665,9 | 1060,5 | 575,9 | 182,7 | 1522,3 | 351,2 |
| **Number of reads for antisense to sRNAs** | 7,7 | 386,7 | 137,1 | 238,1 | 36 | 163,2 | 652,1 | 171,2 |
| **% of antisense RNAs** | 3,9% | 3,85% | 12,68% | 2,42% | 8,16% | 0,47% | 5,56% | 0,91% |

NC_002745 = *S. aureus* N315 genome; NC_003140 = *S. aureus* N315 plasmid. Immunoprecipitation experiments were carried out in RN6390 strain (wild-type and referent strain) as a control, and in the mutant ∆*rnc* strain transformed with a plasmid expressing either the WT flag-tagged RNase III (IP_EL79), the mutant D63A flag-tagged RNase III (IP_EL80) or the mutant E135A flag-tagged RNase III (IP_E78). Total RNAs were prepared from cells grown at the exponential phase (4h) and late exponential phase (6h). * Other stable ncRNAs referred to tmRNA, 4.5S RNA, 6S RNA and RNase P.
