# Supplementary material for: Global Regulatory Functions of the Staphylococcus aureus Endoribonuclease III in Gene Expression
Source: PLoS Genet. 2012 Jun 28;8(6):e1002782. doi: 10.1371/journal.pgen.1002782 (PMC3386247; doi:10.1371/journal.pgen.1002782)
Supplement: Table S6 — Transcriptional start sites (TSS) of several RNAs that were co-immunoprecipitated with RNase III. (a) Numbering according to N315 genome. (b) Start site as defined by primer extension (PE) analysis using the indicated primer (+1 site detected in wt and Δrnc strains was identical unless otherwise indicated). (c, d) Start site and size of RNAs as defined by deep sequencing data, respectively. Only the longest RNA fragment pulled down by either of the two mutant proteins was indicated. (d) Size of as-cspA according to the fragment pulled down with RNase III from samples prepared at the exponential phase of growth (EP); a small fragment was also detected starting at 1408841 from samples prepared at the late exponential phase of growth (SP). (e) References are given for the non coding RNAs for which the exact ends were not mapped in previous studies. Where more than one +1 sites were detected, the main site is indicated in bold letters. In the case of tagG and tagH mRNAs, the main +1 site was detected by 5′-3′ RACE but could correspond to processed RNA; in the case of rnc the main +1 start site is estimated based on the size of the sequenced fragment). nd: not detected. (DOCX) [file pgen.1002782.s013.docx]

| **RNA** | **Strand** | **Start site^a^** | |  |  | **Size (nts)** | **Primer** | **Ref.^e^** |
| --- | --- | --- | --- | --- | --- | --- | --- | --- |
|  |  | **PE^b^** | | | **HTS^c^** |  |  |  |
|  |  | **TSS** | | |  |  |  |  |
|  |  | **WT** | ***∆rnc*** | |  |  |  |  |
| *tagG* | + | 686766 | identical | | 686733 |  | 408 and 332 |  |
|  |  | **686876** | identical | |  |  |  |  |
|  |  | nd | 686941 | |  |  |  |  |
| *tagH* | - | **686716** | identical | | 686980 |  | 410 and 334 |  |
|  |  | 686851 | nd | |  |  |  |  |
|  |  | 686970 | identical | |  |  |  |  |
| SAS028 | + | 1006178 | identical | | EP: 3’ UTR detected  SP: 5’UTR detected starting at 1006178 | 301 | 388 |  |
| Sau-02/teg102  (as SAS028) | - | 1006480 | identical | | 1006481 | 246 | 387^e^ | Abu-Qatouseh et al. [8]; Beaume et al. [9] |
| *rnc* | + | **~1216196** | - | | see text for details |  | 380 |  |
|  |  | 1216572 |  |  |  |  |  |  |
| as *cspA* | + | 1409025 | identical | | 1409015 | 131^d^ | 367 |  |
| *cspA* | - | 1409085 | 1409145 | | see text for details |  | 378 and 379 |  |
| SprFG3, Teg19a | + | **2206385** | identical | | 2206385 | 322 | 257 | Pichon and Felden [15]; Beaume et al. [9] |
|  |  | 2206484 | identical | |  |  |  |  |
| SprFG3, Teg19b | - | 2206557 | identical | | 2206708 | 328 | 256 | Pichon and Felden [15]; Beaume et al. [9] |
|  |  | **2206708** | identical | |  |  |  |  |
| RsaO | + | 2370170 | identical | | 2370165 | 215 | 416 |  |

**Table S6: Transcriptional start sites (TSS) of several RNAs that were co-immunoprecipitated with RNase III.**

(a) Numbering according to N315 genome. (b) Start site as defined by primer extension (PE) analysis using the indicated primer (+1 site detected in wt and ∆*rnc* strains was identical unless otherwise indicated). (c, d) Start site and size of RNAs as defined by deep sequencing data, respectively. Only the longest RNA fragment pulled down by either of the two mutant proteins was indicated. (d) Size of as-*cspA* according to the fragment pulled down with RNase III from samples prepared at the exponential phase of growth (EP); a small fragment was also detected starting at 1408841 from samples prepared at the late exponential phase of growth (SP). (e) References are given for the non coding RNAs for which the exact ends were not mapped in previous studies. Where more than one +1 sites were detected, the main site is indicated in bold letters. In the case of *tagG* and *tagH* mRNAs, the main +1 site was detected by 5’-3’ RACE but could correspond to processed RNA; in the case of *rnc* the main +1 start site is estimated based on the size of the sequenced fragment). nd: not detected.
