# Supplementary material for: Global Regulatory Functions of the Staphylococcus aureus Endoribonuclease III in Gene Expression
Source: PLoS Genet. 2012 Jun 28;8(6):e1002782. doi: 10.1371/journal.pgen.1002782 (PMC3386247; doi:10.1371/journal.pgen.1002782)
Supplement: Table S7 — Strains and plasmids used in this study. (DOCX) [file pgen.1002782.s014.docx]

| **Strains** | **Relevant characteristics** | **Reference/source** |
| --- | --- | --- |
| *E. coli* strains |  |  |
| DH5a | Cloning strain | Laboratory stock |
| XL1-blue | Cloning strain | Laboratory stock |
| M15[pREP4] | Strain suitable for overexpression of proteins | Qiagen |
|  |  |  |
| *S. aureus* strains |  |  |
| NCTC8325-4 | NCTC8325 cured of three prophages | Novick [13] |
| RN4220 | Restriction mutant of 8325-4 | Kreiswirth et al. [11] |
| RN6390 | Derivative of 8325-4, *agr* positive | Peng et al. [14] |
| LUG774 | RN6390: Δ*rnc* region::*cat86* | Huntzinger et al. [1] |
| EL78 | pEL72→LUG774 (Δ*rnc* p_cad_-*rnc*E135A) | This work |
| EL79 | pEL73→LUG774 (Δ*rnc* p_cad_-*rnc*) | This work |
| EL80 | pEL74→LUG774 (Δ*rnc* p_cad_-*rnc*D63A) | This work |
|  |  |  |
|  |  |  |
| Plasmids |  |  |
| *E. coli* plasmids |  |  |
| pLUG515 | pQE30::*rnc* (nts980-1670) | Huntzinger et al. [1] |
| pEL70 | pQE30::*rnc* E135A | This work |
| pEL71 | pQE30::*rnc* D63A | This work |
| pUC18_T7_RsaA | T7 promoter/RsaA | Geissmann et al. [10] |
| pUC18_T7_RsaH | T7 promoter/RsaH | Geissmann et al. [10] |
| pUT7-*spa* | T7 promoter/*spa* | Huntzinger et al. [1] |
| *E. coli-staphylococcal* shuttle plasmids |  |  |
| pCN51 | Vector suitable for expression of proteins in *S. aureus* | Charpentier et al. [2] |
| pEL72 | pCN51 derivative for expression of RNaseIIIE135A;the protein contains N-term His tag and C-term Flag tag | This work |
| pEL73 | pCN51 derivative for expression of the wt RNaseIII;the protein contains N-term His tag and C-term Flag tag | This work |
| pEL74 | pCN51 derivative for expression of RNaseIIID63A;the protein contains N-term His tag and C-term Flag tag | This work |

**Table S7: Strains and plasmids used in this study.**
