# Supplementary material for: Global Regulatory Functions of the Staphylococcus aureus Endoribonuclease III in Gene Expression
Source: PLoS Genet. 2012 Jun 28;8(6):e1002782. doi: 10.1371/journal.pgen.1002782 (PMC3386247; doi:10.1371/journal.pgen.1002782)
Supplement: Table S8 — Oligonucleotides used in this study. (a) With bold letters the mutated nucleotides are indicated; (b) With italics the enzyme restriction sites are indicated; (c) With small letters the sequence hybridizing to pQE30 vector is indicated; (d) With small bold letters the Flag-tag is indicated; (e) Underlined is the T7 promoter sequence. Cand is for candidate RNAs: Cand1 is for SprFG3; Cand3: RsaX28; Cand4: RsaN; Cand5a/b: asSAS028 (SAU-02); Cand6: RsaX31; Cand7: RsaX41; Cand8: RsaL; Cand9: RsaM; for additional details see Table S1. (DOCX) [file pgen.1002782.s015.docx]

| **No.** | **Name** | **Sequence** |
| --- | --- | --- |
| 16 | Cold shock | 5’-AACAACGTTTGCAGCTTGTG-3’ |
| 69 | rnc rev | 5’-TCCAAATACAATGCCCCAAT-3’ |
| 70 | hu-for | 5’-cagatttaatcaatgcagttgcaga-3’ |
| 71 | hu-rev | 5’-taatgctttaccagctttgaatgct-3’ |
| 94 | ^a^rncD45R | 5’-CCGTCAATTCTAATACCGC**TGC**ACCCAAAAACTCTAAACG-3’ |
| 95 | rncE117F | 5’-CATTAATATCAGATGCATTC**GCA**GCATTTATTGGGGCATTG-3’ |
| 96 | rncE117R | 5’-CAATGCCCCAATAAATGC**TGC**GAATGCATCTGATATTAATG-3’ |
| 97 | rncD45F | 5’- CGTTTAGAGTTTTTGGGT**GCA**GCGGTATTAGAATTGACGG-3’ |
| 105 | 5SrRNA-rev | 5’-aacaggtgtgacctccttgc-3’ |
| 114 | ^b, c^RNASEIIIF | 5’-AAAA*CTGCAG*ttcattaaagaggagaaattaactatg-3’ |
| 115 | ^d^RNASEIIIR | 5’-CCG*GAATTC*TTA**cttgtcatcgtcgtccttgtagtc**TTTAATTTGTTTTAATTGCTTATAGGC-3’ |
| 170 | ^e^RNase-T7dist | 5’-CGC*GGATCC*GAAATTAATACGACTCACTATAGGGAGATAAATCTTACATCTGGGTCG-3’ |
| 171 | RNase-T7prox | 5’-CGC*GGATCC*GAAATTAATACGACTCACTATAGGGAGAGCAACACATAAAGGAGATAAAATAATG-3’ |
| 214 | clpP-f | 5’- TAATACGACTCACTATAGG ATGAATTTAATTCCTACAGTTATTG-3’ |
| 215 | clpP-r | 5’-TTATTTTGTTTCAGGTACCATCAC-3’ |
| 217 | groES-r | 5’-TTATTCAATTACCGCTAAAATATCTTC-3’ |
| 220 | tagG +st | 5’- CATCAATGATAACAATGATATTTAAAAACTAAAC-3’ |
| 221 | tagG-r | 5’- TTACAAGAAGTCTGCAAATTGATCTC-3’ |
| 222 | tagH –st | 5’- CATTATAGTATACAATCATGTAAGTTG-3’ |
| 225 | ssaA-r | 5’-TTAATGGATGAATGCATAGCTAG-3’ |
| 226 | ssaA-f | 5’- GTAAAACTTAATTTTAAGGAGGAG-3’ |
| 231 | lsp-f | 5’- GGACGATTGATTGGAGGAACG-3’ |
| 232 | lsp-r | 5’-TTACTTAACCTCCTTCTCC-3’ |
| 247 | rRNA7-T7 | 5’-TAATACGACTCACTATAGG GAATAAGCTGGAGGGG -3’ |
| 248 | rRNA8 | 5’- TGGCTGGGCTAGCTGGATTCG-3’ |
| 250 | rRNA9 | 5’- GTGCAAGTAGTGAGCCATAGAGG-3’ |
| 251 | tRNA-Arg | 5’-TAATACGACTCACTATAGG GTCCTGGTAGCTCAGCTGG-3’ |
| 252 | tRNA-Arg-r | 5’-GAGCCCCGTAATCACGG-3’ |
| 256 | Cand1+ | 5’-GCCTTCAAAATGCCGGTCAAAGCG-3’ |
| 257 | Cand1- | 5’- CGCTTTGACCGGCATTTTGAAGGC-3’ |
| 258 | Cand2+ | 5’- GATTAAGATGGTAGATTTAGTC-3’ |
| 259 | Cand2- | 5’- GACTAAATCTACCATCTTAATC-3’ |
| 260 | 4.5S-r | 5’- TCTTCAAACACTACGTGCTTTGGGC-3’ |
| 261 | SAS025 forward | 5’-AAA*GAATTC*TAATACGACTCACTATAGGGGAATGACATGAAACACCTCG-3’ |
| 262 | SAS025 rev | 5’-AAA*GGATCC*GTTATTGTGATGAATCTTTCGG-3’ |
| 263 | FMN forward | 5’-AAA*GAATTC*TAATACGACTCACTATAGGGGTAAAATTCATCTTCGGGGTC-3’ |
| 264 | FMN rev | 5’-AAA*GGATCC*GCGCTCAACATACTTATTGTG-3’ |
| 265 | T-Box forward | 5’-AAA*GAATTC*TAATACGACTCACTATAGGGGAAGACAAGATGATCTAATC-3’ |
| 266 | T-Box rev | 5’-AAA*GGATCC*CCAACAAACATTTATGTCTG-3’ |
| 267 | SAS057 forward | 5’-AAA*GAATTC*TAATACGACTCACTATAGGGCGATGTTGAGGTGAAATAAATTG-3’ |
| 268 | SAS057 rev | 5’-AAA*GGATCC*CTATTTACTATTTTTACGTCG-3’ |
| 270 | hu-T7 | 5’-TAATACGACTCACTATAGG GTGTATTATTACACATATACGGC-3’ |
| 272 | 6S +st | 5’-CGAACAACTCAGATGACTATC-3’ |
| 273 | SAS050-r | 5’-GAAACGCATAAAAATACGC-3’ |
| 275 | Val-5S | 5’-GCTGAGCTAATTCTCCGATTTAAAACTGCCTGGC-3’ |
| 276 | 16S-Gly | 5’-CAATGTTCATTAAAATTATCTATGGAGCGGG-3’ |
| 277 | Asn-5S | 5’-GAGCTACTGTGGATTAATATTATGCCTGGC-3’ |
| 278 | Ala-16S | 5’-GTCATTCAGTTTTCAATGTTCATCGTG-3’ |
| 279 | 16S-Ile | 5’-GCTATAGGCCCATTTTTTTGAATG-3’ |
| 285 | clpP-f2NEW | 5’- TAATACGACTCACTATAGGGG CATATCTCTGTACTATGAG-3’ |
| 286 | cspA-T7NEW | 5’- TAATACGACTCACTATAGGGG CTGCACTATAATACAAGTTAAG-3’ |
| 287 | groES-f NEW | 5’- TAATACGACTCACTATAGG GGTTATGACTTGAAATTTTGACC-3’ |
| 288 | 4.5S-T7 | 5’-TAATACGACTCACTATAGGGG ATCTTGCCGTGCTAGGTGGGGAGGTAGC-3’ |
| 289 | 4.5S-rev | 5’- TAAAAAACCGTGCACCTAAGCATCGAACG -3’ |
| 291 | secY-T7 | 5’-TAATACGACTCACTATAGGGG ATGATTCAAACCCTTGTG-3’ |
| 292 | secY-rev | 5’-TTTAAAGGTAGATAAGTTGCCTGTG-3’ |
| 302 | purine ribo-T7 | 5’- TAATACGACTCACTATAGGGG TAATTTACATAAACTCATATAATC-3’ |
| 303 | SAM ribo-T7 | 5’- TAATACGACTCACTATAGG GGACTTGGCCCTG-3’ |
| 304 | SAM rev | 5’-CTTCTCACCCTATTGAAAAG-3’ |
| 307 | SAS050-fT7 NEW | 5’- TAATACGACTCACTATAGGGG TGTTATAATTTCATTAATTTTAATTATGTGG -3’ |
| 309 | eno-rev | 5’-GGTACTAATGCACGACCAAATGCG-3’ |
| 311 | purine rev | 5’- ATGGTTACAATTTAATGGC-3’ |
| 312 | cand3-T7trans | 5’- TAATACGACTCACTATAGGGG TGGCAGTAATAATGAATTTG-3’ |
| 313 | cand3-T7trans  rev | 5’- CCAAAGATA GTTGTCACAATAGTGTG-3’ |
| 314 | cand4-T7trans | 5’- TAATACGACTCACTATAGGGG TAATACTGTGTTTTATCTGCG-3’ |
| 315 | cand4-T7trans  rev | 5’-  GTGCGTTTAAAAGTTAACATTGTC-3’ |
| 316 | cand5a-T7trans | 5’- TAATACGACTCACTATAGGGG GGCATTACTTAACACTTTTGAAAGG-3’ |
| 317 | cand5a-T7trans  rev | 5’-GTAAAAAGACGACATGCAGG-3’ |
| 318 | SAS028-T7 trans  rev | 5’-GGCTATCTGAGTAAAGGGGGG-3’ |
| 319 | cand 6-T7 trans | 5’- TAATACGACTCACTATAGGGG AAAAAACACGCATCACTTAAATAAG-3’ |
| 320 | cand 6-T7 trans  rev | 5’-GAACATTGATTTATGTCCCAGCC-3’ |
| 321 | cand 7-T7 trans | 5’- TAATACGACTCACTATAGGGG TGAATAGTGAGTAATCAGCAATC-3’ |
| 322 | cand 7-T7 trans rev | 5’-ACCACTTTAAATCAATAAGTGTCTC-3’ |
| 323 | cand 8-T7 trans | 5’- TAATACGACTCACTATAGGGG GATGCATAAAAAAAGAACTACGC-3’ |
| 324 | cand 8-T7 trans rev | 5’-GTGAGGAAAGAGACTTATAGG-3’ |
| 325 | cand 9-T7 trans | 5’- TAATACGACTCACTATAGGGG AAAAAAAGAGGCCAGATGACG-3’ |
| 326 | cand 9-T7 trans rev | 5’-CAAATTCCCGGTAACCATTCC-3’ |
| 327 | cand 10-T7 trans | 5’- TAATACGACTCACTATAGGGG GTTAAGGCTTTTTATTTATGTG-3’ |
| 328 | cand 10-T7 trans  rev | 5’-CTGCATTTTTTAATGCATTCGT-3’ |
| 329 | ctsR-T7trans | 5’-TAATACGACTCACTATAGGGG GTCAAAGAAGGTCAAAAAGGGGTG-3’ |
| 330 | ctsR-T7trans  rev | 5’-AGCTTGTTGTTGAGAAATAG-3’ |
| 331 | tagG+ T7trans | 5’-TAATACGACTCACTATAGGGG GACCTTCCTTATTCACATTTATC-3’ |
| 332 | tagG+ T7trans rev | 5’-GTTGTAAATACTATCACTATACA-3’ |
| 333 | tagG minus T7trans | 5’- TAATACGACTCACTATAGGGG GTTGTAAATACTATCACTATACA-3’ |
| 334 | tagG minus T7trans rev | 5’- GACCTTCCTTATTCACATTTATC-3’ |
| 335 | ssaA+ T7trans | 5’- TAATACGACTCACTATAGGGG TTAATGGATGAATGCATAGC-3’ |
| 336 | ssaA+ T7trans  rev | 5’-AGCATTTGCAATAACAGCAAC-3’ |
| 337 | ssaA minus T7trans | 5’- TAATACGACTCACTATAGGGG AGCATTTGCAATAACAGCAAC-3’ |
| 338 | ssaA minus T7trans  rev | 5’-TTAATGGATGAATGCATAGC-3’ |
| 339 | tnp-T7trans | 5’- TAATACGACTCACTATAGGG GCA GCTCAACGAGCTG-3’ |
| 340 | tnp-T7trans rev | 5’-CTTTGAATGGACGAACCA-3’ |
| 341 | lsp+ T7trans | 5’- TAATACGACTCACTATAGGGG GAGTTGTCTTATGGACGATTGATTGG-3’ |
| 342 | lsp+ T7trans rev | 5’-CTAAGGCTATTAATATAATAATGG-3’ |
| 343 | SAS028-T7 trans | 5’- TAATACGACTCACTATAGGGG TATTGCTTAAATTTATTATTGCTAC-3’ |
| 344 | cspA short-T7 | 5’-TAATACGACTCACTATAGGGG GCGAATAAGCATATTGAATG-3’ |
| 345 | secY-rev2 | 5’-CCATTACCAACACCGAACTGAGTGA-3’ |
| 346 | secY-rev3 | 5’-GGAATGCCATACCTATAGATTGG-3’ |
| 347 | hmrB-T7 | 5’- TAATACGACTCACTATAGGGG GTCTATGTATAGGCATTTTTAAAGGAGGTG-3’ |
| 348 | lctP-T7 | 5’-TAATACGACTCACTATAGGGG CACATTAATCTCACTGTTCTG-3’ |
| 349 | hmrB-rev | 5’- AAAAACTGAGTCGACAATACTGACG-3’ |
| 350 | cand4 | 5’-  TAATACGACTCACTATAGGGG GTGCGTTTAAAAGTTAACATTGTC-3’ |
| 351 | cand4rev | 5’- TAATACTGTGTTTTATCTGCG-3’ |
| 352 | lctP-rev | 5’- GCTAACATTGTTAATAACGGTGG-3’ |
| 354 | 5 IGR-T7 | 5’ TAATACGACTCACTATAGGGG GTAACATATCTTTTATTTAATTGTC-3’ |
| 355 | 5 IGR rev | 5’-TCATTGCGACTTCTATATCAATG-3’ |
| 357 | SAS28 rev2 | 5’-CTTAACACTTTTGAAAGGAAAAGCC-3’ |
| 358 | 5-T7 | 5’-TAATACGACTCACTATAGGGG CGCAGTGCATAAATAGACG -3’ |
| 359 | RsaA-rev | 5’-TAAAAAAATTCCAAGCTTATCGG-3’ |
| 362 | RsaE-T7 | 5’- TAATACGACTCACTATAGGGG ATGAAATTAATCACATAACAAACATAC-3’ |
| 363 | RsaF-rev | 5’-TATATTAATCGTTAATGTATAAAAAGTGC-3’ |
| 364 | RsaA-T7 | 5’- TAATACGACTCACTATAGGGG GTTAACCATTACAAAAATTGTATAGAG-3’ |
| 365 | RsaAext-T7 | 5’- TAATACGACTCACTATAGGGG ATAAAAAGGGAAAATTATTGAAAAAAATTCGAC-3’ |
| 367 | cspA asRNA-rev | 5’-GTTAAGCAGATGATTATTCCATATTGC-3’ |
| 368 | cspAT7 +str | 5’-TAATACGACTCACTATAGGGG CAACGTTTGCAGCTTGTGGAC-3’ |
| 370 | hu asRNA-rev | 5’-CATAATGATATAAACATATCATTATCAAACC-3’ |
| 371 | huT7 +str | 5’-TAATACGACTCACTATAGGGG CAGCATCTTTTAATGCTTTACCAGC-3’ |
| 372 | secY asRNA-rev | 5’-TATTAATAGCACTAGTTTTGACAGC-3’ |
| 373 | secYT7 +str | 5’-TAATACGACTCACTATAGGGG CATTGTTTCAATCGCTACACC-3’ |
| 374 | RsaA asRNA-fw | 5’-TAATACGACTCACTATAGGGG CAAAGTACACTTTGCTCATAGC-3’ |
| 376 | RsaA asRNA-rev | 5’-CCATTACAAAAATTGTATAG-3’ |
| 378 | cspA rev2 uniq | 5’- AAAATGTACGAATACGTCATTTTCTC-3’ |
| 379 | cspA rev3 uniq | 5’-CAACTACTTCAAACTCAACAGC-3’ |
| 380 | rnc rev new | 5’- GCATCTGATATTAATGATGGTC-3’ |
| 381 | 5S-T7trans | 5’- TAATACGACTCACTATAGGGGATTTGTCATTTGCCTGGC -3’ |
| 382 | 5S-T7trans rev | 5’-GTAAGTTATTTTGTCTGGTGGCTATAGC-3’ |
| 383 | SA0702(llm) rev | 5’-CCAATCATTAAAGCCCCACTATCACC-3’ |
| 384 | tagH rev | 5’-CAATAATCTTAGGTGTCATCGC-3’ |
| 385 | as llm | 5’-CGTATAGTTGTAAATATGATATCATTGATTGAGC-3’ |
| 386 | cand1 T7rev | 5’-GAGCAAGTTGGATAGATGGTGG-3’ |
| 387 | SAS28-3’UTR | 5’-CCATCATTTCAAAACTTTGAC-3’ |
| 388 | 5a unique | 5’-TATTGCTTAAATTTATTATTGCTAC-3’ |
| 389 | cand1 T7 | 5’-TAATACGACTCACTATAGGGG GTAGTAAGTAGAAGCAAAAGATG-3’ |
| 390 | as cand1 T7 | 5’-TAATACGACTCACTATAGGGG GAGCAAGTTGGATAGATGGTGG-3’ |
| 391 | as cand1 T7rev | 5’-GTAGTAAGTAGAAGCAAAAGATG-3’ |
| 392 | mRNA tagG rev | 5’- TTGAAAGTGTGGTTTAATGG-3’ |
| 393 | mRNA tagG T7 | 5’-TAATACGACTCACTATAGGGG CAAGAAGTCTGCAAATTGATCTC-3’ |
| 394 | mRNA tagH T7 | 5’-TAATACGACTCACTATAGGGG GGTTTATTTAATAACGAAGCGGG-3’ |
| 395 | mRNA tagH rev | 5’-CAAATAAAGAACGTATGAAAGATGC-3’ |
| 396 | llm T7 | 5’-TAATACGACTCACTATAGGGG CTAATAGTTTATGATGCAAATGCG-3’ |
| 397 | llm T7rev | 5’-GGTTACATTATTACTAGTTGC-3’ |
| 398 | llm 5UTR T7rev | 5’-CACCTTCATCGATATTAATTG-3’ |
| 399 | SA0701 5UTR T7 | 5’-TAATACGACTCACTATAGGGG CACCTTCATCGATATTAATTG-3’ |
| 400 | SA0701 5UTR T7rev | 5’-GTTGCCTTAAATGATATGATG-3’ |
| 401 | llm 5UTR T7 | 5’-TAATACGACTCACTATAGGGG GTTGCCTTAAATGATATGATG-3’ |
| 404 | T7-Ala-16S | 5’-TAATACGACTCACTATAGGGG GCTAGTCTCCACCATTTATTTTTACACG-3’ |
| 405 | 16S-rev1 | 5’-CTCTCCATAAAAATTATGATGTTTG-3’ |
| 406 | 16S-rev2 | 5’-CCGTTCGCTCGACTTGC-3’ |
| 407 | UTR tagGH RACE | 5’-GATAGCATCCATGTATAGTG-3’ |
| 408 | tagG PE | 5’-CCCAAGCCACACCTAAATAG-3’ |
| 409 | tagG RACE | 5’-gcaattGGTGCGTACTTACA |
| 410 | tagH PE | 5’-GTTTGGGAATGAGCGCATC-3’ |
| 411 | tagH RACE | 5’-GAAACAAACTCGATGAGGCCC-3’ |
| 412 | SAM-T7NEW | 5’-TAATACGACTCACTATAGGGG CATATTTCTTATTGTGAGAAG-3’ |
| 413 | SAM rev NEW | 5’-CTGTGTAATTTGTCATTGttc-3’ |
| 414 | SAS28T7 2.11 | 5’- TAATACGACTCACTATAGGGG GCAATACGAAGATTCAAGGCATGC-3’ |
| 415 | Cand2-T7 | 5’- TAATACGACTCACTATAGGGG GATTAAGATGGTAGATTTAGTC-3’ |
| 416 | Cand2-rev | 5’-GAATATGTTGTTTGTTTAATG-3’ |
| 417 | as cspA short | 5’-TAATACGACTCACTATAGGGG CCTTGTTTCATAATCTGAAACCTCC-3’ |
| 418 | as cspA rev NEW | 5’-CTGCACTATAATACAAGTTAAG-3’ |
| 419 | AgrA-T7 | 5’-TAATACGACTCACTATAGGGG GAAAATTTTCATTTGCGAAGACG-3’ |
| 420 | as AgrA rev | 5’-GAAAATTTTCATTTGCGAAGACG-3’ |
| 421 | as AgrA-T7 | 5’-TAATACGACTCACTATAGGGG TCTCACCGATGCATAGCAGTG-3’ |
| 422 | glmS +st rev | 5’-GGAATAGTACGCGTAAATTATG-3’ |
| 423 | glmS -st T7 | 5’-TAATACGACTCACTATAGGGG GTTAATAAGATCGCCAGAAATTG-3’ |
| 424 | as glmS rev | 5’-GTTAATAAGATCGCCAGAAATTG-3’ |
| 425 | as glmS T7 2 | 5’-TAATACGACTCACTATAGGGG CGCGATACCTGCAGAGTC-3’ |
| 426 | def +st rev | 5’-GTGTAAATATCTACAAAGTTAACCAAC-3’ |
| 427 | def – st T7 | 5’-TAATACGACTCACTATAGGGG CTGTTGACAGTTACGTC-3’ |
| 428 | as def rev | 5’-CTGTTGACAGTTACGTC-3’ |
| 429 | as def T7 2 | 5’-TAATACGACTCACTATAGGGG GAACGTAAACCATATCGTTTCGC-3’ |
| 430 | AgrA rev | 5’-TCTCACCGATGCATAGCAGTG-3’ |
| 431 | as glmS T7 | 5’-TAATACGACTCACTATAGGGG GGAATAGTACGCGTAAATTAT-3’ |
| 432 | as def T7 | 5’-TAATACGACTCACTATAGGGG GTGTAAATATCTACAAAGTTAACCAAC-3’ |
| 433 | as def rev 2 | 5’-CATTAGAGATGGTCATCCAAC-3’ |
| 502 | SA2097-T7-fw | 5’-TAATACGACTCACTATAGGGGATGAAAAAATTAGTAACAGCAACTACGTTAAC-3’ |
| 503 | SA2097-rev | 5’-TTAAATATGGATGTAGTTGTAG-3’ |

**Table S8: Oligonucleotides used in this study.**

(a) With bold letters the mutated nucleotides are indicated; (b) With italics the enzyme restriction sites are indicated; (c) With small letters the sequence hybridizing to pQE30 vector is indicated; (d) With small bold letters the Flag-tag is indicated; (e) Underlined is the T7 promoter sequence. Cand is for candidate RNAs: Cand1 is for SprFG3; Cand3: RsaX28; Cand4: RsaN; Cand5a/b: asSAS028 (SAU-02); Cand6: RsaX31; Cand7: RsaX41; Cand8: RsaL; Cand9: RsaM; for additional details see Table S1.
