# Supplementary material for: Global Regulatory Functions of the Staphylococcus aureus Endoribonuclease III in Gene Expression
Source: PLoS Genet. 2012 Jun 28;8(6):e1002782. doi: 10.1371/journal.pgen.1002782 (PMC3386247; doi:10.1371/journal.pgen.1002782)
Supplement: Text S1 — Supplementary Material and Methods. (DOCX) [file pgen.1002782.s016.docx]

**Supplementary Material and Methods**

**Strains and plasmids**

To introduce mutations E135A and D63A into the *S. aureus* RNase III enzyme, the Quickchange XL Site-directed mutagenesis protocol (Stratagene) was followed. The vector pLUG515 [1] was used as a template with primer pairs 95/96 and 94/97 resulting in vectors pEL70 (pQE30::*rnc E135A*) and pEL71 (pQE30::*rnc D63A*), respectively. For overexpression and purification of the N-terminal His-tagged WT and mutant proteins, the plasmids were transformed into *E. coli* strain M15[pREP4] (Qiagen). To express the wild type and mutant versions of RNase III into *S. aureus* the shuttle vector pCN51 [2] was used. To this end, primers 114/115 were used carrying PstI and EcoRI sites, respectively. As templates, vectors pLUG515, pEL70 or pEL71 were employed to amplify the wt or the mutant versions of RNase III, respectively. The inserts were cloned into pCN51 digested with PstI/EcoRI. This gave raise to plasmids pEL72, pEL73 and pEL74 carrying RNase IIIE135A, RNase III (wt) and RNase IIID63A, respectively (Table S6). In all cases, the proteins carried a N-term His-tag and a C-term Flag-tag. A Cd^2+^ inducible promoter present on the plasmid drove expression of the proteins. The cloned fragments carried no 5’ UTR and for translation of the proteins the Shine and Dalgarno sequence present in the original pQE30 vector was employed. Plasmids pEL72, -73 and -74 were transformed into electro-competent cells of *S. aureus* strain RN4220 and then into strain LUG774 (∆*rnc*), as it was previously described [1].

**Overexpression and purification of RNase III**

Overexpression and purification of wild-type and mutant RNase III from *E. coli* cells were performed as described previously [3]. Briefly, after cell growth and grinding, crude extract was prepared in 25 mM Tris-HCl pH 8.0, 150 mM KCl containing a protease inhibitor cocktail, and was incubated with the Ni^2+^-beads (Qiagen) for 1h at 4°C. The beads were then successively washed with 25 mM Tris-HCl pH 8.0 in the presence of 400 mM KCl, and with the same buffer containing 150 mM KCl. The enzyme was eluted in the same buffer using increasing concentrations of imidazole (from 50 mM to 800 mM). After concentration and dialysis, the RNase III was kept in a storage buffer containing 50 % glycerol. Mass spectrometry analysis and N-terminal sequencing were carried out on the purified enzyme.

**Cell lysate preparation and Western blot analysis**

Overnight cultures of *S. aureus* were freshly diluted in BHI and were grown until mid-exponential phase (OD_600nm_=0.3-0.4). At this point CdCl_2_ (10 μM) was added to induce protein expression and the cells were left to grow further. Samples were collected at the indicated time-points to evaluate levels of RNase III expression. Cells were suspended in lysis buffer (30 mM Tris pH 8.0, 300 mM NaCl, 10 mM imidazole, Triton X-100 0.25%), lysed with lysostaphin and treated with DNase I (0.0125 u/μl). Cell lysates were centrifuged at 13,500 rpm for 30 min and supernatants were kept separately. Biorad analysis followed and 40 µg of each sample were analyzed on an SDS-PAGE gel. Following transfer of proteins on a PVDF membrane (Millipore) the upper half of the membrane was cut and removed to prevent interaction of antibody with Protein A. Incubation with an anti-Flag antibody (Sigma, F1804) and Western blot procedure took place according to manufacturer’s instructions.

**Northern blot and measurement of mRNA half-life**

RNA was isolated from various stages of growth for Northern analysis. For measurement of RNA half-life, cultures in exponential growth phase (OD_600nm_ 0.2-0.3) were treated with rifampicin (300 μg/ml). Samples were collected at the indicated time-points and RNAs were extracted according to the FastRNA Pro protocol (Qbiogene). After separation on agarose gels (1-2 %) containing 20 mM guanidine thiocyanate [3] or 8 % polyacrylamide- 8 M urea gels, RNA was transferred onto Hybond-N+ membranes (Amersham). For detection of transcripts, γ-ATP labeled oligodeoxyribonucleotides, DIG-labeled RNA or DNA probes (prepared according to the protocol provided by Roche) were used (Table S8). Each experiment was reproduced at least three times.

**Determination of mRNA 5’ ends by primer extension and RACE experiments**

Primer extension was carried out on total RNA isolated from various phases of growth. In brief, 10-40 μg of total RNA extracts were mixed with γ-ATP labeled primer, precipitated, washed and suspended in 1X Avian Myeloblastosis Virus reverse transcriptase (AMV, Qbiogene) buffer. The RNA and primer were denatured and then annealed at 54°C for 30 min. Reverse transcription was carried at 45°C for 1h in the presence of 10 units of AMV, 8 units of RNasine (Promega) and 0.5 mM dNTPs. After the primer extension reaction, the RNA was hydrolyzed and the cDNA was precipitated, washed and suspended in DNA loading dye as described [4]. The cDNA fragments were fractionated on an 8% polyacrylamide gel. Sequencing reactions on *in vitro* transcribed RNA fragments were run in parallel as previously described [4].

To assign the 5’ and 3’ ends of the RNAs, the RACE approach was done on previously circularized RNA using T4 RNA ligase [5] (Table S7).

**RNA substrate preparation**

*In vitro* transcription reactions of RNA fragments were done using home-made T7 phage RNA polymerase. After transcription, RNAs were treated with DNase I and were purified from a polyacrylamide gel [4]. PCR fragments were used as templates for transcription where the T7 recognition sequence was incorporated into one of the primers (listed in Table S8). The following sets of primers were used to prepare the RNA fragments: 404/279 (16S rRNA), 170/115 (FL-*rnc*) 171/115 (Δ5’UTR-*rnc*), 286/16 (*cspA-L*), 344/16 (*cspA-S*), 417/418 (*as-cspA*), 270/71 (hu), 370/371 (*as-hu*), 291/292 (*secY*), 331/221 (*tagG*), 333/384 (*tagH*), 247/248 (tRNA-Tyr-tRNA-Gln), 251/252 (tRNA-Arg), 273/307 (6S RNA-SAS050), 263/264 (FMN riboswitch), 288/289 (4.5S RNA), 261/262 (SAS025), 359/364 (RsaA), and 502/503 (SA2097). For transcription of *spa*, plasmid pUT7-*spa* was used (Table S6). In the case where DIG-labeled RNA fragments were prepared, the protocol recommended by Roche was followed.

The 5' end-labeling of dephosphorylated RNA transcripts or DNA oligonucleotides was performed with T4 polynucleotide kinase and [γ-^32^P]ATP [4]. Before use, RNAs were denatured by incubation at 90°C for 2 min in the absence of magnesium and salt, then chilled on ice for 1 min, followed by a renaturation step at 20°C for 15 min in TMK buffer (20 mM Tris-acetate pH 7.5, 10 mM magnesium-acetate, 150 mM KCl).

Complex formation between sense and antisense RNAs was formed under denaturing and native conditions. Under “native conditions”, the two RNAs were first denatured and renatured separately as described above. Subsequently, the 5’ end-labeled or unlabeled RNA (3 10^-8^ M) was mixed with a fivefold excess of unlabeled antisense RNA and the hybrid was allowed to form at 37°C for 1-15 min in TMK buffer. Under “denaturing conditions”, full duplexes were formed by incubation of the two RNAs at 90°C for 2 min followed by slow cooling to 37°C in TMK buffer.

**RNase III cleavage assay**

The RNase III cleavage assay was done as described previously [3]. Briefly, the renatured and unlabeled RNA fragment (100-200 nM) is incubated with purified RNase III (0.165-0.66 μM) in the presence of Mg^2+^ or Ca^2+^ (TMK or TCK buffer, respectively; 10X TMK buffer consists of 100 mM Tris-HCl, pH 8, 100 mM MgCl_2_, 1 M KCl) for 5 min at 37°C. Reactions also contain 1 mM DTT and 1 μg of yeast total tRNA (Sigma). RNA fragments are subsequently precipitated and loaded directly on a denaturing agarose or polyacrylamide gel and visualized by ethidium bromide staining.

To assign the RNase III cleavages on an unlabeled RNA fragment, we used primer extension. The cleaved RNA was mixed with a 5’-labeled oligonucleotide (0.4 pmol) and the sample was heated at 90°C for 1 min, cooled on ice and then incubated at 20°C for 15 min in the RT buffer containing 50 mM Tris-HCl pH 7.5, 20 mM MgCl_2_, 50 mM KCl. Reverse transcription was subsequently performed with 0.3 mM dNTPs and 0.1 U/µl RT (AMV) in RT buffer at 37°C for 30 min. The RNA template was then destroyed by addition of 0.5 M KOH and 1 volume 50 mM Tris-HCl pH 8, 0.5 % SDS, 7.5 mM EDTA and incubation at 90°C for 3 min followed by 1h at 37°C. RNA was precipitated with 0.4 M acetic acid, 0.3 M Na-acetate and 3 volumes ethanol. Samples were run on a 8% polyacrylamide-7 M urea gels and analyzed by autoradiography. Sequencing reactions on purified RNA transcripts were run in parallel.

When 5’ end-labeled RNA was used, the cleavages were assigned by using an RNase T1 reaction under denaturing conditions and an alkaline ladder [4]. The samples were fractionated using 12% polyacrylamide gel-7 M urea gels and analyzed by autoradiography.

**Enzymatic structure probing**

Determination of the RNA secondary structure was performed by enzymatic hydrolysis of unlabeled RNA transcripts according to Benito et al. [6]. Enzymatic hydrolysis was performed in 10 µl of TMK at 37°C for 5 min, in the presence of 1 µg carrier tRNA and RNase T1 (0.0025 U), RNase V1 (0.1 U), or RNase T2 (0.05 U). Reactions were stopped by phenol/chloroform extraction, followed by ethanol precipitation, and washing with 80% ethanol. Incubation controls were done in parallel to detect nicks in the RNA. The cleavage sites were assigned by primer extension as described above.

**Toeprinting assay**

The formation of a simplified translational initiation complex with mRNA and the extension inhibition conditions was done according to [7]. The mRNA was first annealed to the 5’ end-labeled oligonucleotide in 20 mM Tris-acetate pH 7.5, 60 mM NH_4_Cl, 3 mM ß-mercaptoethanol, heated at 90°C for 1 min and put on ice for 1 min. Mg-acetate was added at 10 mM final concentration and incubation was continued at 20°C for 20 min. Initiation complex formation was performed at 37°C for 15 min in a reaction mixture containing 10 mM Tris-acetate pH 7.4, 60 mM NH_4_Cl, 10 mM Mg-acetate, 6 mM ß-mercaptoethanol in the presence of 4 nM mRNA annealed to the labeled primer, 0.1-0.5 µM 30S subunits and 1 µM tRNA^fMet^.

Primer extension reactions were subsequently performed by adding 2-4 units of AMV reverse transcriptase and incubation at 37°C for 15 min. Reactions were stopped by phenol extraction and ethanol precipitation. Samples were heated at 90°C for 3 min, and gel electrophoresis was done on 8% polyacrylamide-8 M urea slab gels. Quantifications of the data were done by Bioimager analysis of dried gels. Relative toeprints were calculated by relating the intensity of the particular band corresponding to the +16 RT pause (toeprint) to the sum of the intensities of this band and the band corresponding to the full length RNA.

**Analysis of complex formation using band-shift experiments**

To perform band-shift assays 50-100 nM of unlabeled transcript were incubated 5 min at 37°C in the presence of increasing concentrations of the mutated RNase III-E135A, and 1 µg of cold tRNA in the TCK buffer under conditions identical to those described for the cleavage assays. At the end of the binding reaction 6X loading dye (30% glycerol, 0.25% bromophenol blue and 0.25% xylene cyanol) was added to the samples and they were analyzed on a 2% agarose gel in 0.5X TBE buffer. RNA-protein complexes were then transferred on a Hybond-N+ membrane using a vacuum pump over 2h. Subsequently, the membranes were hybridized with 5’-^32^P-end-labeled oligonucleotide as for Northern blot hybridization.

Experiments were also carried out with end-labeled *cspA_L_* mRNA transcript previously renatured in the TCK buffer. The labeled mRNA was incubated with RNase III-E135A (200-800 nM) in the presence of cold competitor RNA (from 10 nM to 500 nM) for 5 min at 37°C. The samples were loaded on 5% or 8% polyacrylamide gel under non denaturing conditions and the data were analyzed after autoradiography.

**References**

1. Huntzinger E, Boisset S, Saveanu C, Benito Y, Geissmann T, et al. (2005) *Staphylococcus aureus* RNAIII and the endoribonuclease III coordinately regulate *spa* gene expression. EMBO J 24: 824-835.

2. Charpentier E, Anton AI, Barry P, Alfonso B, Fang Y, et al. (2004) Novel cassette-based shuttle vector system for gram-positive bacteria. Appl Environ Microbiol 70: 6076-6085.

3. Chevalier C, Huntzinger E, Fechter P, Boisset S, Vandenesch F, et al. (2008) *Staphylococcus aureus* endoribonuclease III purification and properties. Methods Enzymol 447: 309-327.

4. Chevalier C, Geissmann T, Helfer AC, Romby P (2009) Probing mRNA structure and sRNA-mRNA interactions in bacteria using enzymes and lead(II). Methods Mol Biol 540: 215-232.

5. Redko Y, Bechhofer DH, Condon C (2008) Mini-III, an unusual member of the RNase III family of enzymes, catalyses 23S ribosomal RNA maturation in *B. subtilis*. Mol Microbiol 68: 1096-1106.

6. Benito Y, Kolb FA, Romby P, Lina G, Etienne J, et al. (2000) Probing the structure of RNAIII, the *Staphylococcus aureus agr* regulatory RNA, and identification of the RNA domain involved in repression of protein A expression. RNA 6: 668-679.

7. Fechter P, Chevalier C, Yusupova G, Yusupov M, Romby P, et al. (2009) Ribosomal initiation complexes probed by toeprinting and effect of trans-acting translational regulators in bacteria. Methods Mol Biol 540: 247-263.

8. Abu-Qatouseh LF, Chinni SV, Seggewiss J, Proctor RA, Brosius J, et al. (2010) Identification of differentially expressed small non-protein-coding RNAs in *Staphylococcus aureus* displaying both the normal and the small-colony variant phenotype. J Mol Med (Berl) 88: 565-575.

9. Beaume M, Hernandez D, Farinelli L, Deluen C, Linder P, et al. (2010) Cartography of methicillin-resistant *S. aureus* transcripts: detection, orientation and temporal expression during growth phase and stress conditions. PLoS One 5: e10725.

10. Geissmann T, Chevalier C, Cros MJ, Boisset S, Fechter P, et al. (2009) A search for small noncoding RNAs in *Staphylococcus aureus* reveals a conserved sequence motif for regulation. Nucleic Acids Res 37: 7239-7257.

11. Kreiswirth BN, Löfdahl S, Betley MJ, O'Reilly M, Schlievert PM, et al. (1983) The toxic shock syndrome exotoxin structural gene is not detectably transmitted by a prophage. Nature 305: 709-712.

12. Nagata M, Kaito C, Sekimizu K (2008) Phosphodiesterase activity of CvfA is required for virulence in *Staphylococcus aureus*. J Biol Chem 283: 2176-2184.

13. Novick RP (1963) Analysis by transduction of mutations affecting penicillinase formation *in Staphylococcus aureus*. J Gen Microbiol 33: 121-136.

14. Peng HL, Novick RP, Kreiswirth B, Kornblum J, Schlievert P (1988) Cloning, characterization, and sequencing of an accessory gene regulator (*agr*) in *Staphylococcus aureus*. J Bacteriol 170: 4365-4372.

15. Pichon C, Felden B (2005) Small RNA genes expressed from *Staphylococcus aureus* genomic and pathogenicity islands with specific expression among pathogenic strains. Proc Natl Acad Sci U S A 102: 14249-14254.

16. Roberts C, Anderson KL, Murphy E, Projan SJ, Mounts W, et al. (2006) Characterizing the effect of *Staphylococcus aureus* virulence factor regulator, SarA, on log-phase mRNA half-lives. J Bacteriol 188: 2593-2603.

17. Abu-Qatouseh LF, Chinni SV, Seggewiss J, Proctor RA, Brosius J, et al. (2010) Identification of differentially expressed small non-protein-coding RNAs in *Staphylococcus aureus* displaying both the normal and the small-colony variant phenotype. J Mol Med (Berl) 88: 565-575.

18. Beaume M, Hernandez D, Farinelli L, Deluen C, Linder P, et al. (2010) Cartography of methicillin-resistant *S. aureus* transcripts: detection, orientation and temporal expression during growth phase and stress conditions. PLoS One 5: e10725.

19. Pichon C, Felden B (2005) Small RNA genes expressed from *Staphylococcus aureus* genomic and pathogenicity islands with specific expression among pathogenic strains. Proc Natl Acad Sci U S A 102: 14249-14254.

20. Do CB, Woods DA, Batzoglou S (2008) CONTRAfold: RNA secondary structure prediction without physics-based models. Bioinformatics 22: e90-98.

21 Lorenz R, Bernhart SH, Höner Zu Siederdissen C, Tafer H, Flamm C, Stadler PF, Hofacker IL (2011) ViennaRNA Package 2.0. Algorithms Mol Biol. 6: 26.
